# Evolutionary diversification of the canonical Wnt signaling effector TCF/LEF in chordates

**DOI:** 10.1111/dgd.12771

**Published:** 2022-02-03

**Authors:** Nuria P. Torres‐Aguila, Marika Salonna, Stefan Hoppler, David E. K. Ferrier

**Affiliations:** ^1^ Gatty Marine Laboratory The Scottish Oceans Institute School of Biology University of St Andrews St Andrews UK; ^2^ Institute of Medical Sciences University of Aberdeen Aberdeen UK

**Keywords:** amphioxus, *Ciona*, comparative genomics, cyclostome, lamprey

## Abstract

Wnt signaling is essential during animal development and regeneration, but also plays an important role in diseases such as cancer and diabetes. The canonical Wnt signaling pathway is one of the most conserved signaling cascades in the animal kingdom, with the T‐cell factor/lymphoid enhancer factor (TCF/LEF) proteins being the major mediators of Wnt/β‐catenin‐regulated gene expression. In comparison with invertebrates, vertebrates possess a high diversity of TCF/LEF family genes, implicating this as a possible key change to Wnt signaling at the evolutionary origin of vertebrates. However, the precise nature of this diversification is only poorly understood. The aim of this study is to clarify orthology, paralogy, and isoform relationships within the TCF/LEF gene family within chordates via in silico comparative study of TCF/LEF gene structure, molecular phylogeny, and gene synteny. Our results support the notion that the four TCF/LEF paralog subfamilies in jawed vertebrates (gnathostomes) evolved via the two rounds of whole‐genome duplications that occurred during early vertebrate evolution. Importantly, gene structure comparisons and synteny analysis of jawless vertebrate (cyclostome) *TCF*s suggest that a *TCF7L2‐*like form of gene structure is a close proxy for the ancestral vertebrate structure. In conclusion, we propose a detailed evolutionary path based on a new pre‐whole‐genome duplication vertebrate *TCF* gene model. This ancestor gene model highlights the chordate and vertebrate innovations of TCF/LEF gene structure, providing the foundation for understanding the role of Wnt/β‐catenin signaling in vertebrate evolution.

## Graphical abstract

The canonical Wnt/β‐catenin signaling pathway acts through the TCF/LEF family of transcription factors. The step change in diversity of the genes in this family across the invertebrate–vertebrate transition is described here, via a new evolutionary model that includes the inferred intermediate genes in this transition. This provides a foundation for future work on the functional diversity of these genes in development and disease.

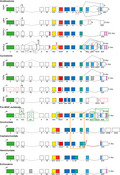

## 1 INTRODUCTION

Wnt signaling is a cell‐to‐cell signaling mechanism highly conserved in the animal kingdom (Hoppler & Moon, 2014). It is necessary during development and regeneration, but is also often found to be defective in different diseases, such as cancer and diabetes (Nusse & Clevers, 2017). Extracellular Wnt ligands bind to cell surface receptors to activate several intracellular signal transduction pathways. The best‐described such Wnt pathway is the canonical Wnt/β‐catenin pathway, in which extracellular Wnt signaling promotes translocation of β‐catenin protein into the cell nucleus to function as a transcriptional co‐regulator. Several different DNA‐binding factors have been identified that interact with nuclear β‐catenin to regulate Wnt‐target gene transcription (Mukherjee et al., 2020; Zimmerli et al., 2020). However, T‐cell factor/lymphoid enhancer factor (TCF/LEF) proteins are the major mediators of Wnt/β‐catenin‐regulated gene expression in the nucleus, with widespread roles in development and human disease (Cadigan & Waterman, 2012; Hoppler & Waterman, 2014; Mayer et al., 2020; Söderholm & Cantù, 2021).

Members of the TCF/LEF protein family are renowned for acting as bimodal transcription factors (Ramakrishnan & Cadigan, 2017). In general, without Wnt signaling (with low levels of nuclear β‐catenin) they act as transcriptional repressors, but when Wnt signaling is present (with high levels of nuclear β‐catenin) they act as transcriptional activators. This bimodal activity is functionally mediated through different protein domains and motifs in the coding sequence of TCF/LEF genes (Cadigan & Waterman, 2012; Hoppler & Waterman, 2014). Four main domains can be defined: (a) the β‐catenin binding domain (BCBD, located at the N‐terminus), (b) a co‐repressor binding domain, (c) the High Mobility Group box (HMG box, DNA‐binding domain, together with a nuclear localization signal [NLS] motif), and (d) a C‐clamp domain (an additional DNA‐binding domain, located at the C‐terminus). Additional motifs are present in some TCF/LEF protein isoforms, including an LVPQ motif, an SxxSS motif, and a C‐terminal binding protein (CtBP) motif. While the β‐catenin binding domain (BCBD) is required for the transcriptional activator activity (Behrens et al., 1996), the co‐repressor binding domain and the LVPQ, SxxSS, and CtBP motifs are associated with the transcriptional repressor activity (Brantjes et al., 2001; Liu et al., 2005; Valenta et al., 2003).

Genome comparisons between vertebrates and invertebrates reveal a remarkable conservation of the canonical Wnt signaling pathway, with relatively little expansion of most Wnt pathway component genes in general (Croce & Holstein, 2014; but see also Gray et al., 2009 for an exception). However, a much greater diversity of vertebrate TCF/LEF transcription factors is observed (Cadigan & Waterman, 2012; Hoppler & Kavanagh, 2007; Hoppler & Waterman, 2014; Mao & Byers, 2011). It has been proposed that multiple copies of TCF/LEF genes have been retained from genome duplications in vertebrates, which typically are thought to possess four TCF/LEF family genes with multiple isoforms in most vertebrates such as humans, while invertebrates are believed to typically have one *TCF* gene with a single isoform (Hoppler & Waterman, 2014). Among all this vertebrate genetic diversity, it has been shown that vertebrate TCF/LEF paralogs have some degree of redundancy at the functional level (Hoppler & Kavanagh, 2007). Nevertheless, the diversity of vertebrate TCF/LEF genes, and the isoforms produced from them, presumably reflects a wide array of functional capabilities downstream of canonical Wnt signaling in vertebrates, and some degree of functional differences between vertebrate genes is already known (Liu et al., 2005; reviewed in Arce et al., 2006), but this is poorly understood at present. Despite vertebrate and invertebrate TCF/LEF genes sharing some inherited ancestral structures and functions, it is still unknown to what extent these characteristics are either shared or have been apportioned among vertebrate paralogs, leading to redundancy, sub‐functionalization, and/or neofunctionalization within this gene family. Thus, this diversity in vertebrate TCF/LEF genes is likely key to understanding the evolution of vertebrate Wnt signaling, and therefore possibly many aspects of vertebrate evolution itself.

Due to the presence of four TCF/LEF genes in the genome of vertebrates, it has been assumed that these originated from a single *TCF* gene ancestor through the two rounds of whole‐genome duplications (2R WGD) that occurred at the base of vertebrate evolution (Aase‐Remedios & Ferrier, 2021; Holland & Ocampo Daza, 2018; Lamb, 2021). However, this hypothesis is currently based only on phylogenetic analyses that include a limited number of gnathostome species (Klingel et al., 2012; Zhu et al., 2021). In this study, we undertake an in silico comparative study of *TCF* gene structure across deuterostomes, with a focus on chordates and the invertebrate–vertebrate transition, using molecular phylogeny and synteny analyses to clarify orthology relationships within this gene family. We investigated a total of 37 species, which include 19 gnathostomes (jawed vertebrates), 5 cyclostomes (agnathans or jawless vertebrates), 5 urochordates (tunicates), 3 cephalochordates (lancelets), 3 echinoderms, and 2 hemichordates. This diversity of species outside the gnathostome clade sheds light on *TCF* evolution at the very base of the vertebrates, from the last common ancestor of chordates via the pre‐WGD last common ancestor of vertebrates to the four subfamilies we find in vertebrates.

## 2 MATERIALS AND METHODS

### 2.1 Phylogenetic analysis

Orthologous TCF/LEF genes were searched for across 24 different vertebrate species and 13 invertebrate species. Each ortholog was identified by reciprocal tBLASTn (BLOSUM 64) against species genome assemblies available on Ensembl or the National Center for Biotechnology Information (NCBI), or the Squalomix portal for *Eptatretus burgeri* (https://transcriptome.riken.jp/squalomix/) (Hara et al., 2018); *Mus musculus* and *Danio rerio* protein sequences were used as start queries. Additionally, available transcriptomic data from *Petromyzon marinus* (SRX9248557–SRX9248631), *Lampetra planeri* (SRX2539407), and *Branchiostoma lanceolatum* (Marlétaz et al., 2018) were used to refine the respective gene models. Detailed gene information for each species with notes on how various gene models were manually curated and final protein isoform sequences inferred is shown in Appendix S1.

Due to the presence of different isoforms, the longest isoform was selected for the subsequent multiple alignments and molecular phylogenetic analyses. The amino‐acid sequences of each group of gnathostome paralogs (*TCF7*, *LEF1*, *TCF7L1*, *TCF7L2*) and invertebrate TCFs were aligned separately with the MUSCLE algorithm, and a posterior manual exon‐level editing was performed, at which point the cyclostome sequences were also incorporated. The five distinct alignments were merged into one, and a final step of manual editing of the alignment was performed. This alignment (Appendix S2) was used as input for the phylogenetic tree building. The maximum likelihood phylogenetic tree was made using IQ‐TREE 1 software (Kalyaanamoorthy et al., 2017; Nguyen et al., 2015) with the parameter “‐m MFP,” which tests and selects a model based on tree inferences, and the parameter “‐b” set at 1,000, to perform bootstrapping with 1,000 replicates. The TCF sequence of the hemichordates *Ptychodera flava* and *Saccoglossus kowalevskii* and echinoderms *Strongylocentrotus purpuratus*, *Asterias rubens*, and *Apostichopus japonicus* were set as outgroup sequences to root the tree. The Bayesian phylogeny was constructed with MrBayes version 3.2.7 (Ronquist & Huelsenbeck, 2003) with two runs for up to 5 × 10^8^ generations and a stoprule with a standard deviation of split frequencies set to 0.01. The two runs did not converge (standard deviation of split frequencies never reached below 0.06 and ended at 0.08), so the analysis was ended after 1.34 × 10^7^ generations. Trees from each run were summarized separately with TreeAnnotator version 1.10.1 (Suchard et al., 2018) to build maximum clade credibility (MCC) trees revealing that each run had settled on a different topology. Since these Bayesian phylogenies did not converge, they do not allow any clear resolution of the relationship of the cyclostome and gnathostome paralogy groups, and the trees are thus not shown.

### 2.2 Synteny analysis

Comparisons of synteny of *TCF7L1* genes and their neighbors in teleosts were performed using the web tool Genomicus (Nguyen et al., 2018) to identify shared neighboring genes, with further confirmation by tBLASTn (BLOSUM 64).

Synteny analysis within lampreys was performed by ultra‐fast genome comparison using the chromeister software (Pérez‐Wohlfeil et al., 2019) to identify homologous chromosomes between species, focusing on the chromosomes that have *TCF* genes. The sequences of all *P. marinus* chromosomes were compared against the sequences of all *Entosphenus tridentatus* and *Lethenteron reissneri* chromosomes.

The synteny analysis between lampreys and gnathostomes was based on the chordate linkage groups described previously (Simakov et al., 2020). We focus on the genes that belong to the ancestral chordate linkage group that contained the ancestral pre‐WGD *TCF* gene, the Chordate Linkage Group Q (CLGQ). Using as reference the CLGQ and the genomes of four gnathostomes (*Mus musculus*, *Gallus gallus*, *Xenopus tropicalis*, *Lepisosteus oculatus*), each gene of the CLGQ was used to search by reciprocal BLAST each gnathostome genome, and genes were defined as a TCF/LEF neighborhood gene if they were located in the same chromosome as any of the TCF/LEF genes in at least one genome. We defined independent gene neighborhoods for each *TCF* paralog (*LEF1* neighborhood, LEF1nbh; *TCF7* neighborhood, TCF7nbh; *TCF7L1* neighborhood, TCF7L1nbh; *TCF7L2* neighborhood, TCF7L2nhb). Secondly, each CLGQ gene was searched for in the *P. marinus* genome and its chromosome location recorded. A binomial test was used to identify whether there was a nonrandom distribution of orthologs of CLGQ genes across the *P. marinus* chromosomes. Also, a Barnard’s test was used to identify overrepresentation of TCF/LEF neighborhoods on selected chromosomes. Both statistical analyses were performed in R version 3.6.1, using the function binom.test() from the “stats” package and binom.test() from the “Barnard” package.

## 3 RESULTS

### 3.1 Comparative analysis of TCF/LEF gene numbers in deuterostomes

Among gnathostomes, we have confirmed in all 19 species studied the previously expected four *TCF* paralogous subfamilies (*TCF7*, *LEF1*, *TCF7L1*, and *TCF7L2*), consistent with the 2R WGD hypothesis, usually with one single paralog for each TCF subfamily (Table 1). There are, however, three departures from this one‐paralog per *TCF* subfamily standard.

**TABLE 1:** Number of TCF/LEF genes found on each studied vertebrate species

**Gnathostome species**	***LEF1***	***TCF7***	***TCF7L1***	***TCF7L2***	**Total**
*Homo sapiens* (human)	1	1	1	1	4
*Mus musculus* (house mouse)	1	1	1	1	4
*Gallus gallus* (modern‐day chicken)	1	1	1	1	4
*Taeniopygia guttata* (zebra finch)	1	1	1	1	4
*Anolis carolinensis* (green anole)	1	1	1	1	4
*Chrysemys picta bellii* (Chinese turtle)	1	1	1	1	4
*Xenopus tropicalis* (western clawed frog)	1	1	1	1	4
*Xenopus laevis* (African clawed frog)	2	1	2	2	7
*Danio rerio* (zebrafish)	1	1	2	1	5
*Oryzias latipes* (medaka)	1	1	2	1	5
*Tetraodon nigroviridis* (green spotted pufferfish)	1	1	2	1	5
*Takifugu rubripes* (Japanese pufferfish)	1	1	2	1	5
*Latimeria chalumnae* (coelacanth)	1	1	1	1	4
*Acipenser ruthenus* (sterlet)	2	2	2	2	8
*Lepisosteus oculatus* (spotted gar)	1	1	1	1	4
*Amblyraja radiata* (starry ray)	1	1	1	1	4
*Chiloscyllium palgiosum* (bamboo shark)	1	1	1	1	4
*Scyliorhinus canicula* (small‐spotted catshark)	1	1	1	1	4
*Callorhinchus milii* (elephant shark)	1	1	1	1	4

Cyclostome species	*TCFa*	*TCFb*	*TCFc*	*TCFd*	Total
*Petromyzon marinus* (sea lamprey)	1	1	1	0^a^	3
*Lethenteron reissneri* (Far Eastern brook lamprey)	1	1	0^a^	0^a^	2
*Entosphenus tridentatus* (Pacific lamprey)	1	1	0^a^	0^a^	2
*Lampetra planeri* (brook lamprey)	1	1	1	1	4
*Eptatretus burgeri* (inshore hagfish)	1	1	0^a^	1	3

^a^ No signs of the gene in the current genome assembly and/or transcriptomic data.

**FIGURE 1:**
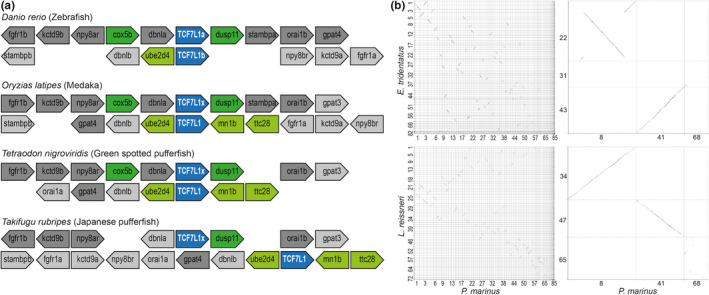
Synteny analysis. (a) Representation of Genomicus synteny results of *TCF7L1* paralogs in teleosts. Blue, TCF7L1 gene. Dark/light gray, genes with paralogy relationships comparable to *TCF7L1a/b* paralogy; the dark‐gray genes are those in the *D*. *rerio TCF7L1a* paralogon, and light gray in the *D*. *rerio TCF7L1b* paralogon. Dark green, genes whose locations are conserved within *TCF7L1a* paralogons. Light green, genes whose locations are conserved within *TCF7L1b* paralogons. (b) Dot plots of genomic sequence comparison between *P*. *marinus* and *E*. *tridentatus* and *L. reissneri*. Left panels show genome versus genome comparison; right panels show *TCF*‐containing chromosome comparisons. Axes indicate chromosome number

**FIGURE 2:**
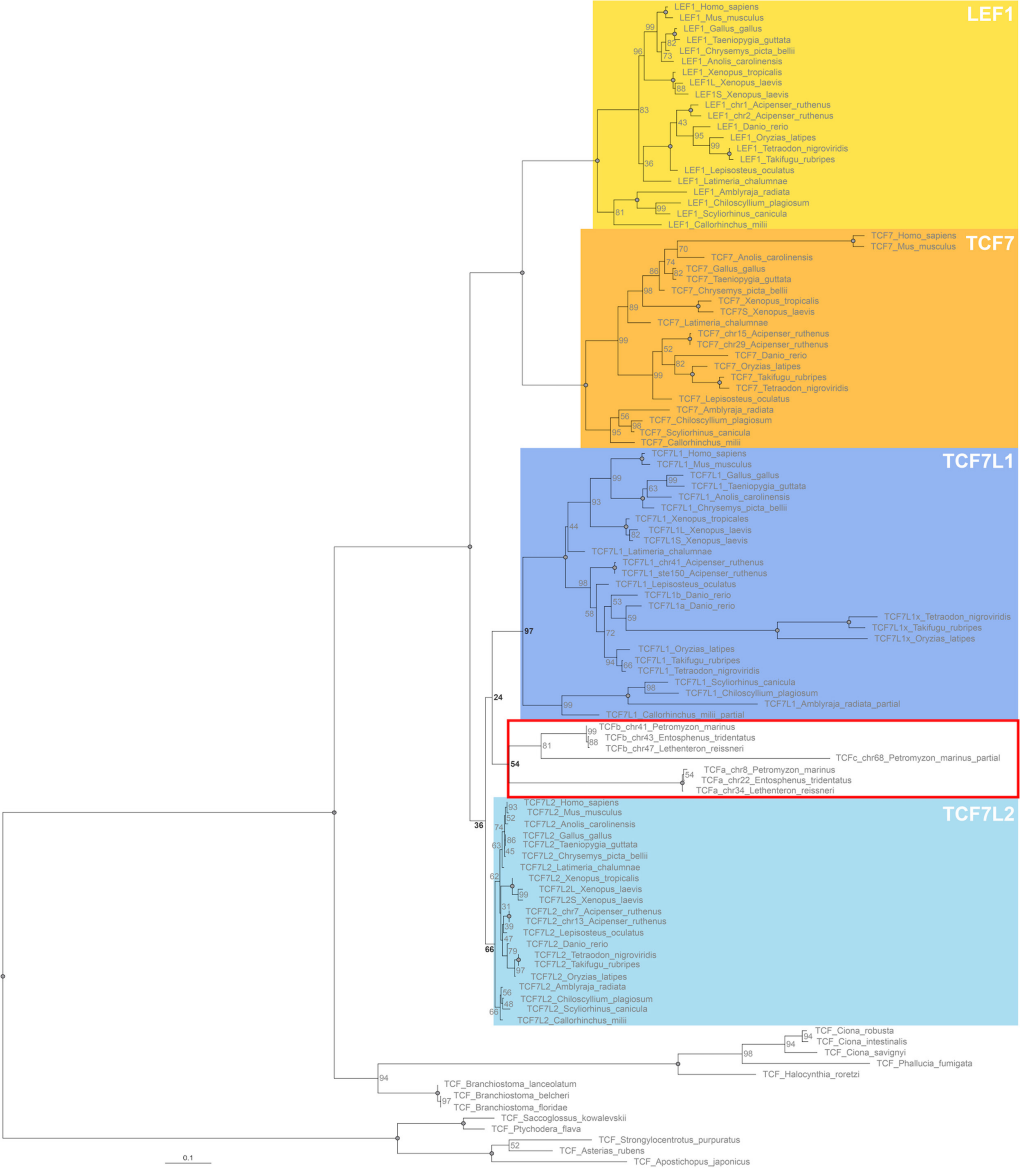
Phylogenetic tree of TCF/LEF family genes. Orange, yellow, dark‐blue, and light‐blue boxes highlight *TCF7*, *LEF1*, *TCF7L1*, and *TCF7L2* ohnolog subfamilies respectively. Red square highlights the cyclostome gene clade. Node values are maximum‐likelihood percentage bootstrap support. The circles in the node indicate a support value of 100

One such exception are the teleost fish, which have one extra copy of *TCF7L1* (Dorsky et al., 2003). We hypothesized that this results from the third round of whole genome duplication (3R WGD) previously described in this teleost lineage (Meyer & Van de Peer, 2005), with secondary gene loss after the 3R WGD accounting for the return of the other *TCF* subfamilies to the single paralog state. We confirmed that the paralogy of the two teleost *TCF7L1* genes likely arose via a large‐scale duplication event (such as the 3R WGD) by observing conserved synteny between *TCF7L1* gene neighborhoods in each teleost genome with, for example, the *dbnla* and *dbnlb* paralogs or fgfr1b and fgfr1a paralogs mirroring the *TCF7L1* relationship (Figure 1a). Furthermore, we found that the flanking genes to the previously unnamed *TCF7L1* paralogs in *Oryzias latipes* (ENSORLG00000022421), *Tetraodon nigroviridis* (ENSTNIG00000013604), and *Takifugu rubripes* (ENSTRUG00000018634) (TCF7L1x in Figure 1a) were the same as the closest chromosomal gene neighbors to *Danio rerio TCF7L1a* (also known as *headless* in zebrafish; Kim et al., 2000), thus unambiguously identifying the previously named *TCF7L1* in *O*. *latipes*, *T. nigroviridis*, and *T*. *rubripes* as orthologous to *D*. *rerio TCF7L1b*, while the unnamed paralogs are *TCF7L1a* genes (Figure 1a), providing greater resolution than the phylogenetic signal alone (see below and Figure 2).

The second and third exceptions to the one‐paralog‐per‐*TCF*‐subfamily standard in gnathostomes are the amphibian *Xenopus laevis* and the bony fish *Acipenser ruthenus*. Both species underwent two independent 3R WGDs (Du et al., 2020; Uno et al., 2013), and have seven and eight *TCF* genes, respectively, with two paralogs in each *TCF* subfamily, except for *X*. *laevis* with only one *TCF7* paralog (*tcf7.S*) (Table 1). The absence of the corresponding *tcf7.L* from the *X*. *laevis* genome was confirmed by synteny identifying an absence of a *Tcf* gene from the paralogous “L” neighborhood.

In the case of cyclostomes, we identified three *TCF* genes in the genome of *P. marinus*, each supported by transcriptomic data, which we call *TCFa* (on chromosome 8)*, TCFb* (on chromosome 41), and *TCFc* (on chromosome 68). In transcriptomic data of *L*. *planeri,* we identified three genes similar in sequence to *TCFa*, *TCFb*, and *TCFc*, and a fourth putative *TCF* gene that we named *TCFd*. However, only two *TCF* genes were detected in the genomic data of *Entosphenus tridentatus* and *Lethenteron reissneri*. For *Eptatretus burgeri,* despite the fact that its genome is not assembled to the level of chromosomes, we were able to identify three TCF genes (Table 1). The amino‐acid sequences of the *E*. *tridentatus* and *L*. *reissneri* TCFs and two of the *E*. *burgeri* genes are highly similar to TCFa and TCFb of *P. marinus*, while the third *E*. *burgeri* TCF is similar to *L*. *planeri* TCFd (Figure S1a).

To check if *TCFc* might be missing due to gaps in the respective genome assemblies, we performed whole‐genome synteny comparisons. We were able to identify homologous chromosomes for *P. marinus* chromosome 8 and chromosome 41 in both *E*. *tridentatus* and *L*. *reissneri*, which contain *TCFa* and *TCFb*, respectively. In contrast, no chromosome homologous to *P*. *marinus* chromosome 68 (which contains *TCFc*) was clearly identified, with a high level of rearrangement of the components of this chromosome across other lamprey genomes (Figure 1b). This then precludes the unambiguous detection of a syntenic genomic region in these lamprey species that is orthologous to the genomic neighborhood containing *P. marinus TCFc*. With current data it is thus not possible to assess whether there is a gap in the respective assemblies at the location where a *TCFc* gene might be expected to be located, and hence we cannot definitively conclude whether orthologs of this gene are present in these lamprey genomes or not. No genome comparisons with *E*. *burgeri* or *L*. *planeri* were performed because of the current lack of chromosome‐level assemblies.

Nevertheless, according to the lamprey phylogeny (Potter et al., 2015), *P. marinus* is the earliest branching (most basal) lineage of those lamprey species sequenced, and the clade of *L*. *planeri* and *L*. *reissneri* arose later (higher) in the phylogeny. Thus, our results suggest an ancestral four *TCF* paralog status for cyclostomes followed by secondary losses of *TCFc* and *TCFd* from some species due to the presence of a *TCFc*‐like sequence in the *L*. *planeri* transcriptome that appears orthologous to the *TCFc* of *P. marinus*, as well as *TCFd* orthologs in the hagfish *E. burgeri* and lamprey *L. planeri* (Figure S1b).

Beyond vertebrates (both the gnathostomes and cyclostomes), our analysis in closely related invertebrates (chordates and more widely in some deuterostomes) confirms the presence of only one *TCF* gene in all 13 invertebrate genomes examined. Our detailed analysis of the numbers of TCF/LEF genes per genome is therefore consistent with the origin of four gnathostome paralogs (ohnologs) from a single *TCF* gene ancestor through the two rounds of whole‐genome duplications (2R WGD) early in vertebrate evolution.

### 3.2 Phylogenetic analysis of the TCF/LEF family in deuterostomes

A phylogenetic tree was made with the protein sequence alignment of a total of 107 sequences from 22 vertebrate and 13 invertebrate species (Figure 2, Appendix S2). The TCF sequences from *E*. *burgeri* and *L*. *planeri* were not included because they currently lack some important domains owing to only partial sequences of the genes being available. The phylogeny of gnathostome sequences shows a pattern consistent with the vertebrate 2R‐WGD, with the TCF7 and LEF1 subfamilies as sister groups in one clade and the TCF7L1 and TCF7L2 subfamilies as sisters in a second clade. This pattern is particularly clear with trees built with gnathostome subfamilies alone (Figure S2). Intriguingly, cyclostome TCFs might form a group located within the TCF7L1/L2 clade. However, this clade has a node support value of only 36% in the maximum‐likelihood phylogeny (Figure 2) and is unresolved in Bayesian phylogenies (data not shown). Thus, the phylogenetic position of the cyclostome genes relative to those of gnathostomes is poorly resolved. As expected, the urochordate and cephalochordate TCFs are located as basal branches in the chordate clade, as a sister group to the vertebrate TCF/LEF clade. Echinoderm and hemichordate sequences were used to root the tree.

### 3.3 A comparative analysis of TCF/LEF gene structures in deuterostomes

**FIGURE 3:**
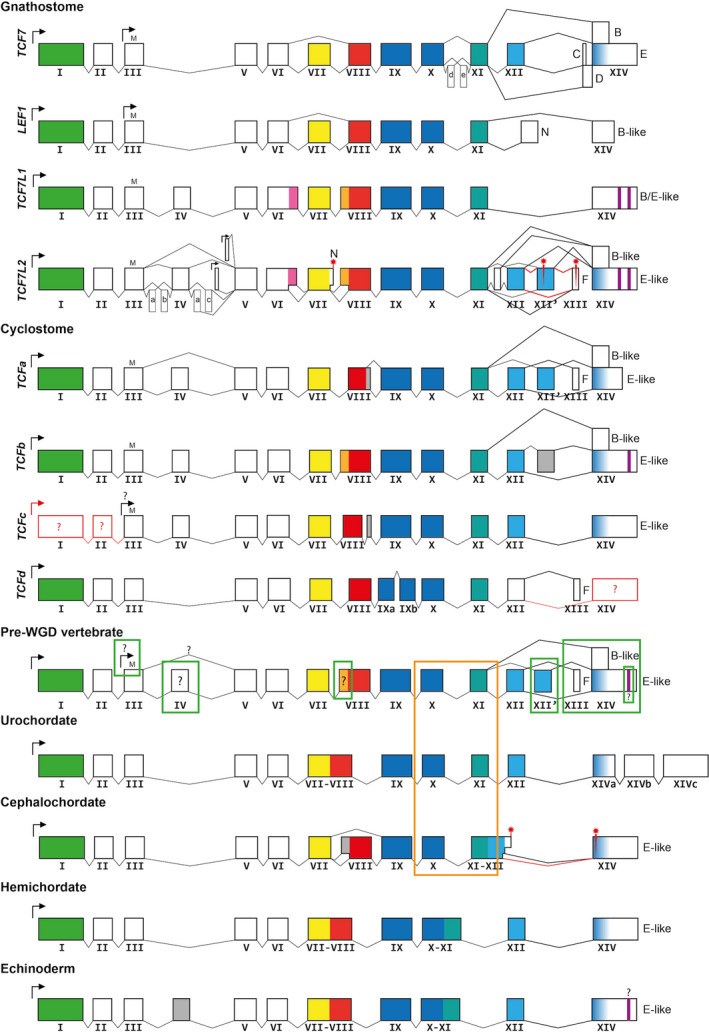
TCF gene models. Composite gene models of the species included in this study (specified in Appendix S1). Boxes represent exons, and lines represent splicing. Arrows represent transcription start sites. Widely conserved exons are numbered with Roman numerals; lower‐case letters indicate species‐specific exons (a, conserved within mammals; b, conserved within birds and reptiles; c, conserved within teleosts; d, conserved within teleosts except *Danio rerio*; e, conserved within reptiles). “M” represents the conserved methionine found on exon III. Red asterisks represent early stop codons. Question mark indicates lack of unambiguous experimental evidence. Following previous consensus, C‐terminal isoforms are named with capital letters (B, C, D, E, F, N). The color codes of the exons are as follows: green, β‐catenin binding domain; pink, LVPQ motif; orange, SxxSS motif; yellow, co‐repressor helper domain; red = co‐repressor domain; dark blue, High Mobility Group box; turquoise, nuclear localization signal; light blue, C‐clamp domain; purple, CtBP motif; gray, exon with no wider sequence conservation. Green squares highlight vertebrate innovations, and orange square highlights a chordate innovation. WGD, whole‐genome duplications

Gene structure comparisons among deuterostomes revealed highly conserved exon–intron structures in general, particularly among vertebrates. We used this widely conserved gene structure to unify the exon numbering among TCF/LEF genes (Figure 3). Although the overall vertebrate gene structure is also remarkably conserved among the single TCF genes of invertebrate genomes, some lineage‐specific peculiarities can be identified.

Within gnathostomes, our comprehensive analysis establishes conservation of diagnostic domains and motifs for the paralogous TCF/LEF ohnolog subfamilies, which is much more widely and more deeply conserved throughout the clade than previously expected.

*TCF7* genes (also known by the synonym *Tcf1*) exhibit three main characteristic features. First, *TCF7* has an alternative translation start site on exon III that produces a protein without the BCBD, previously named the ∆N isoform (Van de Wetering et al., 1996). Secondly, exon VII, which encodes the helper of the co‐repressor domain, can be alternatively spliced. Last, alternative splicing can produce four different C‐termini that differ significantly in the C‐clamp domain (Roël et al., 2003). The C‐clamp domain is composed of two motifs: the CRARF motif encoded in exon XII and the RKKCIRY motif encoded near the beginning of exon XIV. Isoform E is the only one containing a complete C‐clamp domain, and isoform C is characterized by a disrupted C‐clamp (CRARF motif only), while isoforms B and D lack any C‐clamp sequence (Van de Wetering et al., 1996). Specifically, isoforms C and E result from the splicing of exon XII to the first and second alternative acceptor sites on exon XIV, respectively, while isoforms B and D derive from exon XI spliced directly to the second and first alternative acceptor sites on exon XIV, respectively. The alternative splicing of exon XII and the usage of different splicing acceptor sites on exon XIV cause both the absence of a CRARF domain and the change of the reading frame in the last exon. The D isoform has not always been recognized in previous literature; however, it is clearly annotated in the genome sequences of *Homo sapiens*, *G*. *gallus*, and *D*. *rerio*, and similar sequences in the genomes of *Latimeria chalumnae* and *Lepisosteus oculatus* lead us to include it here.

**FIGURE 4:**
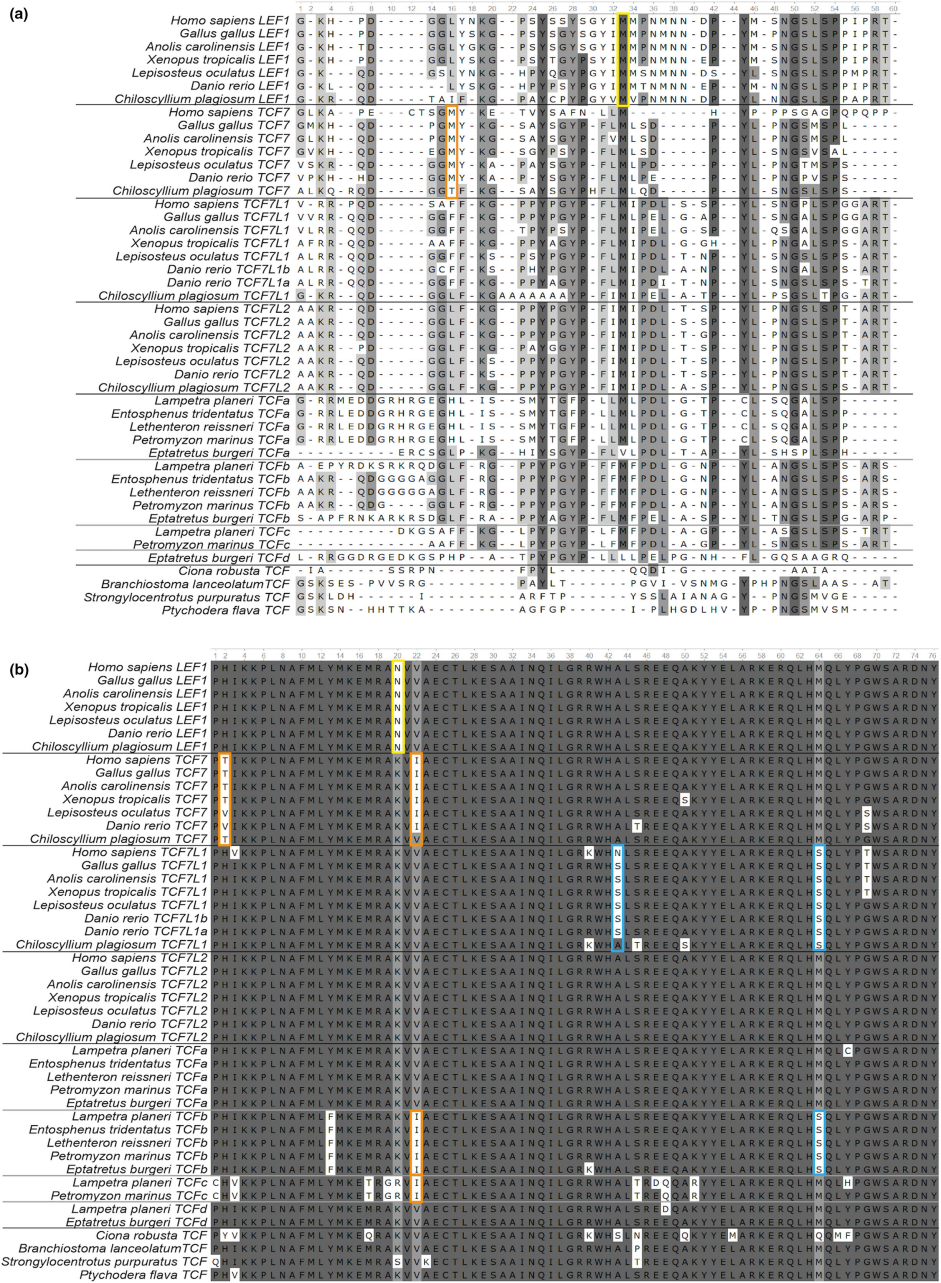
Amino‐acid sequence alignment of parts of TCF/LEF proteins. Intensity of gray coloration represents degree of conservation: the higher the intensity, the greater the conservation. (a) TCF/LEF exon III alignment. Orange and yellow squares highlight *TCF7* and *LEF1* alternative translation start sites, respectively. (b) TCF/LEF HMG‐box alignment. Orange, yellow, and blue squares highlight specific amino‐acid changes for TCF7, LEF1, and TCF7L1, respectively

*LEF1* genes (also known as *Tcf1α*) share some *TCF7* features such as an alternatively spliced exon VII and also a ∆N isoform that starts in exon III (Hovanes et al., 2001). However, compared with *TCF7*, the translation start site of the LEF1 ∆N isoform is located at a different amino‐acid position (Figure 4a). In addition, *LEF1* encodes two different C‐terminal isoforms, named N and B‐like, although it does not have a C‐clamp domain and hence also differs from *TCF7* in this respect.

*TCF7L1* (also known as *Tcf3*) does not have any alternative splicing reported (Molenaar et al., 1996). Among the four TCF/LEF genes, *TCF7L1* is the only one with no additional transcription start site (to generate a ∆N isoform). Our analysis confirms that *TCF7L1* paralogs encode a single isoform throughout gnathostomes. As with *LEF1*, it does not encode a C‐clamp domain. However, *TCF7L1* encodes additional motifs that are not present in TCF7 or LEF1, but which it shares with TCF7L2. These are the LVPQ and SxxSS motifs, flanking the helper co‐repressor domain (exon VII), and two CtBP motifs in exon XIV. In addition, *TCF7L1* possesses an additional exon compared with *TCF7* and *LEF1*, here named exon IV (Figure 3), which it shares with *TCF7L2*. Although it has been described that the coding sequence on exon IV is suitable for phosphorylation (Hikasa et al., 2010), its implications for TCF7L1 transcriptional activity remains unexplored.

*TCF7L2* (also known as *Tcf4*) stands out for its abundance of isoforms from alternative promoter use, extensive alternative splicing, and an increased number of exons (Duval et al., 2000). *TCF7L2* has a similar basic exon composition to *TCF7L1*, including exon IV and LVPQ, SxxSS, and CtBP motifs, but similar to *TCF7* it includes a C‐clamp domain and ∆N isoforms. Specifically, TCF7L2 has at least two different ∆N isoforms, encoded by novel exons located between exon IV and exon V. Interestingly, exon VII and the sequences encoding the LVPQ and SxxSS motifs can be alternatively spliced in *TCF7L2*. Also, an isoform with the BCBD but without the HMG box and NLS is described, previously called isoform N, that occurs when the splicing donor site on exon VII is skipped and there is read‐through to a stop codon (Kennell et al., 2003; Figure 3). Finally, *TCF7L2* possesses one extra exon between exon XI and exon XII, two XII exons (here denoted exon XII and exon XII′) with two distinct C‐clamp motifs of CRARF (exon XII) and CRALF (exon XII′), and one extra exon between exon XII′ and exon XIV (i.e., exon XIII, which leads to the F isoforms). This increase in the number of exons and alternative splicing results in 14 different C‐terminal isoforms, 4 of them with C‐clamp domains (2 with CRARF and 2 with CRALF).

In addition to this general overview, we have also identified some species‐specific exons in *TCF7* and *TCF7L2* previously described for some of the species (e.g., *H. sapiens* exon “a” and *D*. *rerio* exon “c” in Figure 3; Young et al., 2019) and here seen conserved only inside their respective clades (Figure 3; Appendix S1). However, the exon–intron structures of the four ohnolog subfamilies (*TCF7*, *LEF1*, *TCF7L1*, and *TCF7L2*) are revealed as being very highly conserved across all gnathostomes examined, with *LEF1* paralogs showing similarities with *TCF7*, and *TCF7L1* clear similarities with *TCF7L2*, yet *TCF7* and *TCF7L2* also sharing aspects of their generally more complex exon–intron structures.

Cyclostome *TCF* genes encode remarkably similar domains to the gnathostome TCF/LEF genes and are organized with a broadly similar exon–intron structure. However, our analysis reveals that cyclostome *TCF* genes do not simply map to the gnathostome TCF/LEF ohnolog subfamily framework, since no particular cyclostome gene contains all of the same features as any specific gnathostome ohnolog (Figure 3).

*TCFa* (*P*. *marinus* chromosome 8) shows some similarity in gene structure with *TCF7L2*: alternative splicing of exon IV, no alternative splicing of exon VII, and the presence of exon XII′, XIII, and B‐like and E‐like isoforms from exon XIV. Nevertheless, *TCFa* does not have the LVPQ, SxxSS, and CtBP motifs, which is more reminiscent of *TCF7*. Additionally, the XII′ exon (encoding for a CRALF motif) has only been found in *E*. *burgeri TCFa*. Thus, *TCFa* possesses features resembling both the gnathostome *TCF7* and *TCF7L2* subfamilies.

*TCFb* (*P. marinus* chromosome 41) seems generally more similar to *TCF7L2* owing to it encoding an exon IV, the SxxSS motif in exon VIII, two exons between exon XI and exon XIV, and one CtBP motif at the 3′ end of exon XIV. However, the exon between XII and XIV does not contain a CRARF or CRALF motif, and alternative splicing of exon IV is not observed in the transcriptomic data.

*TCFc* (*P. marinus* chromosome 68) appears to have some similarities to *TCF7*, such as the lack of LVPQ, SxxSS, and CtBP motifs and possession of a C‐clamp. However, only a single isoform is observed in the transcriptomic data, and nothing equivalent to exon I or exon II is found. Interestingly, the methionine at position 33 in exon III, which is required for the translation start of the gnathostome *LEF1* ∆N isoform, is potentially conserved (see below and Figure 4a). Therefore, it is possible that *TCFc* has its only translation start site in the equivalent of exon III, as in the case of the *LEF1* ∆N isoform.

Finally, *TCFd* (found only in *E*. *burgeri* and *L*. *planeri*) shows some similarity with *TCF7/LEF1*, by lacking an exon IV and LVPQ and SxxSS motifs. However, it also shows similarity with *TCF7L2* since, although exon XIV has not been found in the current data, it includes exon XIII, which is characteristic of the *TCF7L2* subfamily. Additionally, *TCFd* has some oddities: exon III does not contain the conserved methionine at position 33, exon IX is split into two exons, and exon XII does not contain the characteristic CRARF motif.

Invertebrate TCFs conserve the four main domains characteristic of the TCF/LEF family (the BCBD, co‐repressor domain, HMG box with NLS motif, and C‐clamp), thus having a motif composition highly similar to *TCF7*. However, the exon–intron distribution is slightly different in each lineage surveyed here (Figure 3). Urochordates have the helper co‐repressor exon and the co‐repressor domain in a single exon (exon VII–VIII) and three exon XIVs (XIVa, XIVb, and XIVc). Cephalochordates have the NLS and the CRARF exon fused (exon XI–XII) and have alternative donor splicing sites from the “fused” exon XI/XII. Also, uniquely, at least among the species that we surveyed here, cephalochordates show alternative splicing of exon VIII, which encodes the co‐repressor domain. Hemichordates and echinoderms have the helper and co‐repressor domains fused (exon VII–VIII) and the exon encoding the second part of the HMG box fused with the NLS exon, forming a single exon X–XI.

### 3.4 A comparative analysis of TCF/LEF protein‐coding sequences in deuterostomes

In addition to similarities (and differences) between the gross structure of the genes and their encoded proteins, protein sequence comparisons among vertebrate TCF/LEFs reveal further conserved and divergent features (Figure 4; Appendix S2).

Alignment of exon III (cf. Figure 3) protein‐coding sequence reveals conservation of the translation initiation AUG codon (encoding methionine) used in gnathostome LEF1 paralogs to encode the ∆N isoform (Figure 4a). Remarkably, this methionine is conserved not just among LEF1 paralogs, but in all gnathostome TCF/LEF subfamilies, including TCF7L1 paralogs, for which there is no evidence of a ∆N isoform; TCF7 paralogs, for which transcriptome analysis suggests a translation start site of this TCF7 ∆N isoform located at a different amino‐acid position within the same exon (further towards the 5′ end, or N‐terminal, Figure 4a); and TCF7L2 paralogs, for which transcriptome analysis suggests a translation start site in subsequent exons (cf. Figure 3). Furthermore, our analysis of cyclostome exon III reveals further conservation of this methionine, suggesting that perhaps cyclostome TCF genes also have the potential to encode ∆N isoforms (with the exception of *TCFd*). For cyclostome *TCFc*, the current genome sequence data lack sequences for an exon I (encoding BCBD) and an exon II. It is therefore possible that *TCFc* generally uses a transcription start site leading to translation start in exon III, as in the case for the *LEF1* ∆N isoform. Further, more detailed genomic sequence data around the *TCFc* locus may provide better evidence for or against this notion in the future, and additional transcriptomics data may be able to confirm or refute ∆N isoform expression for *TCFc* and the other cyclostome *TCF* genes. On the other hand, while there is some conservation of some exon III sequences among deuterostomes (Figure 4a), this methionine codon and sequences flanking it are clearly not conserved in invertebrates, indicating they do not encode a LEF1‐like ∆N isoform.

Alignment of TCF/LEF HMG‐box sequences also shows high sequence conservation among all deuterostomes examined (Figure 4b), except for a small number of residues, which appear to be specific (or even diagnostic) for particular gnathostome TCF/LEF subfamilies. LEF1 is distinguished by an asparagine (N) at position 20; TCF7 is distinguished by a threonine (T) or valine (V) at position 2 and an isoleucine (I) at position 22; and TCF7L1 is distinguished by an asparagine (N) or serine (S) at position 43, and a serine (S) at position 64. Interestingly, TCF7L2 does not possess any distinctive amino‐acid change relative to the overall TCF/LEF consensus sequence of deuterostomes, and therefore its HMG‐box sequence appears most prototypical and most invertebrate‐like.

We checked if the HMG‐box sequences in cyclostome TCFs had any of these distinctive residues that distinguish the gnathostome TCF subfamilies. Interestingly, lamprey TCF proteins again do not easily map onto a gnathostome ohnolog subfamily framework, since they tend to show mixed TCF/LEF subfamily features in their amino‐acid sequences, just like they did in their exon–intron organization and domain compositions. In the case of TCFa and TCFd, they do not contain any of these “diagnostic” amino‐acids, and their HMG‐box amino‐acid sequences are identical to TCF7L2 and the wider deuterostome consensus, except for *L. planeri* TCFd, which have a divergent amino‐acid substitution at position 47. However, TCFb has two distinctive amino acids: an isoleucine at position 22, characteristic of TCF7, and a serine (S) at position 64, characteristic of TCF7L1. Finally, TCFc contains one of the “diagnostic” amino acids that is also shared with TCFb and is characteristic of the TCF7 subfamily (i.e., isoleucine (I) at position 22), but also has at least eight additional amino‐acid substitutions, making it the most divergent vertebrate TCF analyzed here. However, two of these substitutions are shared with divergent residues in some gnathostome TCF/LEFs: the valine (V) at position 3 is shared with *Homo sapiens* TCF7L1, and the threonine (T) at position 45 is shared with *Danio rerio* TCF7 and *Chiloscyllium plagiosum* TCF7L1.

Invertebrate TCFs contain some amino‐acid substitutions relative to the deuterostome consensus, but none was shared with the “diagnostic” residues in vertebrate TCF/LEF proteins, and are therefore likely invertebrate lineage specific.

### 3.5 Comparative synteny analysis of chordate TCF/LEF genomic neighborhoods

**FIGURE 5:**
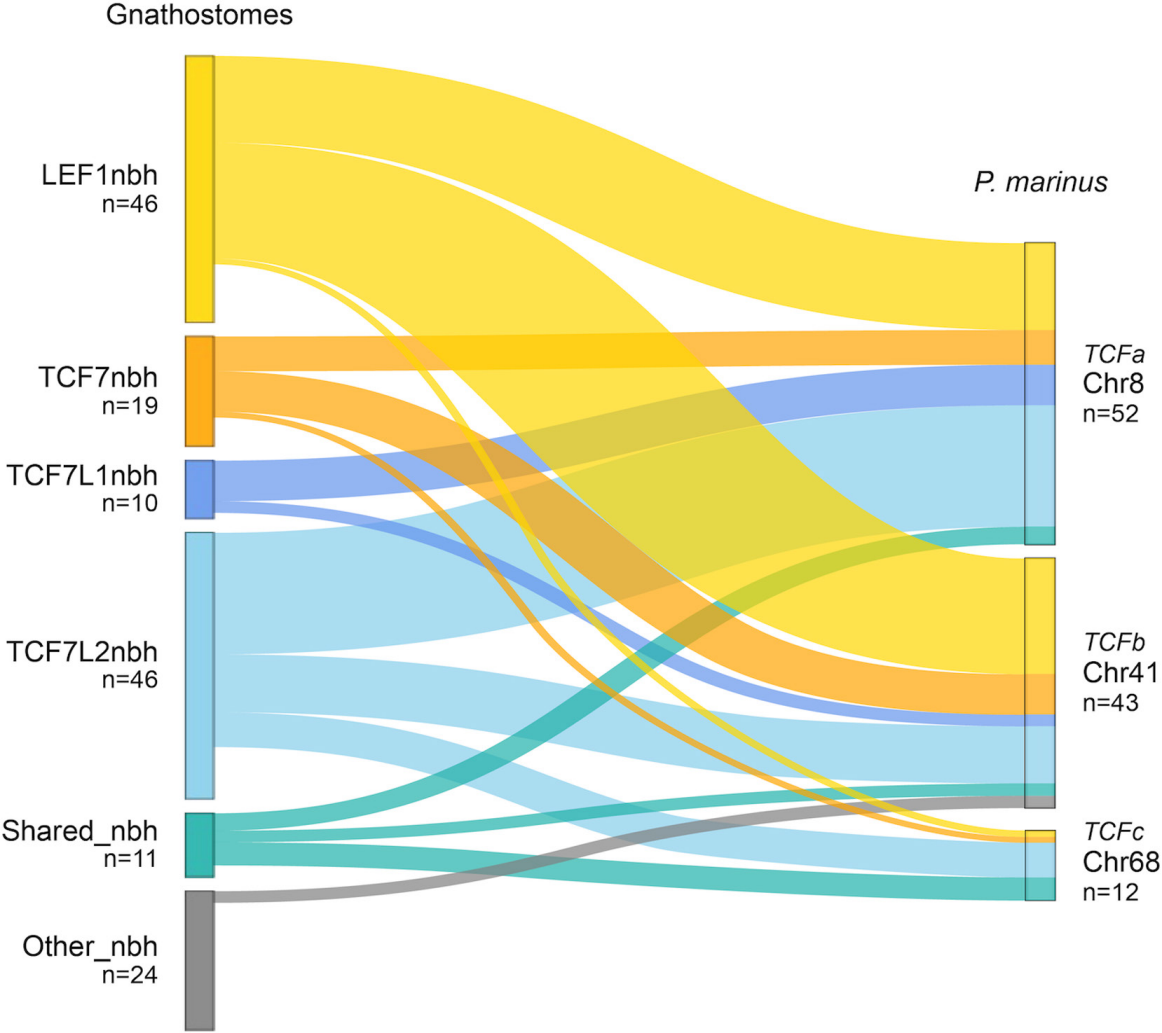
Distribution of CLGQ cyclostome orthologs in gnathostome TCF/LEF neighborhoods and *P*. *marinus TCF*‐containing chromosomes. LEF1nbh, LEF1 neighborhood; TCF7nbh, TCF7 neighborhood; TCF7L1nbh, TCF7L1 neighborhood; TCF7L2nbh, TCF7L2 neighborhood; Shared_nbh, shared neighborhood (ortholog present in more than one gnathostome TCF/LEF neighborhood); Other_nbh, other neighborhood (ortholog not present in any gnathostome TCF/LEF neighborhood); *n* = number of genes

Synteny (i.e., patterns of gene linkage) can provide another source of data to help distinguish evolutionary relationships of genetic loci and the genes within them, independent from the kind of gene sequence analyses described above (i.e., molecular phylogenies and diagnostic residues and comparisons of gene structure and domain composition). Because the cyclostome lineage is the most basal (i.e., early branching) within the vertebrates, we were particularly interested to know to which TCF/LEF subfamily neighborhood each lamprey TCF neighborhood might associate. We based our synteny analyses on the genes that belong to the same ancestral chordate linkage group as the *TCF* gene (i.e., the CLGQ; Simakov et al., 2020), to observe if there has been a segregation of these genes in the gnathostomes that helps to define specific *TCF* subfamily neighborhoods (i.e., *LEF1* neighborhood, *TCF7* neighborhood, *TCF7L1* neighborhood, *TCF7L2* neighborhood in Figure 5), and we compared this segregation with the one observed in lamprey.

We found a total of 156 CLGQ genes in the *P*. *marinus* lamprey genome. A binomial test showed statistically significant support for these genes being distributed across four particular *P. marinus* chromosomes (i.e., chromosomes 8, 41, and 68 that contain the three *TCF* genes of this lamprey species, and chromosome 22) (Table S1 and Figure S3). Of the 156 CLGQ *P. marinus* orthologs, 19 have orthologs in the gnathostome *TCF7* neighborhood, 46 in the *LEF1* neighborhood, 10 in the *TCF7L1* neighborhood, and 46 in the *TCF7L2* neighborhood, with a further 11 having at least two paralogs (ohnologs) shared between neighborhoods, and 24 not being assigned to any gnathostome TCF/LEF neighborhood (Figure 5). A Barnard’s test (Table 2) shows significant association of the *TCF7L1* neighborhood and *TCF7L2* neighborhood genes with the *P*. *marinus TCFa*‐containing chromosome 8 (*p* = 1.07 × 10^−2^ and *p* = 2.68 × 10^−2^, respectively), and significant association of *LEF1* neighborhood genes with the TCFb‐containing chromosome 41 (*p* = 3.46 × 10^−3^). There was no significant association of CLGQ genes observed for *P*. *marinus TCFc*‐containing chromosome 68.

**TABLE 2:** Barnard's test contingency tables of TCF/LEF neighborhood distribution in *Petromyzon marinus* TCF‐containing chromosomes

	Chr8	No‐Chr8	*p*	Chr41	No‐Chr41	*p*	Chr68	No‐Chr68	*p*
TCF7nbh	6	13	ns	7	12	ns	1	18	ns
noTCF7nbh	46	91		36	101		11	126	
LEF1nbh	15	31	ns	20	26	3.46 × 10^−3^	1	45	ns
noLEF1nbh	37	73		23	87		11	99	
TCF7L1nbh	7	3	1.07 × 10^−2^	2	8	ns	0	10	ns
noTCF7L1nbh	45	101		41	105		12	134	
TCF7L2nbh	21	25	2.68 × 10^−2^	10	36	ns	6	40	ns
noTCF7L2nbh	31	79		33	77		6	104	

Abbreviations: nbh, neighborhood; ns, nonsignificant; *p*, Barnard’s test *p*‐value.

Our synteny analysis therefore suggests affinity of the cyclostome *TCFa* neighborhood with those of gnathostome *TCF7L1* and *TCF7L2* genes, and of the cyclostome *TCFb* neighborhood with those of the gnathostome *LEF1* genes, while the cyclostome *TCFc* neighborhood shows such high levels of genomic rearrangement that synteny comparisons cannot reveal any affinity to gnathostome TCF/LEF gene loci.

## 4 DISCUSSION

Based on limited information, it was previously assumed that four paralogs (ohnologs) (*TCF7*, *LEF1*, *TCF7L1*, and *TCF7L2*) evolved in vertebrates from a single invertebrate *TCF* gene through two rounds of whole‐genome duplications (2R WGD), with *TCF7* ohnologs appearing to represent the most prototypical (i.e., most invertebrate *TCF*‐like vertebrate ohnolog) and *TCF7L2* ohnologs presumed to be the most derived via evolving most vertebrate‐specific innovations subsequent to these WGD‐associated TCF/LEF gene duplications (Hoppler & Waterman, 2014).

Our comprehensive analysis of deuterostome sequence information now strongly supports an evolutionary emergence via 2R WGD of a widely conserved four‐ohnolog standard from a single pre‐vertebrate *TCF* gene. Importantly, our analysis of sequence information from jawless vertebrates (cyclostomes), together with more detailed comparison between the four jawed vertebrate (gnathostome) ohnolog subfamilies, now casts doubt on the idea that *TCF7* represents the most prototypical ohnolog, despite its invertebrate‐like appearance in terms of exon–intron structure and domain composition. Instead, our analyses lead us to suggest that many specific innovations evolved in a mostly *TCF7L2*‐like single ancestral *TCF* gene in the vertebrate ancestor before the whole‐genome duplications at the base of the vertebrates.

This novel perspective on vertebrate TCF/LEF evolution provides an essential foundation for future experiments aimed at improving our understanding of the roles of Wnt/β‐catenin signaling in vertebrate evolution, and of the conserved and divergent features of the four vertebrate TCF/LEF ohnolog subfamilies for embryonic development and disease.

### 4.1 Four conserved TCF/LEF ohnologs evolved via 2R WGD in jawed vertebrates

In this study, we have identified that the majority of gnathostomes possess one copy of each of the four conserved TCF/LEF subfamilies (*TCF7*, *LEF1*, *TCF7L1*, and *TCF7L2*, sometimes known by the synonyms *TCF1*, *TCF1*α, *TCF3*, and *TCF4*, respectively). This finding clearly validates the use of model species to study the functions of this conserved set of TCF/LEF genes in vertebrate and human embryonic development and beyond. Future research should also focus on studying conservation of expression of paralogs in different species to help robustly infer the functional roles that are widely conserved versus those that are more restricted or even lineage specific.

Our phylogenetic analysis also provides greater detail to more robustly support the hypothesis that the four gnathostome TCF/LEF subfamilies originated from the 2R WGD that occurred early in vertebrate evolution (Aase‐Remedios & Ferrier, 2021; Holland & Ocampo Daza, 2018). The topology of the phylogeny is consistent with the 1R WGD producing the two ohnologs that were the ancestors of the *TCF7/LEF1* genes on the one hand and the *TCF7L1/TCF7L2* genes on the other, followed by the second of the 2R WGD then producing the four gnathostome TCF/LEF ohnolog subfamilies: *TCF7*, *LEF1*, *TCF7L1*, and *TCF7L2* (Figure S2). This sequence of events is also supported by the gene structure of *LEF1* being more similar to the one of *TCF7*, and the gene structure of *TCF7L1* being more similar to the one of *TCF7L2*; though both *LEF1* and *TCF7L1* have a much less complex gene structure than their closest paralogs, *TCF7* and *TCF7L2*, respectively.

### 4.2 Evolution of TCF/LEF paralogs in jawless vertebrates

The increasing number of sequence resources from cyclostome species indicate that it is timely to examine the TCF/LEF gene complements of these basal (i.e., early branching) vertebrate lineages, since they have the potential to provide insight into the transition from the sequence and organization of the single‐copy *TCF* of invertebrates to the multi‐paralog state of the gnathostome vertebrates. Cyclostomes possess multiple *TCF* genes. We identified three in *P*. *marinus*, four in *L*. *planeri*, and two in *E*. *tridentatus*, *L*. *reissneri*, and *E*. *burgeri*. Taking into account the lamprey phylogeny (Potter et al., 2015), and the fact that three *L*. *planeri* and *P. marinus* paralogs appear to be orthologous to each other (Figure S1), this result suggests a gene loss of *TCFc* in the other cyclostomes. This hypothesized gene loss correlates with a highly rearranged genomic neighborhood around *P*. *marinus TCFc* relative to other lamprey species, and with this level of rearrangement not being observed around the gene loci encoding the other two *TCF* paralogs. It seems reasonable to speculate that this apparently elevated level of genomic rearrangement of the *TCFc* loci in lampreys has somehow led to the loss of the gene in some lamprey species. It is not possible to do the same synteny analysis for *TCFd* owing to the lack of a reference genome in *L*. *planeri* and relatively short scaffolds in the currently available *E*. *burgeri* assembly. However, the presence of *TCFd* in *E*. *burgeri* and *L*. *planeri* implies, as well, a possible gene loss of *TCFd* in the other cyclostome species. It will be particularly interesting to identify gene neighborhoods around these *TCFd* genes to see if there is any conserved synteny that might indicate paralogy with the *P. marinus* chromosome 22, given the concentration of CLGQ orthologs on this *P. marinus* chromosome even though it does not contain a *Tcf* gene itself (Table S1 and Figure S3). This then would add further support to the hypothesis of secondary loss of *TCFd*, at least in the *P. marinus* lineage. However, it could be that our inability to find certain genes in the cyclostome genome sequences is due to gaps in the respective assemblies, which may be resolved with further future sequencing data.

It would be interesting in future work to examine the expression and function of these *TCF* genes across lamprey species to see if, for example, *TCFc* has a particularly restricted and specialized role that might predispose it to loss without serious phenotypic consequences. In addition, the ability to compare genomic and transcriptomic data across several cyclostome species has allowed us to curate and revise the gene models of cyclostome *TCF* genes, alongside our revisions to the gene models of the better‐known gnathostome genes (Appendix S1). This has been essential for attempting to resolve how the cyclostome paralogs relate to the gnathostome ohnologs. This in turn is important for understanding how these cyclostome and gnathostome genes can be robustly compared at the expression and function levels in future work, and also how the evolutionary diversification of these major effectors of canonical Wnt signaling might have contributed to vertebrate evolution.

Our phylogenetic analysis reveals that the cyclostome TCF proteins probably group together in a single clade (maximum‐likelihood bootstrap support of 54%; Figure 2) that is potentially located within the gnathostome TCF7L1/L2 clade, although the bootstrap values that support this location are very low (maximum‐likelihood bootstrap of 36%; Figure 2), so effectively the cyclostome clade position can be represented as a polytomy. Thus, the phylogeny tentatively implies that the cyclostome genes duplicated independently from the gnathostome genes, at least to some extent, separately from the 2R WGD events. Also, the ancestral vertebrate (i.e., before divergence into cyclostome and gnathostome lineages) may have possessed a TCF sequence that was most similar to a TCF7L2‐like protein judging from the very short branch lengths of the TCF7L2 clade, leading to the branch termini (and hence protein sequences) being closest to the ancestral vertebrate node.

However, the relationship of the cyclostome *TCFs* to the 2R WGD, and whether the lamprey genes arose via the same 2R as gnathostomes or instead from at least some independent duplications (as some propose to have occurred in cyclostome evolution; Simakov et al., 2020), cannot confidently be determined from our results, which show mixtures of features. Specifically, we found that *TCFa* has a gene structure with similarities to both *TCF7* and *TCF7L2*, but the sequence of the HMG box alone is most similar to that of TCF7L2, and the synteny shows affinity to a mixed *TCF7L1/L2* gene neighborhood. On the other hand, *TCFb* has a *TCF7L2*‐like gene structure, a LEF1/TCF7L1‐like HMG box, and a *LEF1* gene neighborhood. The *TCFc* and *TCFd* genes are difficult to include in these sorts of comparisons because of their presence in only two cyclostome species, potentially partial sequence data, and unknown genomic location of *TCFd* and highly rearranged genomic loci around *TCFc*.

In the case of the synteny data, this mixture of different gnathostome‐like neighborhoods is consistent with something akin to the hypothesis of tetralogy (Aase‐Remedios & Ferrier, 2021; Martin & Holland, 2014), which would imply that the divergence of the cyclostome and gnathostome lineages occurred in a brief time period close to the 2R WGD events, such that there has been differential sorting of paralogs between cyclostomes versus gnathostomes during the process of rediploidization after the polyploidization events associated with the WGD. The mixed features present in the TCF/LEF genes themselves, when comparing cyclostomes versus gnathostomes, could then be accounted for by lineage‐specific ohnolog resolution (LORe) (Robertson et al., 2017), which was described for the salmonid WGD, but more generally describes the way that ohnologs that arise relatively close to the WGD events then experience independent divergence in the sister lineages arising close to the WGD. Such a process could well lead to the mixtures of TCF/LEF features that we see when comparing cyclostomes with gnathostomes.

### 4.3 A gene model for a *TCF7L2*‐like single ancestral pan‐vertebrate *TCF* gene

Taken together, these results lead us to propose a pre‐WGD vertebrate *TCF* gene model (Figure 3). Before our investigation, it had been assumed (e.g., Hoppler & Waterman, 2014) that *TCF7* represents the most prototypical vertebrate *TCF* ohnolog. However, our detailed comparison of the amino‐acid sequences in the all‐important HMG box and particularly our investigation of *TCF* gene sequences in cyclostomes, instead, highlight the very deep ancestry within the vertebrate lineage of many of the features that had previously been assumed to be subsequent innovations mostly restricted to the *TCF7L2* ohnolog in gnathostomes.

The mixture of characteristics and their affinities in the cyclostome *TCF* genes relative to those of gnathostomes is consistent with their distribution during evolution via tetralogy and LORe (Aase‐Remedios & Ferrier, 2021; Martin & Holland, 2014; Robertson et al., 2017). Consequently, it seems reasonable to conclude that the ancestral pre‐WGD *TCF* gene of vertebrates (i.e., gnathostomes plus cyclostomes) would have been a composite of features found across the gnathostome *TCF7*, *LEF*, *TCF7L1*, and *TCF7L2* ohnolog subfamilies as well as the cyclostome genes. The rationale for inferring the components of our pre‐WGD gene model (Figure 3) is thus as follows. First, the presence of the CRARF‐encoding exon XII is clear, due to its widespread presence across *TCF* ohnologs but its loss from the gnathostome *LEF* and *TCF7L1* subfamilies. Second, the presence of both B‐ and E‐like C‐termini, without or with a C‐clamp respectively, is also highly likely to be present due to its widespread presence across vertebrate ohnologs, particularly in *TCF7* members of the *TCF7/LEF* gnathostome clade, the *TCF7L2* members of the *TCF7L1/L2* clade, and multiple cyclostome genes. Third, the presence of exon XII′, exon XIII, and at least one CtBP motif in the C‐terminus of the ancestral E‐like isoform is included in our model because such features are found in the *TCF7L2* family as well as in the cyclostome *TCFa*, *TCFd*, and *TCFb* genes, respectively (though see further discussion below). Fourth, inclusion of an exon IV is also likely due to presence in gnathostome *TCF7L1* and *L2* genes as well as cyclostome genes. However, this presupposes that the cyclostome genes indicate ancestral features of the vertebrates as a whole, rather than only some of the post‐1R ohnolog characteristics (again, discussed further below). Whether this ancestral exon IV was alternatively spliced or not remains an open question. Fifth, the SxxSS may well have been present given that it is found in both the gnathostome *TCF7L1* and *L2* subfamilies as well as the cyclostome *TCFb* genes. A question mark over this inference (Figure 3) is due to a possible alternative scenario in which this motif evolved after the 1R WGD (see below). Additionally, due to the sequence conservation observed in the acceptor‐splice‐site region of exon VIII, it is possible that this motif was already alternatively spliced in the pre‐WGD vertebrate TCF. However, the absence of transcript evidence for use of this acceptor splice site in the sequence of *TCFb* genes does not support this ancestral splicing, and therefore it is not included in the pre‐WGD gene model. The LVPQ motif is not included in our pre‐WGD model, since it is only found in the gnathostome *TCF7L1* and *L2* subfamilies so that its evolutionary origin post‐1R in the ancestral ohnolog to these two subfamilies is a distinct possibility. This would, in turn, imply that this motif was either lost from the cyclostome genes, if they are also members of this post‐1R ancestral group, or the cyclostome genes diverged before the origin of the LVPQ motif in the ancestral precursor to the two gnathostome subfamilies. Sixth, a methionine residue is encoded by a widely conserved position in exon III that corresponds to where the ∆N isoform of LEF1 starts, as well as possibly cyclostome TCFc. In addition to the conservation of this methionine, the production of ∆N isoforms that use it as the translational start may also have been ancestral, but, if so, this capability has been lost from the *TCF7L1* subfamily and moved to alternative start methionines in TCF7 and TCF7L2. There is no evidence that cyclostome TCFa and b use an alternative start methionine, but further transcriptome data may help to resolve this possibility.

There is an alternative scenario to the cyclostome genes indicating many features of the pre‐WGD ancestral gene, which introduces some question marks over a couple of the components of this ancestral pre‐WGD gene model. Since the current consensus is that cyclostomes arose after 1R, but prior to 2R (Simakov et al., 2020), then if all of the extant cyclostome *TCF* genes do form a single clade that is within the *TCF7L1‐2* clade, as tentatively implied in our phylogenetic tree, the cyclostome descendant of the *TCF7‐LEF1* clade has been lost. Whether this cyclostome clade really is more closely related to the gnathostome post‐1R *TCF7L1/L2* clade than the *TCF7/LEF* clade is a little ambiguous, due to the lack of significant support values on the node uniting the cyclostome clade with the *TCF7L1* clade as well as lack of universally significant support for the *TCF7L1‐L2* clade itself. Thus, the cyclostome genes, according to the phylogenetic tree, probably arose via independent duplications from those of gnathostomes, but their phylogenetic location relative to the gnathostome clades (and hence the 1R and 2R WGD events) is unclear. Formally, they could be an outgroup to all gnathostome ohnolog subfamily clades (i.e., descendent from the pre‐1R WGD state) or they could be allied to the gnathostome *TCF7L1‐L2* or *TCF7‐LEF* clade (i.e., indicating post‐1R, but pre‐2R), but this would require the loss of the cyclostome equivalent to the ancestral *TCF7‐LEF* or *TCF7L1‐L2* ohnolog ancestral gene, respectively. Finally, the cyclostome genes could be orthologous to the gnathostome *TCF7L1* subfamily (i.e., post‐2R), but this would have required extensive secondary loss of genes from cyclostomes. It is intriguing that the ancestral cyclostome likely had four *TCF* genes (*a*–*d*), which might then also raise the possibility of the cyclostome paralogs being one‐to‐one orthologs with the gnathostome ohnologs. This, however, would require a significant change to the current sequence phylogeny topology, which might happen once complete sequence information is available for all the cyclostome genes, but this seems unlikely given the magnitude of the topology change required. These considerations could then change some of our inferences about the composition of the pre‐WGD ancestral vertebrate *TCF* gene. Thus, whether the pre‐WGD ancestral gene possessed an exon IV (possibly alternatively spliced), LVPQ and SxxSS motifs, and CtBP motifs is less certain (Figure 3). Also, the presence of a methionine encoded by a conserved position in exon III is almost certain, but whether this was used as the translational start site to generate a ∆N isoform ancestrally is currently unresolved.

Notwithstanding any currently remaining ambiguity in the details of the evolution of the cyclostome genes (above), the comparisons among the vertebrate genes as a whole relative to the invertebrate orthologs permit us to propose a tentative TCF/LEF evolutionary scenario, as follows:

- The pre‐1R vertebrate ancestor possessed one single *TCF* gene, which our analysis suggests already contained features of the eventual gnathostome *TCF7L2* ohnolog subfamily, including alternative splicing at the 3′ tail and possibly the central region (encoded by exon IV), and alternative 5′ transcription and translation start sites on exon III (mimicking a TCF7/LEF1 feature). The SxxSS motif may also have originated at this stage of the evolutionary scenario.
- After the 1R WGD, this pan‐vertebrate ancestral gene duplicated into two ohnologs:
  1. a *TCF7L1‐L2* ancestral gene, which maintained many features of this pre‐1R WGD *TCF*, though it probably additionally acquired the LVPQ motif, and was therefore in many ways even more similar to the eventual *TCF7L2* ohnolog subfamily; and
  2. a *TCF7‐LEF1* ancestral gene, that in many ways was already similar to the *TCF7* ohnolog subfamily, probably having lost exon IV, XII′, and XIII and SxxSS and CtBP motifs (if these were indeed present ancestrally) and having acquired alternative splicing of exon VII.
- After the 2R WGD, gnathostomes have:
  1. the *TCF7L2* ohnolog subfamily, derived from the *TCF7L1‐L2* ancestor with acquisition of additional ∆N isoforms, alternative splicing of LVPQ and SxxSS motifs, and acquisition of other *TCF7L2*‐specific exons;
  2. the *TCF7L1* ohnolog subfamily, derived from the (mainly *TCF7L2*‐like) common *TCF7L1‐L2* ancestor by losing any alternative start sites and alternative splicing, and therefore only encoding one isoform, which is always full length (i.e., no ∆N isoforms), always encodes the SxxSS and LVPQ motifs, and, importantly, by losing exon XII, XII′, and XIII encodes no CRARF sequence and therefore no C‐clamp domain or alternative C‐termini;
  3. the *TCF7* ohnolog subfamily, derived from the (already *TCF7*‐like) common *TCF7‐LEF1* ancestor by acquisition of a new translation start site on exon III and an additional alternative splicing at the 3′ tail, leading to C and D isoforms; and,
  4. the *LEF1* ohnolog subfamily, derived from the same (mainly *TCF7*‐like) common *TCF7‐LEF1* ancestor by losing exon XII (CRARF motif) and therefore any E‐like isoform, but retaining the possible ancestral translation start site for encoding a ∆N isoform and gaining the exon encoding the N isoform.

### 4.4 Evolutionary innovations in vertebrate TCF/LEF genes

By comparing the gene structure of invertebrates and the pre‐1R WGD vertebrate, we can identify one chordate innovation and six vertebrate innovations (Figure 3). All invertebrates outside the chordate clade have one exon encoding homologous sequences represented as exon X and XI in our model (see Figure 3 for deuterostomes), which implies that the acquisition of the intron that separates these two exons is a chordate innovation. The potential vertebrate innovations are: (1) acquisition of a transcription start site on exon III (∆N isoform); (2) acquisition of exon IV; (3) acquisition of the SxxSS motif; (4) evolving C‐clamp‐lacking TCF proteins (B‐like and F isoforms) by acquisition of alternative splicing and exon XIII; (5) duplication of exon XII (giving exon XII′) and acquiring CRALF motifs; and (6) acquisition of the CtBP motif.

All these structures have been found to modify the transcriptional activity of TCF/LEF proteins. This, together with the fact that they had been acquired during the early evolution of vertebrates, suggests that they may well be key features of vertebrate‐specific Wnt signaling and the associated aspects of vertebrate evolution, development, and physiology. Specifically, exon IV encodes protein sequence that has been found to be subject to phosphorylation in TCF7L1 (Hikasa et al., 2010), which results in less repressor activity. Additionally, the absence of exon IV in *TCF7L1* transcripts has been linked to high risk of hepatic cancer (Tomimaru et al., 2013), while exon IV absence in *TCF7L2* is associated with higher risk of type 2 diabetes (Pradas‐Juni et al., 2014). In addition, the LVPQ, SxxSS, and CtBP motifs have been found to increase the repressor activity of TCF7L1 and TCF7L2 (Brannon et al., 1999; Liu et al., 2005; Valenta et al., 2003). Thus, these vertebrate innovations could have been key to provision of another level of regulation of TCF/LEF transcriptional activity during vertebrate evolution. It is perhaps also noteworthy that the CtBP motif described in vertebrate TCFs (PLSLxxK) is different from the one observed in echinoderms (PxDLSxK), which has instead been described in different vertebrate proteins that also interact with CtBP (Chinnadurai, 2002). Additionally, in the invertebrate fruit fly *Drosophila melanogaster*, it has been described that CtBP is needed along with pangolin (the *Drosophila TCF* ortholog) for regulation of Wnt‐target genes, despite pangolin not having any CtBP motif in its protein coding sequence (Fang et al., 2006). This raises the possibility that CtBP is part of a Wnt‐target gene‐regulating protein complex containing TCF and that direct interactions of CtBP and TCF have evolved repeatedly in, for example, echinoderms and vertebrates via independent evolution of CtBP‐interacting motifs in the respective TCF proteins. In addition, the C‐clamp domain has been described to be required for strong DNA‐binding affinity of TCF/LEF proteins to some DNA sequences (Atcha et al., 2007; Hoverter et al., 2012; Weise et al., 2010). Therefore, the loss of the C‐clamp domain from the B‐like isoform will modify its DNA‐binding capability, suggesting the possible evolution of new vertebrate‐specific Wnt target genes.

Finally, we have described five different amino‐acid changes in the TCF/LEF HMG‐box domain that are distinctive for specific TCF/LEF subfamilies. The HMG box is the domain responsible for the binding to and recognition of Wnt response elements (WREs), so, changes in the coding sequences of the HMG box could alter this recognition as well as binding affinities. Recently, one study identified several mutations in the TCF7L2 HMG box that are associated with lung cancer (Su et al., 2020). One of these mutations is precisely the change from valine (V) to isoleucine (I) at HMG‐box position 22 that we have found to be a specific change conserved across the TCF7 subfamily (Figure 4b). This suggests that the described subfamily‐specific amino‐acid changes may have an important role in TCF/LEF subfunctionalization and/or neofunctionalization. Hence, it will be interesting to study if the described TCF/LEF subfamily‐specific amino‐acid changes can modify the affinity of TCF/LEF proteins to WREs and, as a consequence, the binding to target gene promoters and enhancers.

In conclusion, our study provides the foundation for a better understanding of the role of Wnt/β‐catenin signaling in vertebrate development and evolution by supplying a clear and comprehensive overview of the structures of TCF/LEF genes and their encoded proteins, highlighting motifs and sequences that warrant future functional analysis.

However, there are some limitations to the current analyses. While our analysis of *TCF* genes in cyclostomes has been essential for our understanding of early pan‐vertebrate TCF gene evolution and the ancestry of ohnologs in gnathostomes, the precise evolutionary pathway to the cyclostome TCF/LEF genes remains rather obscure. However, this may nevertheless be indicative of the phenomena impacting paralog evolution among diverging lineages that are associated with large‐scale duplications such as WGD, such as tetralogy, rediploidization, and the possibilities for independent duplications, particularly given the 400 million years or so of independent evolution of cyclostomes and gnathostomes.

Despite this uncertainty surrounding the cyclostome genes, we can still infer the ancestral gene structure and sequence for the pre‐1R WGD vertebrate ancestral *TCF* and detect likely chordate and vertebrate innovations in this gene family, which will be fruitful avenues for further research on function to discover how these innovations may have impacted chordate and vertebrate evolution and the diversification of the roles of this major effector of canonical Wnt/β‐catenin signaling in vertebrate biology and human diseases.

## Supporting information

Supplementary Material

Appendix S1

Appendix S2
