# Supplementary material for: Evolutionary diversification of the canonical Wnt signaling effector TCF/LEF in chordates
Source: Dev Growth Differ. 2022 Feb 3;64(3):120–37. doi: 10.1111/dgd.12771 (PMC9303524; doi:10.1111/dgd.12771)
Supplement: Supplementary file 1 — Supplementary Material [file DGD-64-120-s003.pdf]

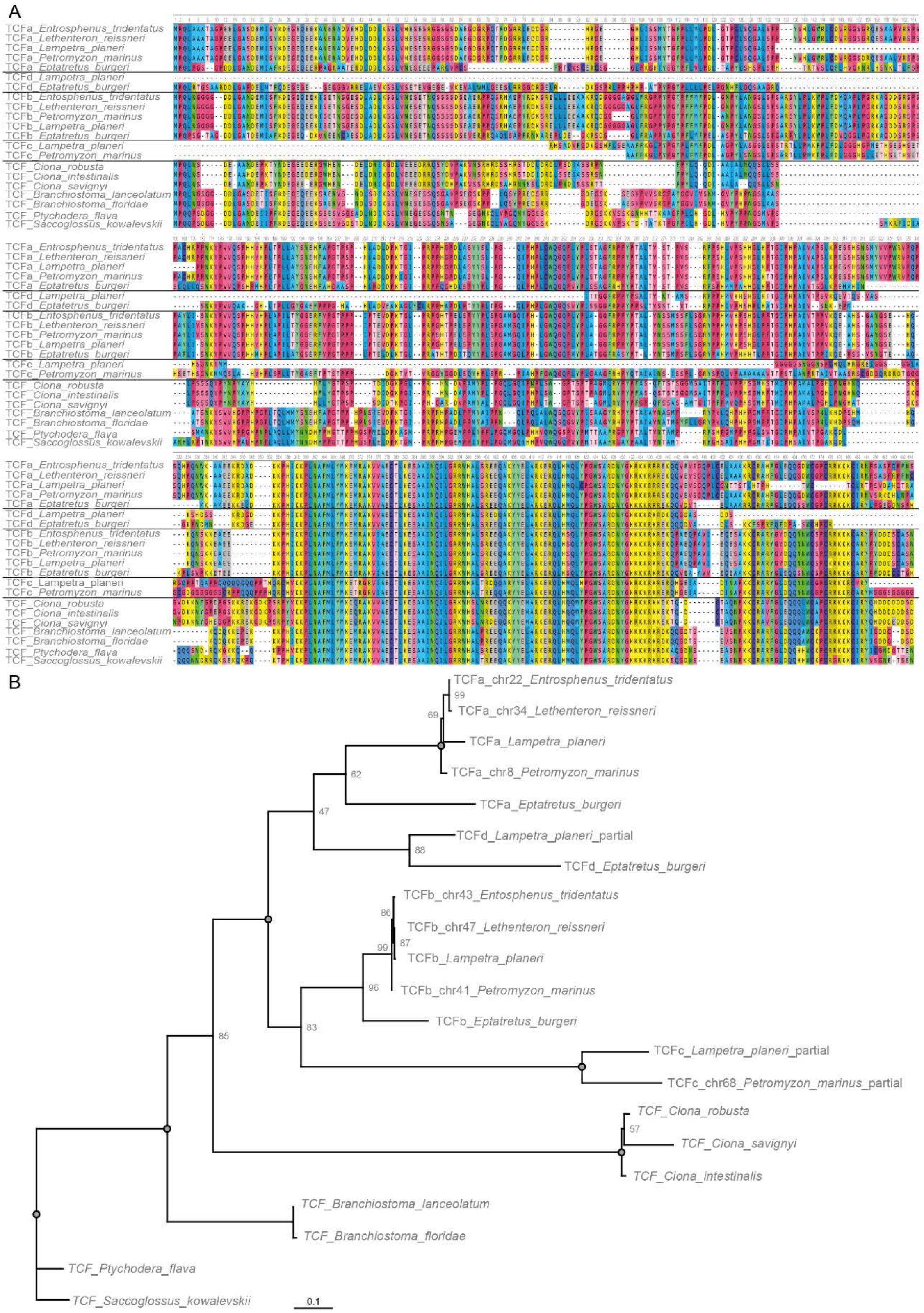

Supplemental Figure 1. **Alignment and phylogeny of cyclostome TCFs**. A) Alignment of Cyclostome TCF amino-acid sequences, including the partial sequences of *L. planeri* TCFc and TCFd, and *P. marinus* TCFc. B) Maximum-likelihood tree using the sequence alignment from A. Node values are the percentage bootstrap support values. The circles in the node indicates a support value of 100. The tree is rooted with non-chordate sequences as the outgroup.

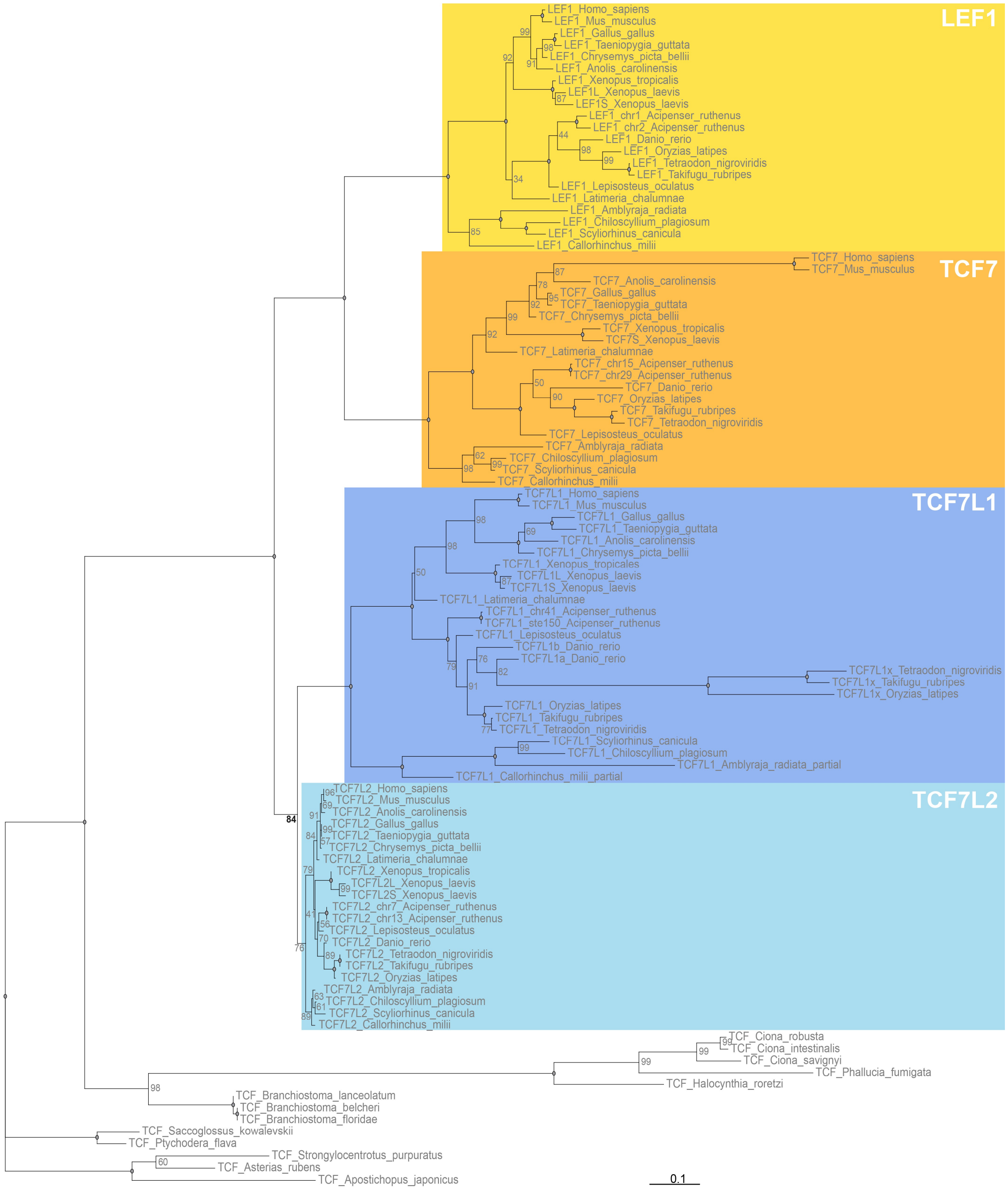

Supplemental Figure 2. Gnathostome TCF/LEF phylogeny. Maximum-likelihood tree using the sequence alignment from Supplemental File 2 excluding the cyclostome sequences. Node values are the percentage bootstrap support values. The circles in the node indicate a support value of 100. Tree is rooted with non-chordate sequences as the outgroup.

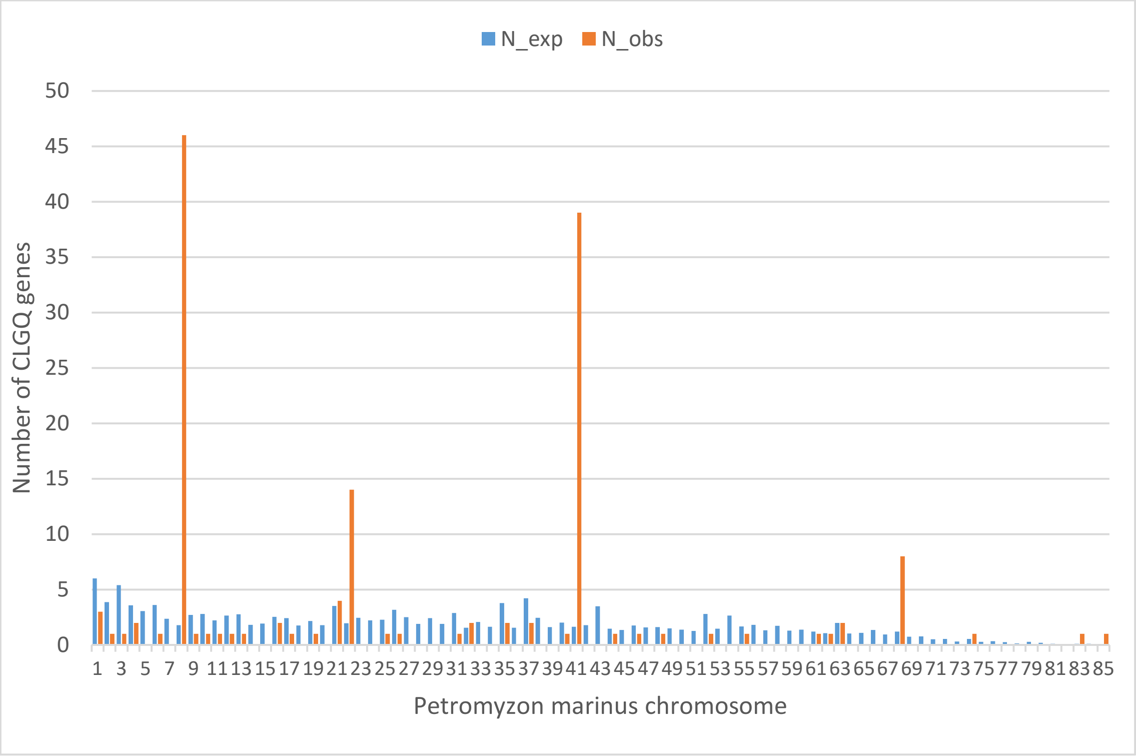

Supplemental Figure 3. Distribution of CLGQ genes across *P. marinus* chromosomes. N_exp: number of CLGQ genes expected. N_obs: Number of CLGQ genes observed.

Supplemental Table. Distribution of CLGQ genes across *P. marinus* chromosomes and its Binomial p-value. N_genes: total number of genes described in the chromosome. P_CLGQ: probability of having a CLGQ gene. N_exp: Number of CLGQ genes expected according to the probability calculated. N_obs: real number of CLGQ observed. P_value: P-value of the Binomial Test.

Chromosome	N_genes	P_CLGQ	N_exp	N_obs	P_value
1	756	0.0385	6	3	0.942
2	487	0.0248	4	1	0.980
3	681	0.0347	5	1	0.996
4	450	0.0229	4	2	0.875
5	387	0.0197	3	0	1
6	454	0.0231	4	1	0.974
7	299	0.0152	2	0	1
**8**	**224**	**0.0114**	**2**	**46**	**1.03E-50**
9	342	0.0174	3	1	0.935
10	352	0.0179	3	1	0.940
11	278	0.0142	2	1	0.893
12	335	0.0171	3	1	0.932
13	349	0.0178	3	1	0.939
14	230	0.0117	2	0	1
15	245	0.0125	2	0	1
16	321	0.0164	3	2	0.727
17	306	0.0156	2	1	0.914
18	220	0.0112	2	0	1
19	273	0.0139	2	1	0.887
20	225	0.0115	2	0	1
21	443	0.0226	4	4	0.470
**22**	**246**	**0.0125**	**2**	**14**	**1.37E-08**
23	309	0.0157	2	0	1
24	278	0.0142	2	0	1
25	286	0.0146	2	1	0.899
26	398	0.0203	3	1	0.959
27	315	0.0160	3	0	1
28	240	0.0122	2	0	1
29	306	0.0156	2	0	1
30	238	0.0121	2	0	1
31	362	0.0184	3	1	0.945
32	195	0.0099	2	2	0.458
33	261	0.0133	2	0	1
34	206	0.0105	2	0	1
35	475	0.0242	4	2	0.893
36	196	0.0100	2	0	1
37	532	0.0271	4	2	0.926
38	309	0.0157	2	0	1
39	204	0.0104	2	0	1
40	253	0.0129	2	1	0.868
**41**	**206**	**0.0105**	**2**	**39**	**1.86E-41**

42	227	0.0116	2	0	1
43	441	0.0225	4	0	1
44	186	0.0095	1	1	0.774
45	172	0.0088	1	0	1
46	221	0.0113	2	1	0.830
47	200	0.0102	2	0	1
48	203	0.0103	2	1	0.801
49	190	0.0097	2	0	1
50	174	0.0089	1	0	1
51	159	0.0081	1	0	1
52	354	0.0180	3	1	0.941
53	186	0.0095	1	0	1
54	335	0.0171	3	0	1
55	211	0.0108	2	1	0.816
56	228	0.0116	2	0	1
57	168	0.0086	1	0	1
58	217	0.0111	2	0	1
59	164	0.0084	1	0	1
60	173	0.0088	1	0	1
61	154	0.0078	1	1	0.705
62	136	0.0069	1	1	0.660
63	252	0.0128	2	2	0.595
64	129	0.0066	1	0	1
65	139	0.0071	1	0	1
66	169	0.0086	1	0	1
67	121	0.0062	1	0	1
**68**	**151**	**0.0077**	**1**	**8**	**3.26E-05**
69	95	0.0048	1	0	1
70	99	0.0050	1	0	1
71	64	0.0033	1	0	1
72	70	0.0036	1	0	1
73	38	0.0019	0	0	1
74	70	0.0036	1	1	0.430
75	36	0.0018	0	0	1
76	45	0.0023	0	0	1
77	31	0.0016	0	0	1
78	18	0.0009	0	0	1
79	37	0.0019	0	0	1
80	26	0.0013	0	0	1
81	15	0.0008	0	0	1
82	10	0.0005	0	0	1
83	16	0.0008	0	1	0.117
84	15	0.0008	0	0	1
85	10	0.0005	0	1	0.075
