# Supplementary material for: Evolutionary diversification of the canonical Wnt signaling effector TCF/LEF in chordates
Source: Dev Growth Differ. 2022 Feb 3;64(3):120–37. doi: 10.1111/dgd.12771 (PMC9303524; doi:10.1111/dgd.12771)
Supplement: Supplementary file 3 — Appendix S2 [file DGD-64-120-s002.pdf]

```
CLUSTAL W 2.0 multiple sequence alignment

LEF1_Homo_sapiens_V2                    MPQLSGGGGGGGDPELCATDEMIPFKDEGDPQKEKIFAEISHPEEEGDLADIKSSLVNESEI-IP-AS-- 70
LEF1_Mus_musculus_V2                    MPQLS-GGGGGGDPELCATDEMIPFKDEGDPQKEKIFAEISHPEEEGDLADIKSSLVNESEI-IP-AS-- 70
LEF1_Gallus_gallus_V2                   MPQLPG-GGGGGDPELCATDEMIPFKDEGDPQKEKIFAEISHPEEEGDLADIKSSLVNESEI-AP-GS-- 70
LEF1_Taeniopygia_guttata_V2             MPQLPG-GGGGGDPELCATDEMIPFKDEGDPQKEKIFAEISHPEEEGDLADIKSSLVNESEI-AP-GA-- 70
LEF1_Anolis_carolinensis_V2             MPQLSGGGGGGGDPELCATDEMIPFKDEGDPQKEKIFAEISHPEEEGDLADIKSSLVNESET-IS-SS-- 70
LEF1_Chrysemys_picta_bellii_V2          MPQLPGGGGGGGDPELCATDEMIPFKDEGDPQKEKIFAEISHPEEEGDLADIKSSLVNESEI-TP-NS-- 70
LEF1_Xenopus_tropicalis_V2              MPQLSGGNGGGGDPELCATDEMIPFKDEGDPQKEKIYAEISNPEEEGDLADIKSSLVNETEI-IP-SS-- 70
LEF1L_Xenopus_laevis_V2                 MPQLSGGNGGGGDPELCATDEMIPFKDEGDPQKEKIYAEISNPEEEGDLADIKSSLVNETEI-IP-SS-- 70
LEF1S_Xenopus_laevis_V2                 MPQLSGGNGVGGDPELCATDEMIPFKDEGDPQKEKIYAEISNPEEEGDLADIKSSLVNETEI-IP-SS-- 70
LEF1_chr1_Acipenser_ruthenus_V2         MPQLAGGGGGGGDPELCANDEMIPFKDEGDPQKEKIFAEISHSEEEGDLADIKSSLVNESEI-NP-NS-- 70
LEF1_chr2_Acipenser_ruthenus_V2         MPQLAGGGGGGGDPELCANDEMIPFKDEGDPQKEKIFAEISHSEEEGDLADIKSSLVNESEI-NP-N--- 70
LEF1_Latimeria_chalumnae_V2             MPQLSGGGGGGGDPELCANDEMIPFKDEGDPQKEKIFAEISHPEEEGDLADIKSSLVNESEI-NP-ST-- 70
LEF1_Lepisosteus_oculatus_V2            MPQLAGGGGGGGDPELCANDEMIPFKDEGDPQKEKIFAEISHPEEEGDLADIKSSLVNETEI-NP-NS-- 70
LEF1_Danio_rerio_V2                     MPQLS-GGGGGGDPELCATDEMIPFKDEGDPHKEQIFAEISHSEEEGDLAEIKSSLVNETEI-SP-NS-- 70
LEF1_Oryzias_latipes_V2                 MPQLS--GGGGGDPELCATDEMIPFKDEGDPHKEQIFAELSHSEEEGDLADIKSSLVNESES-SP-NS-- 70
LEF1_Tetraodon_nigroviridis_V2          MPQLS---GGGGDPELCATDEMIPFKDEGDPHKEQIFAELSHSEEEGDLADIKSSLVNESES-SP-NS-- 70
LEF1_Takifugu_rubripes_V2               MPQLS---GGGGDPELCATDEMIPFKDEGDPHKEQIFAELSHSEEEGDLADIKSSLVNESES-SP-NS-- 70
Amblyraja_radiata_LEF1                  MPQLN----GGGDPELGANDEMISFKDEGD-QEEKILAEISGSEER-DLADLKSSLVNESEI-SPCNS-- 70
Chiloscyllium_plagiosum_LEF1            MPQLN----SGGDPELGANDEMISFKDEGE-QDDKILAEISATEER-DLADLKSSLVNETET-NASNP-- 70
Scanicula_LEF1                          MPQLN----GGGDPELGANDEMISFKDEGE-QEDKILAEISATEER-DLADLKSSLVNESEI-NPSNS-- 70
Elphany_shark_partial_LEF1              MPQMT---GGGGDPELGANDEMISFKDE-E-QEEKVLAEISATEER-DLADLKSSLVNESEI-NPSNT-- 70
TCF7_Homo_sapiens_V3                    MPQLDSGGAGGGD-DLGAPDELLAFQDEGEEQDDKSRDSAAGPE-R-DLAELKSSLVNESEG-AAGGAGI 70
TCF7_Mus_musculus_V3                    MPQLDSGGAGAGD-DLGAPDELLAFQDEGEEQDDKNRDSAVGPE-R-DLAELKSSLVNESEG-AAAGAGV 70
TCF7_Gallus_gallus_V3                   MPQLGS---GGGD-DLGATDEMLAFKDEGE-QEEKIP-ENAFTE-R-DLADLKSSLVNESEG-S-GSP-A 70
TCF7_Taeniopygia_guttata_V3             MPQLGS---GGGD-DLGATDEMLAFKDEGE-QEEKIP-ENAFTE-R-DLADLKSSLVNESEG-S-GSPAA 70
TCF7_Anolis_carolinensis_V3             MPQLNS---GVGD-DLGANDEMLAFKDEGE-QEEKSQ-ASVFPEGG-DLADLKSSLVNESEG-APGASAA 70
TCF7_Chrysemys_picta_bellii_V3          MPQLSS---GGGD-DLGANDEMIAFKDEGE-QEEKIQ-ENAFTE-R-DLADLKSSLVNESES-N-SSPAA 70
TCF7_Xenopus_tropicalis_V3              MPQMNS---AGED-DLGASDEMISFKDEGD-QEEKIR-ESAFTE-R-DLADLKSSLVNESEV-S-SHPRV 70
TCF7S_Xenopus_laevis_V3                 MPQMNS---AGED-DLGASDEMISFKDEGD-QEEKIR-ENGFTE-R-DLADLKSSLVNESEV-A-SHPRV 70
TCF7_chr15_Acipenser_ruthenus_V3        MPQLNS---GGGD-DLGANDEMIAFKDEGE-QEEKIQ-ENALTE-R-DLADLKSSLVNESEI-S-QNAG- 70
TCF7_chr29_Acipenser_ruthenus_V3        MPQLNS---GGGD-DLGANDEMIAFKDEGE-QEEKIQ-ENALTE-R-DLADLKSSLVNESEI-S-QNAG- 70
TCF7_Latimeria_chalumnae_V3             MPQLNS---GGGD-DLGANDEMISFKDEGD-QEEKIQ-ENAFTE-R-DLADLKSSLVNESET-N-QNP-N 70
TCF7_Lepisosteus_oculatus_V3            MPQLNS---GGGD-DLGANDEMISFKDEGE-QDEKIQ-ENAFTE-R-DLADLKSSLVNESEI-N-QNPN- 70
TCF7_Danio_rerio_V3                     MPQLNG---GGGD-DLGANDEMIAFKDEGD-HEEKIR-ESAFTE-S-DLADLKSSLVSETEI-S-QSP-- 70
TCF7_Oryzias_latipes_V3                 MPQLSS---GGGD-DLGANDEMISFKDEGE-QEEKIQ-ENAFTE-R-DLADLKSSLVNESEI-S-QSPS- 70
TCF7_Takifugu_rubripes_V3               MPQLSS---GGGD-DLGANDEMIAFKDEGE-QEEKIQ-ENALTE-R-DLADLKSSLVNESET-N-QSPN- 70
TCF7_Tetraodon_nigroviridis_V3          MPQLSS---GGGD-DLGANDEMIAFKDEGE-QEEKIQ-ENALTE-R-DLADLKSSLVNESET-N-QSPN- 70
Amblyraja_radiata_TCF7                  MPQLNS---GGGD-DLGANDEMIAFKDEGE-QEEKAS-ENALTE-T-DLADVKSSLVNESET-G-QNIHP 70
Chiloscyllium_plagiosum_TCF7            MPQLNS---GGGD-DLGANDEMISFKDEGE-QEEKAS-ENALTE-R-DLADVKSSLVSESET-N-QNVNP 70
Scanicula_TCF7                          MPQLNS---GGGD-DLGANDEMISFKDEGE-QEEKIS-ENTLTE-R-DLADVKSSLVNESET-N-QNVNP 70
Elphany_shark_partial_TCF7              MPQLNS---GGGD-DLGANDEMISFKDEGE-QEEKIS-ENALTE-R-DLAEVKSSLVNESET-N-QSSNT 70
TCF7L1_Homo_sapiens                     MPQLGGGAAGGGD-DLGANDELIPFQDEGG-EEQEPSSDSASAQ-R-DLDEVKSSLVNESEN-Q-SSS-- 70
TCF7L1_Mus_musculus                     MPQLGGGATSGGD-DLGANDELIPFQDEGG-EEQEPSSDSASAQ-R-DLDEVKSSLVNESEN-Q-SSS-- 70
TCF7L1_Gallus_gallus                    MPQLEP---AAGD-DLGAPDELIAFQDEGE-EQDKGA-GRGSAH-G-DLDELKSSLVSETEG-R-AAAPG 70
TCF7L1_Taeniopygia_guttata              MPQLEP---TGGD-DLGAPDELIAFQDEGE-EQDKGA-GRGSAH-G-DLDELKSSLVSETEN-R--GGPG 70
TCF7L1_Anolis_carolinensis              MPQLQPGGGGSGD-DLGAPDELIPFQDEGD-EQDKGAGGRGSAH-G-DLDELKSSLVHESES-R-GAGGG 70
TCF7L1_Chrysemys_picta_bellii           MPQL---AAAGGD-DLGAPDELIPFQDEGD-EQDKGA-GRGSAH-G-DLDELKSSLVHESEN-R-RASAS 70
TCF7L1_Xenopus_tropicales               MPQLNS---GGGD-ELGANDELIRFKDEGE-QEEKSP-GEGSAE-G-DLADVKSSLVNESEN-H-SSD-- 70
TCF7L1L_Xenopus_laevis                  MPQLNS---GGGD-ELGANDELIRFKDEGE-QEEKSP-GEGSAE-G-DLADVKSSLVNESEN-H-SSD-- 70
TCF7L1S_Xenopus_laevis                  MPQLNS---GGGD-ELGANDELIRFKDEGE-QEEKSP-GEGSAE-G-DLADVKSSLVNESEN-H-SSD-- 70
TCF7L1_chr41_Acipenser_ruthenus         MPQLNG---GGGD-DLGANDEMIPFKDEGE-QEDKIS-ENVSAE-R-DLDDVKSSLVNESEN-N-SSS-- 70
TCF7L1_ste150_Acipenser_ruthenus        MPQLNG---GGGD-DLGANDEMIPFKDEGE-QEEKIS-ENVSAE-R-DLDDVKSSLVNESEN-N-SSS-- 70
TCF7L1_Latimeria_chalumnae              MPQLNS---GGGD-DLGANDEMISFKDEGE-QEEKIS-ENVSAE-R-DLDEVKSSLVNESEN-N-SSS-- 70
TCF7L1a_Lepisosteus_oculatus            MPQLNG---GGGD-DLGANDEMISFKDEGE-QEEKIS-ENVSAE-R-DLDDVKSSLVNESEN-N-SSS-- 70
TCF7L1b_Danio_rerio                     MPQLNG---GGGD-ELGANDEMISFKDEGE-QEDKIS-ENVSAE-R-DLDDVKSSLVNESEN-N-SSS-- 70
TCF7L1_Oryzias_latipes                  MPQLNG---GGGD-DLGANDEMIPFKDEGE-QEEKIS-ENVSAE-R-DLDDVKSSLVNESEN-N-SSS-- 70
TCF7L1_Takifugu_rubripes                MPQLNG---GGGD-DLGANDEMISFKDEGE-QEEKIS-ENVSAE-R-DLDDVKSSLVNESE-----SS-- 70
TCF7L1_Tetraodon_nigroviridis           MPQLNG---GGGD-DLGANDEMISFKDEGE-QEEKIS-ENVSAE-R-DLDDVKSSLVNESE-----SS-- 70
TCF7L1a_Danio_rerio                     MPQLNG---GGGD-DLGANDELISFKDEGE-QEEKIS-ENVSSE-R-DLDEVKSSLVNESEN-N-SSS-- 70
TCF7L1x_Tetraodon_nigroviridis          MPQLNG---EDGD-DLGANDELIAFKDEGE-HEEK---RNVSAE-R-DLDDVKSSLVNESET-N--SS-- 70
TCF7L1x_Takifugu_rubripes               MPQLNG---EDGD-DLGANDELIAFKDEGE-HEEK---RNVSAE-R-DLDDVKSSLVNESET-N--SS-- 70
TCF7L1x_Oryzias_latipes                 MPRLSR--DGDAD-DLGASDELIAFKDEGE-QEEK---RSTSAE-R-DLDDVKSSLVNESES-N--SG-- 70
Scanicula_TCF7L1                        MPQL-----PGAD-DLGASDEMISFQDEGE-QEGKSP-GSESAE-R-DLAEVKSSLVSETEE-PRSSSS- 70
Amblyraja_radiata_TCF7L1_partial        ---------------------------------------------------------------------- 70
Chiloscyllium_plagiosum_TCF7L1          MPQL-----PGGD-DLGANDELISFKDEGE-QEDKRP-GSVSAD-R-DLADVKSSLVNESEQTS-SSSSG 70
Elphany_shark_partial_TCF7L1            -----------GD-DLGANDEMISFKDEGE-QEGKSP-GGASAD-R-DLAEVKSSLVNETETEN-HSS-- 70
TCF7L2_Homo_sapiens_V3                  MPQLNG---GGGD-DLGANDELISFKDEGE-QEEKSS-ENSSAE-R-DLADVKSSLVNESET-N-QNS-- 70
TCF7L2_Mus_musculus_V3                  MPQLNG---GGGD-DLGANDELISFKDEGE-QEEKNS-ENSSAE-R-DLADVKSSLVNESET-N-QNS-- 70
TCF7L2_Gallus_gallus_V3                 MPQLNG---GGGD-DLGANDELISFKDEGE-QEEKIS-ENSSAE-R-DLADVKSSLVNESET-N-QNS-- 70
TCF7L2_Taeniopygia_guttata_V3           MPQLNG---GGGD-DLGANDELISFKDEGE-QEEKIS-ENSSAE-R-DLADVKSSLVNESET-N-QNS-- 70
TCF7L2_Anolis_carolinensis_V3           MPQLNG---GGGD-DLGANDELISFKDEGE-QEEKIS-ENSSAE-R-DLADVKSSLVNESET-N-QNS-- 70
TCF7L2_Chrysemys_picta_bellii_V3        MPQLNG---GGGD-DLGANDELISFKDEGE-QEEKIS-DNSSAE-R-DLADVKSSLVNESET-N-QNS-- 70
TCF7L2_Xenopus_tropicalis_V3            MPQLNG---GGGD-DLGANDEMISFKDEGE-QEEKIS-EISSAE-R-DLADVKSSLVNESET-P-QNS-- 70
TCF7L2L_Xenopus_laevis_V3               MPQLNG---GGGD-DLGANDEMISFKDEGE-QDEKIC-PISSAE-R-DLADVKSSLVNESET-P--HS-- 70
TCF7L2S_Xenopus_laevis_V3               MPQLNG---GGGD-DLGANDEMISFKDEGE-QDEKIC-PISSAE-R-DLADVKSSLVNESET-P--HS-- 70
TCF7L2_chr7_Acipenser_ruthenus_V3       MPQLNG---GGGD-DLGANDEMISFKDEGE-QEEKIS-ENSSAE-R-DLADVKSSLVNESET-N-QNS-- 70
```

```
TCF7L2_chr13_Acipenser_ruthenus_V3      MPQLNG---GGGD-DLGANDEMISFKDEGE-QEEKIS-ENSSAE-R-DLADVKSSLVNESET-N-QNS-- 70
TCF7L2_Latimeria_chalumnae_V3           MPQLNG---GGGD-DLGANDEMISFKDEGE-QEEKIS-ENSSAE-R-DLADVKSSLVNESET-N-QNS-- 70
TCF7L2_Lepisosteus_oculatus_V3          MPQLNG---GGGD-DLGANDEMISFKDEGE-QEEKIS-DNSSAE-R-DLADVKSSLVNESET-N-QNS-- 70
TCF7L2_Danio_rerio_V3                   MPQLNG---GGGD-DLGANDEMISFKDEGE-QEEKIS-ENSSAE-R-DLADVKSSLVNESET-N-QNS-- 70
TCF7L2_Tetraodon_nigroviridis_V3        MPQLNG---GGGD-DLGANDEMISFKDEGE-QEEKIS-ENSSAE-R-DLADVKSSLVNESET-N-QNS-- 70
TCF7L2_Oryzias_latipes_V3               MPQLNG---GGGD-DLGANDEMISFKDEGE-QEEKIS-ENSSAE-R-DLADVKSSLVNESET-N-QNS-- 70
TCF7L2_Takifugu_rubripes_V3             MPQLNG---GGGD-DLGANDEMISFKDEGE-QEEKIS-ENSSAE-R-DLADVKSSLVNESET-N-QNS-- 70
Amblyraja_radiata_TCF7L2                MPQLNS---GGGD-DLGANDEMISFKDEGE-QEEKIS-ENASAE-R-DLADVKSSLVNESET-N-QNS-- 70
Chiloscyllium_plagiosum_TCF7L2          MPQLNS---GGGD-DLGANDEMISFKDEGE-QEEKIS-ENASAE-R-DLADVKSSL-------------- 70
Scanicula_TCF7L2                        MPQLNS---GGGD-DLGANDEMISFKDEGE-QEEKIS-ENASAE-R-DLADVKSSLVNESET-N-QNS-- 70
Elephant_shark_TCF7L2                   MPQLNS---GGGD-DLGANDEMISFKDEGE-QEEKIS-ENASAE-R-DLADVKSSLVNESET-N-QNS-- 70
Lamprey_TCF_chr41                       MPQLNG---GGGD-DLGANDEMISFKDEGE-QEEKIS-ETNSGE-S-DLADLKSSLVNESET-N-QSS-- 70
Pacific_lamprey_TCF_chr43               MPQLNG---GGGD-DLGANDEMISFKDEGE-QEEKIS-ETNSGE-S-DLADLKSSLVNESET-N-QSS-- 70
Lethenteron_reissneri_chr47_TCF         MPQLNG---GGGD-DLGANDEMISFKDEGE-QEEKIS-ETNSGE-S-DLADLKSSLVNESET-N-QSS-- 70
Lamprey_TCF_chr8                        MPQLAA-ATAGPE-ELGASDEMISYKDEGE-QEEKAN-ENADVE-H-DLDDLKSSLVHESES-R-GGS-- 70
Pacific_lamprey_TCF_chr22               MPQLAA-ATAGPE-ELGASDEMISYKDEGE-QEEKAN-ENADVE-H-DLDDLKSSLVHESES-R-GGS-- 70
Lethenteron_reissneri_chr34_TCF         MPQLAA-ATAGPE-ELGASDEMISYKDEGE-QEEKAN-ENADVE-H-DLDDLKSSLVHESES-R-GGS-- 70
Lamprey_putative_TCF_chr68_partial      ---------------------------------------------------------------------- 70
Ciona_robusta_TCF_v3                    MPQLN------SD-E-AANDEPKTYNDEGE-EDERGWHEND------ELDNIKGDLVEEEDRD--QSYD- 70
Ciona_intestinalis_TCF                  MPQLN------SD-E-AAHDEPKTYNDEGE-EDERGWHEND------ELDNIKGDLVEEEDRD--QSYD- 70
Ciona_savignyi_TSA_based                MPQLN------SD-E-AANDEPKTYNDEGE-E-ERGWHEND------ELDNIKGDLVEEEDRD--QSYD- 70
Phallucia_fumigata_TCF                  MPQLN------SD-E-AANDEPKTYNDEGE--DE-GWQESD------ELDNLKGDLVDEVDRD--PSYD- 70
Halocynthia_roretzi_TCF                 MPQLN------SD-E-AATDEPTTYTEE-E-EEQR-WH-NDA-----DLNDIKGDLVEESDCD--PTN-- 70
B_lanceolatum_TCF                       MPQLNG---SGGD-DLGASDETISFKDEGE-QEEKSA-ENVSN----DLSDIKSSLVNESESS--QSGA- 70
B_belcheri_TCF                          MPQLNG---SGGD-DLGASDETISFKDEGE-QEEKSA-ENVSN----DLSDIKSSLVNESESS--QSGA- 70
B_floridae_TCF                          MPQLNG---SGGD-DLGASDETISFKDEGE-QEEKSA-ENVSN----DLSDIKSSLVNESESS--QSGA- 70
Saccoglossus_kowalevskii_TCF            MPQQPS---DGGD-DLGANDEIIPFKDEGE-QEEKSS-ESVSD---SDLNDIKSSLVNEGESS--QSNS- 70
Ptychodera_flava_TCF_TSA                MPQQPS---DGGD-DLGANDEIIPFKDEGE-QEEKSS-ESVSG---SDLNDIKSSLVNEGESS--QSNT- 70
Strongylocentrotus_purpuratus_TCF       MPQQHS---RGGE-DDGPPDETKTYHTEGE-QEEKAS-ENVRD---SDLNDVKSSLIDEGESVS-QKGS- 70
Asterias_rubens_TCF                     MPQQPS---RSGE-DDGPPDETKTYHTEGE-QEEKIN-ENARE---SDLNDVKSSLVNEGESASSKPNT- 70
Apostichopus_japonicus_TCF              MPQEPS---RGGL-DDAAEEEVKTFRTEGD-GEERVI-ENGQD---SDLNDVKSSLVIEGDPTS-LKGA- 70


LEF1_Homo_sapiens_V2                    ---NGHE-VARQA---QTSQEPYHDKARE-HPDDG-KH--PD-----GGLYNKG--PSYSSYSGYIMMPN 140
LEF1_Mus_musculus_V2                    ---NGHE-VVRQA---PSSQEPYHDKARE-HPDEG-KH--PD-----GGLYNKG--PSYSSYSGYIMMPN 140
LEF1_Gallus_gallus_V2                   ---GGHE-VSRQT---P-AQDSYHDKGRE-HPEEG-KH--PD-----GGLYSKG--PSYSGYSGYIMMPN 140
LEF1_Taeniopygia_guttata_V2             ---NGHE-VSRQT---Q-AQDSYHDKGRE-HPEEG-KH--PD-----NGLYSKG--PSYSGYSGYIMMPN 140
LEF1_Anolis_carolinensis_V2             ---NGHE-VSRQG---Q-PQEIYHDKGRE-HPEEG-KH--TD-----GGLYGKG--PSYSGYSGYIMMPN 140
LEF1_Chrysemys_picta_bellii_V2          ---NGHE-VSRQA---Q-AQESYHDKGRE-HSEEG-KH--PD-----GGLYSKG--PSYSGYSGYIMMPN 140
LEF1_Xenopus_tropicalis_V2              ---NSHE-ISRR------RQDSYHEKSRE-HPEDG-KH--PD-----GGLYSKG--PSYTGYPSYIMMPN 140
LEF1L_Xenopus_laevis_V2                 ---NSHE-ISRR------RLDSYHEKSRE-HPEDG-KH--PD-----GGLYSKG--PSYTGYPSYIMMPN 140
LEF1S_Xenopus_laevis_V2                 ---NNHE-ISRR------RQDSYHENPRE-HPEDG-KH--PD-----GSLYSKC--PSYTGYPSYIMMPN 140
LEF1_chr1_Acipenser_ruthenus_V2         ---NNHE-AAR-----QVLQDSYHEKHRE-HCDDG-KH--QD------SLYNKG--HPYPGYPGYIMMSN 140
LEF1_chr2_Acipenser_ruthenus_V2         ---NNHE-AAR-----RVLQDSYHEKHRE-HCDDG-KH--HD------SLYNKG--HSYPGYPGYIMMSN 140
LEF1_Latimeria_chalumnae_V2             ---ISHE-AARS---QVPQDSY-DKARE-HPDEG-KH--PD-----GVLYNKG--P-YQGYPGYIMMPN 140
LEF1_Lepisosteus_oculatus_V2            ---NSHE-AARQS---QVPQDSYHEKHRE-HSDDG-K---QD-----GSLYNKG--HPYQGYPGYIMMSN 140
LEF1_Danio_rerio_V2                     ---NSHD-AARQS---QITPDSYHEKHRD-HPDDG-KL--QD-------LYSKG--HPYPSYPGYIMMTN 140
LEF1_Oryzias_latipes_V2                 ---NNHD-AARQS---QIPPDSYHEKHRD-LIEDG-KH--AD-------MYGKG--HVYPSYPGYIMM-N 140
LEF1_Tetraodon_nigroviridis_V2          ---NSHD-GVRQS---QI-PDSYHEKHRD-HQEDG-KL--QD-------LYSKG--HPYSGYPSYSMMSN 140
LEF1_Takifugu_rubripes_V2               ---NSHD-GVRQS---QI-PDSYHEKHRD-HQEDG-KL--QD-------LYCKG--HPYSGYPSYSMMSN 140
Amblyraja_radiata_LEF1                  ---RGPE-VGRRL---QVIEDSYRNKIRRPGTEEG-KR--LD-----GSMV-----PGFCAYPSYIMIPN 140
Chiloscyllium_plagiosum_LEF1            ---AERE-VGRRP---QVNEEPFRDKARTSIEEVG-KR--QD-----TAIF-KG--PAYCPYPGYVMVPN 140
Scanicula_LEF1                          ---AEPQ-VGRRT---QVSEEPFRDKVRVSTEEGG-KR--HD-----AAMY-KN--PAYCTYPGYIMVPN 140
Elphany_shark_partial_LEF1              ---ADTE-VIRRS---HVSQDSYQDKVRD-RDEEG-KR--QDA----GVMY-KG--PAY--YSGYIMIPN 140
TCF7_Homo_sapiens_V3                    PARGEAE-ALGRE---HAAQRLFPDKLPE-PLEDGLKA--PE---CTSGMY-KE--TVYSAFN-LLM--- 140
TCF7_Mus_musculus_V3                    PAEGAPE-ALGRE---HTSQRLFPDKLPE-SLEDGLKA--PE---CASGMY-KE--TVYSAFN-LLM--- 140
TCF7_Gallus_gallus_V3                   A-AADPE-AIRRV---QDPQRVYQEKLPD-HMDDGMKH--QD-----PGMY-KG--SAYSGYP-FLMLSD 140
TCF7_Taeniopygia_guttata_V3             A-AADAE-ALRRV---QDPQRVYQEKLPD-HMDDGMKH--QD-----PGMY-KG--SAYSGYP-FLMLSD 140
TCF7_Anolis_carolinensis_V3             P-AAPSE-AIRRL---QDPQRVYAEKLPD-HMEEGLKH--QD-----PGMY-KG--SAYSGYP-FVMLSD 140
TCF7_Chrysemys_picta_bellii_V3          A-AADPE-AIRRV---QDPQRVYQEKLPD-HMEDGKIH--QD-----TGMY-KG--SAYSGYP-FLMLSD 140
TCF7_Xenopus_tropicalis_V3              P-ESHPE-AMRRT---QDAQLVYQDKLTD-HMEDGVKH--QD-----EGMY-KG--SGYPSYP-FLMLSD 140
TCF7S_Xenopus_laevis_V3                 P-ETHPE-AIRRA---QDVQLVYQDKFSA-HMEDGIKH--QD-----EGMY-KG--SGYPGYP-FLMLSD 140
TCF7_chr15_Acipenser_ruthenus_V3        ------A-AIRRAH--AEEERAYTDKARD-HLEDLSKR--QD-----GGMY-KG--PAYSGYP-FLMLPD 140
TCF7_chr29_Acipenser_ruthenus_V3        ------A-AIRRAH--AEEERAYTDKARD-HLEDLSKR--QD-----GGMY-KG--PAYSGYP-FLMLPD 140
TCF7_Latimeria_chalumnae_V3             A-VPDPE-AIRRA---QDGQRVYQEKPRD-HLGDGTKH--QD-----GVMY-KS--PAYSGYP-FLMLSD 140
TCF7_Lepisosteus_oculatus_V3            ------S-AIRRVQ--QDEHRVYTEKHRE-HLEDVSKR--QD-----GGMY-KA--PAYSGYP-FLMLPD 140
TCF7_Danio_rerio_V3                     ------A-VIRRGQ--QDEQRIYSDK-RE-HLDDVPKH--HD-----GGMY-KA--P-YSGYP-FLMLPE 140
TCF7_Oryzias_latipes_V3                 -----AA-AARRGH--QGEQRGFPDKHRE-HLEGVSKQ--QD-----GSMY-KS--AAYPGYP-FLMLPD 140
TCF7_Takifugu_rubripes_V3               -----AA-AIRRAQ--QGEQRGFAEKHRE-HLDGVSKH--QD-----GGIY-KT--PTYPGYP-FLMLPD 140
TCF7_Tetraodon_nigroviridis_V3          -----AA-AIRRAQ--QGEQRSFAEKHRE-LID-VSKH--QD-----GGMY-KT--PAYTGYP-FLMLPD 140
Amblyraja_radiata_TCF7                  A-PPDPE-AIARS---RDGQRIYQDKSKE-HIDDVSKQ-HQD-----GGMF-KG--TTYSGYPHFLMLQD 140
Chiloscyllium_plagiosum_TCF7            --ASDLG-AIRRS---QDNQRIYQDKSRG-HIDDALKQ-RQD-----GGTF-KG--SAYSGYPHFLMLQD 140
Scanicula_TCF7                          --ASDPG-AIRRS---QDNQRIYQDKSRG-HIDDVIKQ-RQD-----GGAF-KG--TAYSGYPHFLMLQD 140
Elphany_shark_partial_TCF7              A-ASDPG-AIRRS---QDSQRLYQDKSRG-HIDDVTKQ-RQD-----GGMF-KG--TTYSGYSHFLMLQD 140
TCF7L1_Homo_sapiens                     ---SDSE-AERRPQP-V--RDTF-QKPRD-YFAEV-RR-PQD-----SAFF-KG--PPYPGYP-FLMIPD 140
TCF7L1_Mus_musculus                     ---SDSE-AERRPQP-A--RDAF-QKPRD-YFAEV-RR-PQD-----GAFF-KG--PAYPGYP-FLMIPD 140
TCF7L1_Gallus_gallus                    ---PDAE-AERPPQP----RESF-QKPRD-ALAEVVRR-QQD-----GGFF-KG--PPYPGYP-FLMLPE 140
TCF7L1_Taeniopygia_guttata              P--PGPE-AERPPPP----RESF-QKPRD-SLAEVVRR-QQD-----GGFF-KG--PPYPGYP-FLMLPE 140
```

```
TCF7L1_Anolis_carolinensis              -SSSESE-AERPPRP----RESF-QKPRDASVAEVLRR-QQD-----GGFF-KG--TPYPSYP-FLMIPE 140
TCF7L1_Chrysemys_picta_bellii           ---SDSE-AERPPQP----RESF-QKPRD-YLAEVVRR-QQD-----GGFF-KG--PPYPGYP-FLMIPE 140
TCF7L1_Xenopus_tropicales               ---SDSE-VERRPPP----RETF-EKPRD-YLSEAFRR-QQD-----AAFF-KG--PPYAGYP-FLMIPD 140
TCF7L1L_Xenopus_laevis                  ---SDSE-VERRPPP----REAF-EKHRD-YLTEALRR-QQD-----AAFF-KG--PPYAGYP-FLMIPD 140
TCF7L1S_Xenopus_laevis                  ---SDSE-VERRPPP----RETF-EKPRD-YLSEAFRR-QQD-----AAFF-KG--PPYAGYP-FLMIPD 140
TCF7L1_chr41_Acipenser_ruthenus         ---SDSE-AERRPQPTRQDLENY-EKARE-YFSEALRR-QQD-----GGFF-KS--PHYPGYP-FLMIPD 140
TCF7L1_ste150_Acipenser_ruthenus        ---SDSE-AERRPQPTRQDLENY-EKARE-YFSEALRR-QQD-----GGFF-KS--PHYPGYP-FLMIPD 140
TCF7L1_Latimeria_chalumnae              ---SDSE-AERRPQP-RH-SETF-EKPRD-YLSEALRRQQQD-----GGFF-KG--PPYPGYP-FLMIPD 140
TCF7L1a_Lepisosteus_oculatus            ---SDSEQAERRPQP-RQDSESY-EKPRD-YFTEALRR-QQD-----GGFF-KS--PSYPGYP-FLMIPD 140
TCF7L1b_Danio_rerio                     ---SDSEQTERRPQP-RADLESY-EKARE-YFTEALRR-QQD-----GCFF-KS--PHYPGYP-FLMIPD 140
TCF7L1_Oryzias_latipes                  ---SDSEHAERRPQT-RPDSESY-EKTRE-YFSEALRR-QQD-----GGFF-KS--PHYPGYP-FLMIPD 140
TCF7L1_Takifugu_rubripes                ---SDSEQAERRPQT-RADSESY-DKARD-YFSEALRR-QQD-----GGFF-KS--PHYPGYP-FLMIPD 140
TCF7L1_Tetraodon_nigroviridis           ---SDSEQAERRPEP-VADSESY-DKARD-YFSEALRR-QQD-----GGFF-KS--PHYPGYP-FLMIPD 140
TCF7L1a_Danio_rerio                     ---SDSEQTDRRPRP-RPDLESY-EKQRE-YFAEALRR-QQD-----GGFF-KG--PPYAGYP-FLMIPD 140
TCF7L1x_Tetraodon_nigroviridis          ---SDSEQGGRRSKA-AVDLER---RAR---N--VFRR-RQD-----GGLF-HP--SPYVGYP-FFMIPD 140
TCF7L1x_Takifugu_rubripes               ---SDSEPGDRRPKP-GVDLER---RARHDED--VFRR-RQD-----GGLF-HP--SPYVGYP-FFMIPD 140
TCF7L1x_Oryzias_latipes                 ---SESE-ADRRPRS-RPDAES---RAGLD-G--ALRR-QQD-----DGLF-QP--PPYLRYP-FFMIPD 140
Scanicula_TCF7L1                        ----ESE-ADRRPQP-RS-SGSFAEKIREYHQE-V-KR--QD-----GGLF-KG-----AAYP-FIMIPD 140
Amblyraja_radiata_TCF7L1_partial        -------QAER---------GSESEKRR-----DRDRAPSRD-----GGLF-SA-----ATFP-LLLIP- 140
Chiloscyllium_plagiosum_TCF7L1          --SPETE-TERRHSP------SFGEKARDYQQHGG-KR--QD-----GGLF-KGAAAAAAAYP-FIMIPE 140
Elphany_shark_partial_TCF7L1            --SSDSE-AERRPQ--R--PERYHEKPLHYLDE-V-KR--QD-----GGLF-QG--TAYPGYP-FIMIPD 140
TCF7L2_Homo_sapiens_V3                  --SSDSE-AERRPPP-R--SESFRDKSRE-SLEEAAKR--QD-----GGLF-KG--PPYPGYP-FIMIPD 140
TCF7L2_Mus_musculus_V3                  --SSDSE-AERRPPP-R--SESFRDKSRE-SLEEAAKR--QD-----GGLF-KG--PPYPGYP-FIMIPD 140
TCF7L2_Gallus_gallus_V3                 --SSDSE-AERRPPP-R--SETFRDKSRE-SLEEAAKR--QD-----GGLF-KG--PPYPGYP-FIMIPD 140
TCF7L2_Taeniopygia_guttata_V3           --SSDSE-AERRPPP-R--SETFRDKSRE-SLEEAAKR--QD-----GGLF-KG--PPYPGYP-FIMIPD 140
TCF7L2_Anolis_carolinensis_V3           --SSDSE-AERRPPP-R--SESFRDKSRE-SLEEAAKR--QD-----GGLF-KG--PPYPGYP-FIMIPD 140
TCF7L2_Chrysemys_picta_bellii_V3        --SSDSE-AERRPPP-R--SETFRDKSRE-SLEEAAKR--QD-----GGLF-KG--PPYPGYP-FIMIPD 140
TCF7L2_Xenopus_tropicalis_V3            --SSDSE-AERRPPP-R--SESFRDKSRD-SLEDAAKR--PD-----GGLF-KG--PAYGGYP-FIMIPD 140
TCF7L2L_Xenopus_laevis_V3               --SSDSE-TERRPPP-R--PESFRDKSRD-SLEDAAKR--PD-----GGLF-KG--PTYGGYP-FIMIPD 140
TCF7L2S_Xenopus_laevis_V3               --SSDSE-AERRPPP-R--SESFRDKSRD-SLEDAAKR--PD-----GGLF-KG--PTYGGYP-FIMIPD 140
TCF7L2_chr7_Acipenser_ruthenus_V3       --SSDSE-AERRPPP-R--SETFRDKSRE-SLEEAAKR--QD-----GGLF-KS--PPYPGYP-FIMIPD 140
TCF7L2_chr13_Acipenser_ruthenus_V3      --SSDSE-AERRPPP-R--SETFRDKSRE-SLEEAAKR--QD-----GGLF-KS--PPYPGYP-FIMIPD 140
TCF7L2_Latimeria_chalumnae_V3           --SSDSE-AERRPPP-R--SETFRDKSRE-SLEEAAKR--QD-----GGLF-KG--PPYPGYP-FIMIPD 140
TCF7L2_Lepisosteus_oculatus_V3          --SSDSE-AERRPPP-R--SETFRDKSRE-SLEEAAKR--QD-----GGLF-KS--PPYPGYP-FIMIPD 140
TCF7L2_Danio_rerio_V3                   --SSDSE-AERRPPP-R--SESFRDKTRE-SLEEAAKR--QD-----GGLF-KS--PPYPGYP-FIMIPD 140
TCF7L2_Tetraodon_nigroviridis_V3        --SSDSE-AERRPPP-R--SETFRDKTRE-SLEEAAKR--QD-----GGLF-KS--PPYPGYP-FIMIPD 140
TCF7L2_Oryzias_latipes_V3               --SSDSE-AERRPPP-R--SETFRDKTRE-SLEEAAKR--QD-----GGLF-KS--PPYPGYP-FIMIPD 140
TCF7L2_Takifugu_rubripes_V3             --SSDSE-AERRPPP-R--SETFRDKTRE-SLEEAAKR--QD-----GGLF-KS--PPYPGYP-FIMIPD 140
Amblyraja_radiata_TCF7L2                --SSDSE-AERRPPP-RH-SETFRDKSRE-SLEE-AKR--QDG----GGLF-KG--PPYPGYP-FIMIPD 140
Chiloscyllium_plagiosum_TCF7L2          --------AERRPPP-RQ-PESFRDKSRE-SLEEAAKR--QD-----GGLF-KG--PPYPGYP-FIMIPD 140
Scanicula_TCF7L2                        --SSDSE-AERRPPP-RQ-PETFRDKSRE-SLEEAAKR--QD-----GGLF-KG--PPYPGYP-FIMIPD 140
Elephant_shark_TCF7L2                   --SSDSE-AERRPPP-RH-SESFRDKSRE-SLEEATKR--QD-----GGLF-KG--PPYPGYP-FIMIPD 140
Lamprey_TCF_chr41                       --SSDSE-AERPPQS-RH-AEPYRDKSRELLLEEAAKR--QDG----GGLF-RG--PPYPGYP-FFMFPD 140
Pacific_lamprey_TCF_chr43               --SSDSE-AERPPQS-RH-AEPYRDKSRELLLEEAAKR--QDGGGGAGGLF-RG--PPYPGYP-FFMFPD 140
Lethenteron_reissneri_chr47_TCF         --SSDSE-AERPPQS-RH-AEPYRDKSRELLLEEAAKR--QDGGGGGAGLF-RG--PPYPGYP-FFMFPD 140
Lamprey_TCF_chr8                        --GSDAE-GDGRPQT-------F----------DG-RRLEDDGRHRGEGHL-IS--SMYTGFP-LLMLPD 140
Pacific_lamprey_TCF_chr22               --GSDAE-GDGRPQT-------F----------DG-RRLEDDGRHRGEGHL-IS--SMYTGFP-LLMLPD 140
Lethenteron_reissneri_chr34_TCF         --GSDAE-GDGRPQT-------F----------DG-RRLEDDGRHRGEGHL-IS--SMYTGFP-LLMLPD 140
Lamprey_putative_TCF_chr68_partial      -----------------------------------------------AAFF-KG--LPYPGYP-LFMFPD 140
Ciona_robusta_TCF_v3                    ---VPAKNSR-RDSSHRSTD-DLD---RD-LPSD-IA------SSRPN--------FPYL-------QQD 140
Ciona_intestinalis_TCF                  ---VPAKNSR-RDSSHRSTD-DID---RD-LSSE-IA------SSRSN--------FPYL-------QQD 140
Ciona_savignyi_TSA_based                ---HPAKSSRGRDSAHRNNE-SLD---RD-LPND-IS------SSRTT--------FPYL-------QQD 140
Phallucia_fumigata_TCF                  -SQVPYHYVPYPLLYGASSPSDLD---GK-SG---MT--------RP--------------------PHD 140
Halocynthia_roretzi_TCF                 ---I-NKHSHG----EATSKDVLD---RD-SRDE-IK-----GPGRT-----------YLY------PHD 140
B_lanceolatum_TCF                       -PSEGSK----PLQSYP-REDS-----RR--GDEGSKSES-PVVSRG---------PAYLT------PGV 140
B_belcheri_TCF                          -PSEGSK----PLQSYP-REDS-----RR--GDEGSKAES-PVVSRG---------PAYLT------PGV 140
B_floridae_TCF                          -PSEGSK----PLQSYP-REDS-----RR--GDEGSKAES-PVVSRG---------PAYLT------PGV 140
Saccoglossus_kowalevskii_TCF            -ASEGNK----QLVAGQNYGGSSKDRSKKVE---GSKTD--TAT-KT---------PGFGS------I-P 140
Ptychodera_flava_TCF_TSA                -NSEGNK----QLVPGQNYGGSSKDRSKKVA---GSKSN--HHTTKA---------AGFGP------I-P 140
Strongylocentrotus_purpuratus_TCF       -SSQRNK----RLS-SG-YGDHSKEDHKK-VSD-GSKLDH------I---------ARFTP------YSS 140
Asterias_rubens_TCF                     -TSQRNK----RLGPSE-YVDSSKDAVKKPVGE-GSKAEH-HD---I---------ARFAA------YSN 140
Apostichopus_japonicus_TCF              -NSQRNK----RLNGGE-TYEPPKDANKKNLHE-GEKVDY----------------PRFPP------FTN 140


LEF1_Homo_sapiens_V2                    MNN-DP--YM-SNGSLSPPIPRT----------------------------------SNKVPVVQPSHAV 210
LEF1_Mus_musculus_V2                    MNS-DP--YM-SNGSLSPPIPRT----------------------------------SNKVPVVQPSHAV 210
LEF1_Gallus_gallus_V2                   MNN-DP--YM-PNGSLSPPIPRT----------------------------------SNKVPVVQPSHAV 210
LEF1_Taeniopygia_guttata_V2             MNN-DP--YM-PNGSLSPPIPRT----------------------------------SNKVPVVQPSHAV 210
LEF1_Anolis_carolinensis_V2             MNN-DP--YM-SNGSLSPPIPRT----------------------------------SNKVPVVQPSHAV 210
LEF1_Chrysemys_picta_bellii_V2          MNN-DP--YM-SNGSLSPPIPRT----------------------------------SNKVPVVQPSHAV 210
LEF1_Xenopus_tropicalis_V2              MNN-EP--YM-SNGSLSPPIPRT----------------------------------SNKVPVVQPSHAI 210
LEF1L_Xenopus_laevis_V2                 MNN-EP--YM-SNGSLSPPIPRT----------------------------------SNKVPVVQPSHAV 210
LEF1S_Xenopus_laevis_V2                 MNN-EP--YM-SNGSLSPPIPRT----------------------------------SNKVPVVQPSHAV 210
LEF1_chr1_Acipenser_ruthenus_V2         MNN-DS--YM-PNGALSPSMVRT----------------------------------SNKVPVVQPSHAV 210
LEF1_chr2_Acipenser_ruthenus_V2         MNN-DS--YM-PNDSLSPPMART----------------------------------SNKVPVVQPSHAV 210
LEF1_Latimeria_chalumnae_V2             M-N-DP--YM-SNGSLSPPIART----------------------------------S--YPSIHPSIQK 210
LEF1_Lepisosteus_oculatus_V2            MNN-DS--YL-SNGSLSPPMPRT----------------------------------SNKVPVVQPSHAV 210
LEF1_Danio_rerio_V2                     MNN-EP--YM-NNGSLSPPIPRT----------------------------------SNKVPVVQPSHAV 210
```

```
LEF1_Oryzias_latipes_V2                 MNS-DS--YM-NNGSLSPPMPRT----------------------------------SNKVPVVQPSHAV 210
LEF1_Tetraodon_nigroviridis_V2          MND-SS--YM-NNGSLSPPMPRT----------------------------------SNKVPVVQPSHAV 210
LEF1_Takifugu_rubripes_V2               M-D-AS--YM-NNGSLSPPMPRT----------------------------------SNKVPVVQPSHAV 210
Amblyraja_radiata_LEF1                  LSN-EP--YL-NNGSLSPPGVRT----------------------------------SNKVPVVQPCHAV 210
Chiloscyllium_plagiosum_LEF1            MNN-DP--YL-NNGSLSPPAPRT----------------------------------SNKVPVVQPCHAV 210
Scanicula_LEF1                          LNN-DP--YL-NNGSLSPPVPRT----------------------------------SNKVPVVQPCHAV 210
Elphany_shark_partial_LEF1              MNN-DP--YL-TNGSLSPPMPRS----------------------------------SNKVPVVQPSHAI 210
TCF7_Homo_sapiens_V3                    -----H--YP-PPSGAGPQPQPPL---------------------------------H-KAN--QPPHGV 210
TCF7_Mus_musculus_V3                    -----P--YP-PASGAGPQPQPPL---------------------------------HNKPG--QPPHGV 210
TCF7_Gallus_gallus_V3                   -----P--YL-PNGSMSPL--------------------------------------SNKVPVVQPSHGV 210
TCF7_Taeniopygia_guttata_V3             -----P--YL-PNGSMSPL--------------------------------------SNKVPVVQPSHGV 210
TCF7_Anolis_carolinensis_V3             -----P--YL-PNGSMSPL--------------------------------------SNKVPVGQPSHGV 210
TCF7_Chrysemys_picta_bellii_V3          -----P--YL-PNGSMSPL--------------------------------------SNKVPVVQPSHGV 210
TCF7_Xenopus_tropicalis_V3              -----P--YL-SNGSVSAL--------------------------------------SSKVPVVQPSHGV 210
TCF7S_Xenopus_laevis_V3                 -----P--YL-SNGSVSAL--------------------------------------SNKVPVVQPSHGV 210
TCF7_chr15_Acipenser_ruthenus_V3        -----P--YL-QNGPMSPS--------------------------------------SNKVSVVQQSHGM 210
TCF7_chr29_Acipenser_ruthenus_V3        -----P--YL-QNGPMSPS--------------------------------------SNKVSVVQQSHGM 210
TCF7_Latimeria_chalumnae_V3             -----P--YL-PNGSMSPL--------------------------------------SNKVPVVQPSHGV 210
TCF7_Lepisosteus_oculatus_V3            -----P--YL-PNGTMSPS--------------------------------------SNKVSVVQQPHGV 210
TCF7_Danio_rerio_V3                     -----P--YL-PNGPVSPS--------------------------------------SNKVSVVQQ--GM 210
TCF7_Oryzias_latipes_V3                 -----P--YL-PNSSVSPS--------------------------------------SNKVSVVQQSHGM 210
TCF7_Takifugu_rubripes_V3               -----P--YL-PNGSVSPS--------------------------------------SNKVSVVQQSHGM 210
TCF7_Tetraodon_nigroviridis_V3          -----P--YL-PNGSVSPS--------------------------------------SNKVSVVQQSHGM 210
Amblyraja_radiata_TCF7                  -----P--YL-ANGSLSPT--------------------------------------SNKVPVVQPTHAV 210
Chiloscyllium_plagiosum_TCF7            -----P--YL-SNGSLSPS--------------------------------------SNKVPVVQPSHAV 210
Scanicula_TCF7                          -----P--YL-PNGSLSPS--------------------------------------SNKVPVVQPSHAV 210
Elphany_shark_partial_TCF7              -----P--YL-HNGSLTPS--------------------------------------SNKVPVVQPSHAV 210
TCF7L1_Homo_sapiens                     L-S-SP--YL-SNGPLSPGGARTYLQMKWPLLDVPSSA-----TVKDTRSPSPAHL-SNKVPVVQHPHHM 210
TCF7L1_Mus_musculus                     L-S-SP--YL-SNGPLSPGGARTYLQMKWPLLDVPSSA-----TVKDTRSPSPAHL-SNKVPVVQHPHHM 210
TCF7L1_Gallus_gallus                    L-G-AP--YL-SNGALSPGGARTYLQMKWPLLDVPAGA-----TLKDSRSPSPAHL-SNKVPVVQHAPHM 210
TCF7L1_Taeniopygia_guttata              L-G-SP--YL-ANGALSPGGARTYLQMKWPLLDVPAGA-----TLKDSRSPSPAHL-SNKVPVVQHAHHM 210
TCF7L1_Anolis_carolinensis              L-G-SP--YL-QSGALSPGGARTYLQVKWPLLDVPAGA-----TLKDTRSPSPAHL-SNKVPVVQHAHHM 210
TCF7L1_Chrysemys_picta_bellii           L-G-SP--YL-PNGALSPGAARTYLQMKWPLLDVPAGA-----TLKDSRSPSPAHL-SNKVPVVQHAHHM 210
TCF7L1_Xenopus_tropicales               L-G-GH--YL-PNGALSPS-ARTYLQMKWPLLDSPSTA-----GLKDARSPSPAHL-SNKVPVVQHPHHM 210
TCF7L1L_Xenopus_laevis                  L-G-GH--YL-PNGALSPS-ARTYLQMKWPLLDSPSTA-----GLKDARSPSPAHL-SNKVPVVQHPHHM 210
TCF7L1S_Xenopus_laevis                  L-G-GH--YL-PNGALSPS-ARAYLQMKWPLLDSPSTA-----GLKDARSPSPAHL-SNKVPVVQHPHHM 210
TCF7L1_chr41_Acipenser_ruthenus         L-S-NP--YL-SSGSLSTG-ARTYLQMKWPLLDVPGSG-----GLKDSRASTPGHL-SNKVPVVQHPHHM 210
TCF7L1_ste150_Acipenser_ruthenus        L-S-NP--YL-SSGSLSTG-ARTYLQMKWPLLDVPGSG-----GLKDSRASTPGHL-SNKVPVVQHPHHM 210
TCF7L1_Latimeria_chalumnae              L-T-NP--YL-SNGSLSPG-ARTYLQMKWPLLDVPGSA-----GLKDTRSPSPAHL-SNKVPVVQHPHHV 210
TCF7L1a_Lepisosteus_oculatus            L-T-NP--YL-SNGSLSPS-ARTYLQMKWPLLDVPGTA-----GLKDSRSPTPGHL-SNKVPVVQHPHHV 210
TCF7L1b_Danio_rerio                     L-A-NP--YL-SNGALSPS-ARTYLQMKWPLLDVPGSA-----ALKDSQSPSPGHL-SNKVPVVQHA-HM 210
TCF7L1_Oryzias_latipes                  L-T-NP--YL-SNGSLSPG-ARTYLHMKWPLLDVPSSA-----GIKDSRSPTPGHL-SNKVPVVQHGHHV 210
TCF7L1_Takifugu_rubripes                L-T-NP--YL-PNGSLSPS-ARTYLQMKWPLLDVPGSA-----GLKDSRSPTPGHL-SNKVPVVQHAHHV 210
TCF7L1_Tetraodon_nigroviridis           L-T-NP--YL-PNGSLSPS-ARTYLQMKWPLLDVPGSA-----GLKDSRTPTPGHL-SNKVPVVQHAHHV 210
TCF7L1a_Danio_rerio                     I-T-NP--YL-SNGSLSPS-TRTYLQMKWPLLDVPASA-----ALKDSRSPTPGHL-SNKVPVVQHPHHV 210
TCF7L1x_Tetraodon_nigroviridis          L-G-NP--YL-PNGALTPN-ARTYLPFQWPLLDVAARS-----AIRDATNPA--HL-SNNVPVVQHPHMT 210
TCF7L1x_Takifugu_rubripes               L-G-NP--YL-PNGALASN-ARTYLPFQWPLLDVAARS-----AIRDSTNAA--HL-PNNVPMVQHPHMT 210
TCF7L1x_Oryzias_latipes                 L-G-NP--YL-TSG------GRAYLPFQWPLLDVPGRT-----AVRDSATPT--HL-SS--------PMA 210
Scanicula_TCF7L1                        L-S-AP--YL-PTGSLSPG-ARTYLQMKWPLLDVQTAS-----CLKDSPSPI--HL-TNRLPLVQHAHHM 210
Amblyraja_radiata_TCF7L1_partial        -----P--Q-----GLSPG-PRTYLHMKWPLLDLQTAG-----SLKDSSSPV--HI-AAGLPLVQHPHHM 210
Chiloscyllium_plagiosum_TCF7L1          L-A-TP--YL-PSGSLTPG-ARTYLQMKWPLLDVQTAS-----CLKDSPAPI--HL-ANRLPLVQHAHHM 210
Elphany_shark_partial_TCF7L1            L-S-SP--YL-PTGALSPS-ARTYLHVKWPLLDVQAAA-----GLKDARSPPPTHL-SNKVAVVQHPHHV 210
TCF7L2_Homo_sapiens_V3                  L-T-SP--YL-PNGSLSPT-ARTYLQMKWPLLDVQAGSLQSRQALKDARSPSPAHIVSNKVPVVQHPHHV 210
TCF7L2_Mus_musculus_V3                  L-T-SP--YL-PNGSLSPT-ARTYLQMKWPLLDVQAGSLQSRQTLKDARSPSPAHIVSNKVPVVQHPHHV 210
TCF7L2_Gallus_gallus_V3                 L-S-SP--YL-PNGSLSPT-ARTYLQMKWPLLDVQAGSLQSRQALKDARSPSPAHIVSNKVPVVQHPHHV 210
TCF7L2_Taeniopygia_guttata_V3           L-S-SP--YL-PNGSLSPT-ARTYLQMKWPLLDVQAGSLQSRQALKDARSPSPAHIVSNKVPVVQHPHHV 210
TCF7L2_Anolis_carolinensis_V3           L-T-GP--YL-PNGSLSPT-ARTYLQMKWPLLDVQAGSLQSRQALKDARSPSPAHIVSNKVPVVQHPHHV 210
TCF7L2_Chrysemys_picta_bellii_V3        L-T-SP--YL-PNGSLSPT-ARTYLQMKWPLLDVQAGSLQSRQALKDARSPSPAHIVSNKVPVVQHPHHV 210
TCF7L2_Xenopus_tropicalis_V3            L-T-SP--YL-PNGSLSPT-ARTYLQMKWPLLDVQAGSLQSRQGLKDTRSPSPAHIVSNKVPVVQHPHHV 210
TCF7L2L_Xenopus_laevis_V3               L-S-SP--YL-PNGSLSPT-ARQYLQMKWPLLDVQAGSLQSRQGMKDTRSPSPAHIVSNKVPVVQHPHHV 210
TCF7L2S_Xenopus_laevis_V3               L-T-SP--YL-PNGSLSPT-ARQYLQMKWPLLDVQAGSLQSRQGIKDTRSPSPAHIVSNKVPVVQHPHHV 210
TCF7L2_chr7_Acipenser_ruthenus_V3       L-T-SP--YL-QNGSLSPT-ARTYLQMKWPLLDVQAGSLQSRQALKDARSPSPAHIVSNKVPVVQHPHHV 210
TCF7L2_chr13_Acipenser_ruthenus_V3      L-T-SP--YL-QNGSLSPT-ARTYLQMKWPLLDVQAGSLQSRQALKDARSPSPAHIVSNKVPVVQHPHHV 210
TCF7L2_Latimeria_chalumnae_V3           L-T-NP--YL-PNGSLSPT-ARTYLQMKWPLLDVQAGSLQSRQALKDARSPSPAHIVSNKVPVVQHPHHV 210
TCF7L2_Lepisosteus_oculatus_V3          L-T-SP--YL-PNGSLSPT-ARTYLQMKWPLLDVQAGGLQSRQALKDARSPSPAHIVSNKVPVVQHPHHV 210
TCF7L2_Danio_rerio_V3                   L-T-SP--YL-PNGSLSPT-ARTYLQMKWPLLDVQAGSLQSRQALKDARSPSPAHIVSNKVPVVQHPHHV 210
TCF7L2_Tetraodon_nigroviridis_V3        L-T-SP--YL-PNGSLSPT-ARTYLQMKWPLLDVQPGGLQSRQALKDARSPSPAHIVSNKVPVVQHPHHV 210
TCF7L2_Oryzias_latipes_V3               L-T-SP--YL-PNGSLSPT-ARTYLQMKWPLLDVQPGGLQSRQALKDARSPSPAHIVSNKVPVVQHPHHV 210
TCF7L2_Takifugu_rubripes_V3             L-T-SP--YL-PNGSLSPT-ARTYLQMKWPLLDVQPGGLQSRQALKDARSPSPAHIVSNKVPVVQHPHHV 210
Amblyraja_radiata_TCF7L2                L-A-SP--YL-PNGSLSPS-ARTYLQMKWPLIDVQAGSLQGRQGLKDARSPSPAHIVSNKVPVVQHPHHV 210
Chiloscyllium_plagiosum_TCF7L2          L-A-SP--YL-PNGSLSPS-ARTYLQMKWPLIDVQAGSLQGRQGLKDARSPSPAHIVSNKVPVVQHPHHV 210
Scanicula_TCF7L2                        L-A-SP--YL-PNGSLSPS-ARTYLQMKWPLIDVQAGSLQGRQGLKDARSPSPAHIVSNKVPVVQHPHHV 210
Elephant_shark_TCF7L2                   L-A-SP--YL-PNGSLSPS-ARTYLQMKWPLIDVQAGSLQGRQGLKDARSPSPAHIVSNKVPVVQHPHHV 210
Lamprey_TCF_chr41                       L-A-NP--YL-ANGSLSPS-ARSYLPLKWPLFDMQAPL-PGRKAGDDSRSPSPAYLISNKVPVVQSPHHV 210
Pacific_lamprey_TCF_chr43               L-G-NP--YL-ANGSLSPS-ARSYLPLKWPLFDMQAPL-PGRKAGDDSRSPSPAYLISNKVPVVQSPHHV 210
Lethenteron_reissneri_chr47_TCF         L-G-NP--YL-ANGSLSPS-ARSYLPLKWPLFDMQAPL-PGRKAGDDSRSPSPAYLISNKVPVVQSPHHV 210
Lamprey_TCF_chr8                        L-G-TP--CL-SQGALSPP----YVHLGWRLCDVRPGY-GSDRQESAALVRSPSYPAPNKVPVVQSPHHV 210
Pacific_lamprey_TCF_chr22               L-G-TP--CL-SQGALSPP----YVHLGWRLCDVRPGY-GSGRQESAAPVRSPSYPAPNKVPVVQSPHHV 210
```

```
Lethenteron_reissneri_chr34_TCF        L-G-TP--CL-SQGALSPP----YVHLGWRLCDVRPGY-GSGRQESAAPVRSPSYPAPNKVPVVQSPHHV 210
Lamprey_putative_TCF_chr68_partial     L-A-GP--YL-SNGSLSPS-ART----------------------------------GNKM--MQSLAHV 210
Ciona_robusta_TCF_v3                   I-G---------AAIA---------------------------------------LNQQSLLSS---- 210
Ciona_intestinalis_TCF                 I-G---------AAIA---------------------------------------LNQQSLLSS---- 210
Ciona_savignyi_TSA_based               I-G---------AALA-----------------------------------------QQSLLSN---V 210
Phallucia_fumigata_TCF                 V-P----------SM-------------------------------------------YSL------P 210
Halocynthia_roretzi_TCF                I-G----------SIVS-------------------------------------SLHPSN-LF---N 210
B_lanceolatum_TCF                      I-VSNMG-YPHPNGSLAAS--AT----------------------------------SNKVSVVHGPPHP 210
B_belcheri_TCF                         I-VSNMG-YPHPNGSLAAS--AT----------------------------------SNKVSVVHGPPHP 210
B_floridae_TCF                         I-VSNMG-YPHPNGSLAAS--AT----------------------------------SNKVSVVHGPPHP 210
Saccoglossus_kowalevskii_TCF           LHGDLHV-YP-PNGSMVSMKRFIDIAANFLRPADYLDHLEDQVQ------------TNKVSVVHPAGHP 210
Ptychodera_flava_TCF_TSA               LHGDLHV-YP-PNGSMVSM-------------------------------------ANKVSVVHPPGHP 210
Strongylocentrotus_purpuratus_TCF      LAIANAG-Y--PNGSMVGE-------------------------------------GQKMPVLHP-GYP 210
Asterias_rubens_TCF                    LAIA-GG-Y-HPNGSMVSS-------------------------------------GSKLSMVHPSGYP 210
Apostichopus_japonicus_TCF             --IAFPGNY-HSNGAVL---------------------------------------GSKVPMVHPMAYP 210


LEF1_Homo_sapiens_V2                   HPLTPLITYSDEHFSPGSHPSHI-PSDVNSKQGMSRHPPAP-DIPTFYPLSPGGVGQITPPLG-W----Q 280
LEF1_Mus_musculus_V2                   HPLTPLITYSDEHFSPGSHPSHI-PSDVNSKQGMSRHPPAP-EIPTFYPLSPGGVGQITPPIG-W----Q 280
LEF1_Gallus_gallus_V2                  HPLTPLITYSDEHFSPGSHPSHI-PSDVSSKQGMSRHPPAP-DIPTFYPLSPGGVGQITPPLG-W----Q 280
LEF1_Taeniopygia_guttata_V2            HPLTPLITYSDEHFSPGSHPSHV-PSDVSSKQGMSRHPPAP-DLPTFYPLSPGGVGQITPPLG-W----Q 280
LEF1_Anolis_carolinensis_V2            HPLTPLITYSDEHFSPGTHPSHL-PSDVNSKQGMSRHPPGP-DMPTFYPLSPGSVGQITPPLG-W----Q 280
LEF1_Chrysemys_picta_bellii_V2         HPLTPLITYSDEHFSPGSHPSHI-PSDVNSKQGMSRHPPAP-DIPTFYPLSPGGVGQITPPLG-W----Q 280
LEF1_Xenopus_tropicalis_V2             HPLTPLITYSDEHFAPGAHPSHI-PSDVNSKQGMHRHPQAP-DLPTFYPLSPGSVGQMTPPLG-W----- 280
LEF1L_Xenopus_laevis_V2                HPLTPLITYSDEHFAPGVHPSHI-PSDINTKQGMHRHPQAP-DLPTFYPMSPGSVGQMTPPLG-W----- 280
LEF1S_Xenopus_laevis_V2                HPLTPLITYSDEHFAPGAHPSHL-PSDVNTKQGMHRHHQVP-DLPTFYPLSPGSVGQMTPPLG-W----- 280
LEF1_chr1_Acipenser_ruthenus_V2        HPLTPLITYSDEHFAPGPHSGHH-PQEVNSKQGIHRHHPGP-DMANFYSLSPGGVGQITPPLG-W----Q 280
LEF1_chr2_Acipenser_ruthenus_V2        HPLTPLITYSDEHFAPGPHSGHH-PQEVDSKQGIHRHHPGP-DMANFYPLSPGGVGQITPPLG-W----Q 280
LEF1_Latimeria_chalumnae_V2            LP-------------------------NVRQGMPRHPPGP-DIPTFYPLSPGGVGQITPPLG-W----Q 280
LEF1_Lepisosteus_oculatus_V2           HPLTPLITYSDEHFAPGPHSGHH-PQDVNSKQGMPRHHPGP-DIANFYPLSPGGVGQITPPLG-W----Q 280
LEF1_Danio_rerio_V2                    HPLTPLITYSDEHFAPGPHSGHH-PQDVNPKQGMPRHHPGP-DIPNFYPLSPGGVGQMTPPLG-W----- 280
LEF1_Oryzias_latipes_V2                HPLTPLITYSDEHFAPGSHSGLH-PQDANNKQGMPRHHPGP-DMPNFYSLSPGGVGQITPPLG-W----- 280
LEF1_Tetraodon_nigroviridis_V2         HPLTPLITYSDEHFAPGSHSGHH-PHDGSSKQGMPRQHPGP-DMPNFYSLSPGGVGQITPPLG-W----- 280
LEF1_Takifugu_rubripes_V2              HPLTPLITYSDEHFAPGSHSGHH-PHDGSNKQGMPRQHPGP-DMANFYSLSPGGVGQITPPLG-W----- 280
Amblyraja_radiata_LEF1                 HPLTPLITYSDEHFAPGSHPTQI-PTDTSSKRGMHRHPPGP-DIPTFYPLSPGTIGQLTPPLS-W----Q 280
Chiloscyllium_plagiosum_LEF1           HPLTPLITYSDEHFAPGSHPTQI-PTDINSKRGMQRHPAGP-DIPSFYPLSPGAVGQLTPPLN-W----- 280
Scanicula_LEF1                         HPLTPLITYSDEHFAPGSHPAQI-PTDINSKRGMHRHPPGP-DITSFYPLSPGAVGQLTPPLN-W----- 280
Elphany_shark_partial_LEF1             HPLTPLITYSDEHFAPGVHPTHI-PTDVNSKRGIHRHPPGP-EMPTFYPLSPGGVGQLTPPLG-W----Q 280
TCF7_Homo_sapiens_V3                   PQLS-L----YEHFNSPHPTP-A-PADISQKQ-VHRPLQTP-DLSGFYSLTSGSMGQLPHTVS-W----P 280
TCF7_Mus_musculus_V3                   PQLSPL----YEHFSSPHPTP-A-PADISQKQGVHRPLQTP-DLSGFYSLTSGSMGQLPHTVS-W----P 280
TCF7_Gallus_gallus_V3                  HPLTPLIPYSNDHFSHGSHSPHL-PADISQKQAVHRPSQTS-DIPGFYPLPPAGVGQITPSMG-W----Q 280
TCF7_Taeniopygia_guttata_V3            HPLTPLIPYSNDHFSHGSHSPHL-PADINQKQGVHRPSQTS-DIPGFYPLPPGGVGQITPSMG-W----Q 280
TCF7_Anolis_carolinensis_V3            HPLTPLIPYSNEHFSHGSHSPHL-PADISQKQGVHRPSQTS-DIPGFYSLPPTGVGQITSSMG-W----Q 280
TCF7_Chrysemys_picta_bellii_V3         HPLTPLIPYSNEHFSHGSHSPHL-PADINQKQGVHRPSQTS-DIPGFYSLPPGGVGQITPSMG-W----Q 280
TCF7_Xenopus_tropicalis_V3             H---PLIPYNNESFSHGSHSTHL-PADLNQKQGVHRPAQTA-DIPTFYPLPSGGVGQISPSVG-W----Q 280
TCF7S_Xenopus_laevis_V3                H---PLIPYNSESFSHGSHSPHL-PVDLNQKQGVHRPSQTP-DVPTFYPLPSGGVGQISPSMG-W----Q 280
TCF7_chr15_Acipenser_ruthenus_V3       HPLTSLLPYSNDHFNPNS--PHL-PADMSQKPGVHRP-QSQ-DLSGFYSLPPGGVGQITPPMG-W----Q 280
TCF7_chr29_Acipenser_ruthenus_V3       HPLTSLLPYSNDHFNPNS--PHL-PADMSQKPGVHRP-QSQ-DLSGFYSLPPGGVGQITPPMG-W----Q 280
TCF7_Latimeria_chalumnae_V3            HPLTPLIPYSNEGFSTGSHSPHL-PADINQKQGIHRPAQCP-DIPGFYSLPPSGVGQITPPMG-W----Q 280
TCF7_Lepisosteus_oculatus_V3           HPLTPLLPYSNDHFNPSS--PHL-PTDMSQKPGVHRP-PSQ-EISGFYSLPPAGVGQITPSMS-W----Q 280
TCF7_Danio_rerio_V3                    HPLTPLLPY--EHFNPSP--THM-PTDGGQKPGVHRH-QTQ-EISGFYSLPQ---GQITPSMN-W----P 280
TCF7_Oryzias_latipes_V3                HPLTSLLPYSNDHFSPSP--PHL-PTDMSQKTGVHRH-QSQ-DLSGYYSLPPGGVGQIPPSMS-W----Q 280
TCF7_Takifugu_rubripes_V3              HPLSSLLPYGNEHFSPSP--PHL-PADMSQKPGVHRH-QSQ-DLSGYYSLPPGGVGQITPSMS-W----Q 280
TCF7_Tetraodon_nigroviridis_V3         HPLSSLLPYGNEHFSPSP--PHL-PADMSQKPGVHRH-QSQ-DLSGYYSLPPGGVGQISPSVS-W----Q 280
Amblyraja_radiata_TCF7                 HPLTPLISYSNEHFPSNPHSPHL-SSEMNQKTGLHRSPQGP-DLAAYYSMSPGSVGQMTPAMG-W----Q 280
Chiloscyllium_plagiosum_TCF7           HPLTPLISYSNEHFAPSSHSPHL-PAEINQKSGLHRSPQGP-DLAALYSLSPGTAGQITPTIG-W----Q 280
Scanicula_TCF7                         HPLTPLISYSNEHFAPSSHSPHL-PAEINQKTGLHRSPQGP-ELAALYSLSPGSVGQITPTVG-W----Q 280
Elphany_shark_partial_TCF7             HPLTPLISYSNEHFSPSSHSPHL-PADINQKTGMHRPHQGP-DLATFYSLSPGGVGQISPAMG-W----Q 280
TCF7L1_Homo_sapiens                    HPLTPLITYSNDHFSPGSPPTHL-SPEIDPKTGIPRPPHPS-ELSPYYPLSPGAVGQIPHPLG-WLVPQQ 280
TCF7L1_Mus_musculus                    HPLTPLITYSNDHFSPASPPTHL-SPEIDPKTGIPRPPHPS-ELSPYYPLSPGAVGQIPHPLG-WLVPQQ 280
TCF7L1_Gallus_gallus                   HPLTPLITYSNDHFSPGSPPAHL-SPDIDPKTGIPRPPHPP-ELPPYYPLSPGAVGQIPHPLG-WLVPQQ 280
TCF7L1_Taeniopygia_guttata             HPLTPLITYSSEPFPPGSPPGHL-SPDIDPKTGIPRPPHPP-ELPPYYPLSPGAVGQLPHPLG-WLVPQQ 280
TCF7L1_Anolis_carolinensis             HPLTPLITYSNDHFSPGSPPSHL-SPEIDPKTGIPRPPHPS-ELSPYYPLSPGAIGQIPHPLG-WIVPQQ 280
TCF7L1_Chrysemys_picta_bellii          HPLTPLITYSSDHFSPGSPPSHL-SPEIDPKTGIPRPPHPS-ELSPYYPLSPGAVGQIPHALG-WLVPQQ 280
TCF7L1_Xenopus_tropicales              HPLTPLITYSNEHFSPGTPPGHL-SPEIDPKTGIPRPPHPS-ELSPYYPLSPGAVGQIPHPLG-WLVPQQ 280
TCF7L1L_Xenopus_laevis                 HPLTPLITYSNEHFSPGTPPGHL-SPEIDPKTGIPRPPHPS-ELSPYYPLSPGAVGQIPHPLG-WLVPPQ 280
TCF7L1S_Xenopus_laevis                 HPLTPLITYSNEHFSPGTPPGHL-SPEIDPKTGIPRPPHPS-ELSPYYPLSPGAVGQIPHPLG-WLVPQQ 280
TCF7L1_chr41_Acipenser_ruthenus        HPLTPLITYSNEHFSPGTPPPHL-SPEIDPKTGIPRTPHPS-ELSPYYPLSPGAVGQIPHPLG-WLVPQQ 280
TCF7L1_ste150_Acipenser_ruthenus       HPLTPLITYSNEHFSPGTPPPHL-SPEIDPKTGIPRTPHPS-ELSPYYPLSPGAVGQIPHPLG-WLVPQQ 280
TCF7L1_Latimeria_chalumnae             HPLTPLITYSNEHFSPGTPPSHL-SPEISPKTGIPRHPHPS-ELSPYYPLSPGAVGQIPHPLG-WLVPQQ 280
TCF7L1a_Lepisosteus_oculatus           HPLTPLITYSNEHFSPGTPPSHL-SPEIDPKTGIPRAPHPS-ELSPYYPLSPGAVGQIPHPLG-WLVPQQ 280
TCF7L1b_Danio_rerio                    HPLTPLITYSNE-FPPGTPPAHL-SPEIDPKTGIPRTPHPA-ELSPYYPLSPGAVGQIPHPLG-WLVPQQ 280
TCF7L1_Oryzias_latipes                 HPLTPLITYSNEHFSPGTPPSHL-SPEIDPKTGIPRTPHPS-ELSPYYPLSPGAVGSIAHPLS-WFMQHQ 280
TCF7L1_Takifugu_rubripes               HPLTPLITYSNEHFSPGTPPSHL-SPEIDPKTGIPRTPHPS-ELSPYYPLSPGAVGSIAHPLG-WFMPQQ 280
TCF7L1_Tetraodon_nigroviridis          HPLTPLITYSNEHFSPGTPPSHL-SPEIDPKTGIPRTPHPS-ELSPYYPLSPGAVGSIAHPLG-WFMPQQ 280
TCF7L1a_Danio_rerio                    HPLTPLITYSNEHFSPGTPPSHL-SPEIDPKTGIPRTPHPS-ELSPYYPLSPGAVGQIPHPLG-WLVPQQ 280
TCF7L1x_Tetraodon_nigroviridis         H-LHPLLSYSPEAFSPQRASPGF-SPDT----GVSRSPHAA-----CYPVSPGGMAQITHPLG-WL---P 280
TCF7L1x_Takifugu_rubripes              H-LHPLLSYSPEAFSPQRASPGF-SPDT----GISRSPHTA-----CYPVSPGGMAQITHPLG-WL---P 280
```

```
TCF7L1x_Oryzias_latipes                 H-LHPLLSYSPEAFSPQRASPVL-SPDA----GMSRTPHAA-----CYPLSPGGMAQMAPPLG-WL---S 280
Scanicula_TCF7L1                        HPLTPLITYSNEHFSPGNPTSHL-SSELDSKAGLSRVPHPS-ELSPYYPLSPGAVGQIAHPLS-WIMPPQ 280
Amblyraja_radiata_TCF7L1_partial        HPLTPLITYSNDHFTPG----SL-SPELDPKTGLPRLRHPS-DLAAFYPLAAGGVGQIAHPLG-WVVPPP 280
Chiloscyllium_plagiosum_TCF7L1          HPLTPLITYSNEHFSPGNPTPHL-SSELNSKTGLSRVPYPS-ELSPYYPLSPGTVGPIAHPLG-WVMPPQ 280
Elphany_shark_partial_TCF7L1            HPLTPLITYSNEHF-PGNPSPHL-SPDLDPKTGIPRPPHPS-ELSPYYPLSPGAVGQLTHPLG-WLVPPQ 280
TCF7L2_Homo_sapiens_V3                  HPLTPLITYSNEHFTPGNPPPHL-PADVDPKTGIPRPPHPP-DISPYYPLSPGTVGQIPHPLG-WLVPQQ 280
TCF7L2_Mus_musculus_V3                  HPLTPLITYSNEHFTPGNPPPHL-PADVDPKTGIPRPPHPP-DISPYYPLSPGTVGQIPHPLG-WLVPQQ 280
TCF7L2_Gallus_gallus_V3                 HPLTPLITYSNEHFTPGNPPPHL-PADVDPKTGIPRPPHPP-DISPYYPLSPGTVGQIPHPLG-WLVPQQ 280
TCF7L2_Taeniopygia_guttata_V3           HPLTPLITYSNEHFTPGNPPPHL-PADVDPKTGIPRPPHPP-DISPYYPLSPGTVGQIPHPLG-WLVPQQ 280
TCF7L2_Anolis_carolinensis_V3           HPLTPLITYSNEHFTPGNPPPHL-PADVDPKTGIPRPPHPP-DISPYYPLSPGAVGQIPHPLG-WLVPQQ 280
TCF7L2_Chrysemys_picta_bellii_V3        HPLTPLITYSNEHFTPGNPPPHL-PADVDPKTGIPRPPHPP-DISPYYPLSPGTVGQIPHPLG-WLVPQQ 280
TCF7L2_Xenopus_tropicalis_V3            HPLTPLITYSNEHFTPGNPPPHL-QADVDPKTGIPRPPHPP-DISPYYPLSPGAVGQIPHPLG-WLVPQQ 280
TCF7L2L_Xenopus_laevis_V3               HPLTPLITYSNEHFTPGNPPPHL-QADVDPKTGIPRPPHPP-DISPYYPLSPGAVGQIPHPLG-WLVPQQ 280
TCF7L2S_Xenopus_laevis_V3               HPLTPLITYSNEHFTPGNPPPHL-QADLDPKTGIPRPPHPP-DISPYYPLSPGTMGQIPHPLG-WLVPQQ 280
TCF7L2_chr7_Acipenser_ruthenus_V3       HPLTPLITYSNEHFTPGNPPPHL-QADVDPKTGIPRPPH-P-DISPYYPLSPGTVGQIPHPLG-WLVPQQ 280
TCF7L2_chr13_Acipenser_ruthenus_V3      HPLTPLITYSNEHFTPGNPPPHL-QADVDPKTGIPRPPH-P-DISPYYPLSPGTVGQIPHPLG-WLVPQQ 280
TCF7L2_Latimeria_chalumnae_V3           HPLTPLITYSNEHFTPGNPPPHL-PADVDPKTGIPRPPHPP-DISPYYPLSPGTVGQIPHPLG-WLVPQQ 280
TCF7L2_Lepisosteus_oculatus_V3          HPLTPLITYSNEHFTPGNPPPHL-QTDVDPKTGIPRPPHPP-DISPYYPLSPGTVGQIPHPLG-WLVPQQ 280
TCF7L2_Danio_rerio_V3                   HPLTPLITYSNEHFTPGNPPPHL-QADVDPKTGIPRPPHPP-DISPYYPLSPGTVGQIPHPLG-WLVPQQ 280
TCF7L2_Tetraodon_nigroviridis_V3        HPLTPLITYSNEHFTPGNPPPHL-QTDVDPKTGIPRPPHPP-DISPYYPLSPGTVGQIPHPLG-WLVPQQ 280
TCF7L2_Oryzias_latipes_V3               HPLTPLITYSNEHFTPGNPPPHL-QTDVDPKTGIPRPPHPP-DISPYYPLSPGTVGQIPHPLG-WLVPQQ 280
TCF7L2_Takifugu_rubripes_V3             HPLTPLITYSNEHFTPGNPPPHL-QTDVDPKTGIPRPPHPP-DISPYYPLSPGTVGQIPHPLG-WLVPQQ 280
Amblyraja_radiata_TCF7L2                HPLTPLITYSNEHFTPGNPPPHL-PADVDPKTGIPRPPHPS-DISPYYPLSPGAVGQIPHPLG-WLVPQQ 280
Chiloscyllium_plagiosum_TCF7L2          HPLTPLITYSNEHFTPGNPPPHL-PADVDPKTGIPRPPHPS-DISPYYPLSPGTVGQIPHPLG-WLVPQQ 280
Scanicula_TCF7L2                        HPLTPLITYSNEHFTPGNPPPHL-PADVDPKTGIPRPPHPS-DISPYYPLSPGAVGQIPHPLG-WLVPQQ 280
Elephant_shark_TCF7L2                   HPLTPLITYSNEHFTPGNPPPHL-PADVDPKTGIPRPPHPS-DISPYYPLSPGTVGQIPHPLG-WLVPQQ 280
Lamprey_TCF_chr41                       HPLAPILTYGGERFVPGTPPP-I-PTEVDPKTGLPRPSHTP-ELSPYYPLSPGAMGQIPH-LG-W----Q 280
Pacific_lamprey_TCF_chr43               HPLAPILTYGGERFVPGTPPP-I-PTEVDPKTGLPRPGHTP-ELSPYYPLSPGAMGQIPH-LG-W----Q 280
Lethenteron_reissneri_chr47_TCF         HPLAPILTYGGERFVPGTPPP-I-PTEVDPKTGLPRPGHTP-ELSPYYPLSPGAMGQIPH-LG-W----Q 280
Lamprey_TCF_chr8                        HPLTPLLAYSNEHFAPGTPSPHL--ADLDPKIGIPRPPHGP-DLASYYSLP----GQIPHPLC-W----Q 280
Pacific_lamprey_TCF_chr22               HPLTPLLAYSNEHFAPGTPSPHL--ADLDPKTGIPRPPHGP-DLASYYSLP----GQIPHPLC-W----Q 280
Lethenteron_reissneri_chr34_TCF         HPLTPLLAYSNEHFAPGTPSPHL--ADLDPKTGIPRPPHGP-DLASYYSLP----GQIPHPLC-W----Q 280
Lamprey_putative_TCF_chr68_partial      HPLSPLLTYGAE-FTPTSTPPP------DGKTVTVRGGYGGGDLSQYHPLSP---RPIAHPFG-WVFNQQ 280
Ciona_robusta_TCF_v3                    -LPSSSQPYNPYAYHHFLSPTDI-----DGKPGLPRHN---DVPAMY-PLPG-QLGQIPNPLSPWASAMA 280
Ciona_intestinalis_TCF                  -LPSSSQPYNPYAYHHFLSPTDI-----DGKPGIPRHN---DAPAMY-PLPG-QLGQIPHPLSPWASAMA 280
Ciona_savignyi_TSA_based                GLPSSSQPYNPYAYHHLLSPSDI-----DTKPGIPHGAR--DVPAMY-ALPG-QLGQIPHPLTPWASAMA 280
Phallucia_fumigata_TCF                  GQLG--QPN-PLA--PPWSPG---------------------------------------PL---ASGVA 280
Halocynthia_roretzi_TCF                 GVPPNGKPY-PYSMHPLLSP-DI-----DSKTGLPRSHHP-DIPSMY-SLPG-QLGQIPHPLAPWASTMA 280
B_lanceolatum_TCF                       GPLTQLMMYSNEHFAPGT-PPHINSIEVDPKTGIPRPRHPADLPPMY-AIPPNQLPQLPK-IEEW----Q 280
B_belcheri_TCF                          GPLTQLMMYSNEHFAPGT-PPHINSIEVDPKTGIPRPRHPADLPPMY-AIPPNQLPQLPK-IEEW----Q 280
B_floridae_TCF                          GPLTQLMMYSNEHFAPGT-PPHINSIEVDPKTGIPRPRHPADLPPMY-AIPPNQLPQLPK-IEEW----Q 280
Saccoglossus_kowalevskii_TCF            NPLAQLLMYNNDHFPPGTTPPHLSPLELDPKTGMPRPRHPGEMPPIY-PPLPGQMGQIHHPMEVW---QP 280
Ptychodera_flava_TCF_TSA                NPLAQLLMYNNDHFPHGTTPPHLSPMELDPKASMPRPRHPGEMPPLY-PPLPGQMGQLPHHMEVW---QP 280
Strongylocentrotus_purpuratus_TCF       PSLAQLMMYQQEHYSPGT-PPHVG-LEVDSKTGILRRSH-SEISPYH-PLSPSSLGT--HPLEVW----P 280
Asterias_rubens_TCF                     PSLAHLMMYNQEHYPPGTPPPHLGTIEVDAKTGIPRPRHSSELPLYH-PLSPGSLGSVPHHLEVW----Q 280
Apostichopus_japonicus_TCF              PSLAHL-MYNSDRYPPGTPPPHLSSVEIDSKTGILRPRHSLDLPSYH-PLSPSSLGQITPHLDV------ 280
                                                                                     .

LEF1_Homo_sapiens_V2                    GQPVYPI-TGGFRQPYPSSLSVD-TSMS------RFSHHMIPG-PPGPHTTGIPHPAIVTPQVKQEHPHT 350
LEF1_Mus_musculus_V2                    GQPVYPI-TGGFRQPYPSSLSGD-TSMS------RFSHHMIPG-PPGPHTTGIPHPAIVTPQVKQEHPHT 350
LEF1_Gallus_gallus_V2                   GQPVYPI-SSGFRQPYPSSLSVD-PSMS------RFSHHMIPG-PPGPHTTGIPHPAIVTPQVKQEHPHN 350
LEF1_Taeniopygia_guttata_V2             GQPVYPI-SSGFRQPYPSSLSVD-PSMS------RFSHHMIPG-PPGPHTTGIPHPAIVTPQVKQEHLHN 350
LEF1_Anolis_carolinensis_V2             GQPVYPI-SSGFRQPYPSSLSVD-TSMS------RFSHHMIPG-PPGPHTTGIPHPAIVTPQVKQEHPHT 350
LEF1_Chrysemys_picta_bellii_V2          GQPVYPI-SSGFRQPYPSSLSVD-PSMS------RFSHHMIPG-PPGPHATGIPHPAIVTPQVKQEHPHT 350
LEF1_Xenopus_tropicalis_V2              -----------------------------------YPHHMVSG-PPGPHATGIPHPAIVNPQVKQEHPHN 350
LEF1L_Xenopus_laevis_V2                 -----------------------------------YPHHMVSG-PPGPHATGIPHPAIVNPQVKQEHSHN 350
LEF1S_Xenopus_laevis_V2                 -----------------------------------YPHHMVSG-PPGHHATGIPHPAIVNPQVKQEHPHN 350
LEF1_chr1_Acipenser_ruthenus_V2         GQPMYPI-SSGFRQQYPSTLPSD-TPMS------RFSHHMVPG-PPGPHATGIPHPAIVNPQVKHEHPH- 350
LEF1_chr2_Acipenser_ruthenus_V2         GQPMYPI-SSGFRQQYPSTLPGD-TPMS------RFSHHMVPG-PPGPHATGIPHPAIVNPQVKHEHPH- 350
LEF1_Latimeria_chalumnae_V2             GQPVYPI-TSGFRQPYPSPLSVD-TSMS------RFSHHMVPG-PPGPHATGIPHPAIVNPQIKQEPQHN 350
LEF1_Lepisosteus_oculatus_V2            GQPVYPI-SGGFREPYPSALPVD-TPMA------RFSHHMVPG-PPGPHTTGIPHPAIVNPQVKHEHPH- 350
LEF1_Danio_rerio_V2                     -----------------------------------FSHHMVPG-PPGPHATGIPHPAIVNPQVKQE--H- 350
LEF1_Oryzias_latipes_V2                 -----------------------------------FSHHMVPG-PPGPHATGIPHPAIVNPQIKHEPPH- 350
LEF1_Tetraodon_nigroviridis_V2          -----------------------------------FSHHMVPG-PPGPHATGIPHPAIVNPQIKHEPLH- 350
LEF1_Takifugu_rubripes_V2               -----------------------------------FSHHMVPG-PPGPHATGIPHPAIVNPQIKHEPLH- 350
Amblyraja_radiata_LEF1                  GQPVYPITTGGFRHPYPTALTVN-ASMS------RFTHPMVPT-HP----SGIPHPAMENHQIKQEPMHS 350
Chiloscyllium_plagiosum_LEF1            ----------------------------------RFSHPMVHG-NPNPPTTGIPHPAIVNHQIKQEPIHN 350
Scanicula_LEF1                          -----------------------------------FSHPMVPG-NH--QTTGIPHPAIVNHQIKQEPIH- 350
Elphany_shark_partial_LEF1              GQPIYP-VSGGFRQPYPTAHPVN-TPIS------RFSHHMMPG-QPGHHATGIPHPAIVNPQIKQEPMQN 350
TCF7_Homo_sapiens_V3                    SPPLYPSPSCGYRQHFPAPTAAPGAPYP------RFTHPSMLG-SGVPHPAAIPHPAIVPPSGKQELQPF 350
TCF7_Mus_musculus_V3                    SPPLYPSPSCGYRQHFPAPTAAPGAPYP------RFTHPSMLG-SGVPHPAAIPHPAIVPSSGKQELQPY 350
TCF7_Gallus_gallus_V3                   SQSVYPLPSCGFRQPYSSALSAG-ASFS------RFTHPLMLG--SGMHTTGIPHPAIVPHSGKQEMEHY 350
TCF7_Taeniopygia_guttata_V3             SQSVYPLPSCGFRQPYSSALSAG-ASFS------RFTHPLMLG--SGMHTTGIPHPAIVPHSGKQEMEHY 350
TCF7_Anolis_carolinensis_V3             SQPVCPFQSCGFRQPYTSALSAG-ATFS------RITHPLMLG--SGMHTTGISHPGIT-SPGKQEMDHY 350
TCF7_Chrysemys_picta_bellii_V3          SQSVYPLPSCGFRQPYSSALSAG-ATFS------RFTHPLMLS--SGMHTTGIPHPAIVPHSGKQEMDHY 350
TCF7_Xenopus_tropicalis_V3              SQPVYPIPSCGFRQPYSATLSSG-PSYS------RFPHPLMLS--PSMHTTGIPHPAIIPHSGNKDIDCY 350
TCF7S_Xenopus_laevis_V3                 SQPVYPIPSCGFRQPYSATMSSG-PSYS------RFPHPLMLS--PSMHTTGIPHPAIIPHSGNKDMDIY 350
TCF7_chr15_Acipenser_ruthenus_V3        SQPVYPIAPCGFRQPYSSGLPAG-SSFS------RFPHSLMLG-PPGMHSTGIPHPAIVPPSGKHEHDQY 350
```

```
TCF7_chr29_Acipenser_ruthenus_V3        SQPVYPIAPCGFRQPYSSGLPAG-SSFS------RFPHSLMLG-PPGMHSTGIPHPAIVPPSGKHEHDQY 350
TCF7_Latimeria_chalumnae_V3             SQPVYPITSCGFRQTYTSALSAG-TSLA------RFSHPLVLG-PPGMHTTGIPHPAIVPTSGKQEMDHY 350
TCF7_Lepisosteus_oculatus_V3            SQPVYPISSCGFRQPYSSSLPTS-SSFS------RFPHSLVLG-PSSMHPTGIPHPAIVPPTGKQEHDQY 350
TCF7_Danio_rerio_V3                     NQPVYPLPSCGFRQPFSSGLQSG-SSYP------RFSHSLML--QSGMHPTGIPHPAIVPPSGKQEHDQF 350
TCF7_Oryzias_latipes_V3                 SQPVYPLSPCGFRQSYSSNLPSA-SSYS------RFPHSLMLG-PSGMHPTGIPHPAIVPPSGKQEHDQY 350
TCF7_Takifugu_rubripes_V3               GQPVYPLSPCGFRQPYTSNLSSTPSSFS------RFPHPLMLG-PSGMHPTGIPHPAIVPPTGKQDHDQY 350
TCF7_Tetraodon_nigroviridis_V3          GQAVYPLSPCGFRQPYASNLSSTPSSYS------RFPHPLMLG-PSGMHPTGIPHPAIVPPTGKQEHDQY 350
Amblyraja_radiata_TCF7                  GQPVYPITTCGFRHPYPTALTVN-ASMS------RFPHPLVLG-PPGIHSTGIPHSAIMSTSGKSDMNQY 350
Chiloscyllium_plagiosum_TCF7            GQSVYPITSCGFRQPYSSPLPPS-TSIS------RFPHPLVLG-PPGMHSTGIPHPAIVSASGKPDMNQY 350
Scanicula_TCF7                          GQSVYPITSCGFRQPYSSPLPPS-TSIS------RFPHPLVLG-PPGMHSTGIPHPAIVSASGKPDMNQY 350
Elphany_shark_partial_TCF7              GQSVYPITSCGFRQPYSTALPPS-SSIS------RFSHPLVIG-PPGMHSTGIPHPAIVTTSSKAEMNQY 350
TCF7L1_Homo_sapiens                     GQPMYSLPPGGFRHPYP-ALAMN-ASMSSLVS-SRFSPHMVAPAHPGLPTSGIPHPAIVSPIVKQEPAPP 350
TCF7L1_Mus_musculus                     GQPMYSLPPGGFRHPYP-ALAMN-ASMSSLVS-SRF-PHMVAPAHPGLPTSGIPHPAIVSPIVKQEPAAP 350
TCF7L1_Gallus_gallus                    GQPMYSIPPGGFRHPYP-ALAMN-ASMSSLMS-SRFSPHMVPPPTHGLHPSGIPHPTIVSPIVKQESAQP 350
TCF7L1_Taeniopygia_guttata              GQPVYSIPAGGFRHPYP-ALAMN-ASMSSLMS-SRFSPHMVPPPAHGLHPSGIPHPTIVSPIVKQEPSQP 350
TCF7L1_Anolis_carolinensis              GQPVYSIPPGGFRHPYP-ALAMN-ASMSSLMS-SRFSPHMVPPPAHGLHPSGIPHPTIVSPIVKQEPTQP 350
TCF7L1_Chrysemys_picta_bellii           GQPVYSIPPGGFRHPYP-ALAMN-ASMSSLMS-SRFSPHMVPPAAHGLHPSGIPHPTIVSPIVKQESTQA 350
TCF7L1_Xenopus_tropicales               GQPMYSIPPGGFRHPYP-ALAMN-ASMSSLVS-SRFSPHMVPPPHHGLHTSGIPHPAIVSPIVKQEPSSG 350
TCF7L1L_Xenopus_laevis                  GQPMYSIPPGGFRHPYP-ALAMN-ASMSSLVS-SRFSPHMVPPPHHSLHTSGIPHPAIVSPIVKQEPSSG 350
TCF7L1S_Xenopus_laevis                  GQPMYSIPPGGFRHPYP-ALAMN-ASMSSLVS-SRFSPHMVPPPHHSLHTSGIPHPAIVSPIVKQEPSSG 350
TCF7L1_chr41_Acipenser_ruthenus         GQPMYSIPPGGFRHPYP-ALAMN-ASMSSLVS-SRFSPHMVSHHPHSLHQTGIPHPAIVSPAIKQEPNQG 350
TCF7L1_ste150_Acipenser_ruthenus        GQPMYSIPPGGFRHPYP-ALAMN-ASMSSLVS-SRFSPHMVSHHPHSLHQTGIPHPAIVSPAIKQEPNQG 350
TCF7L1_Latimeria_chalumnae              SQPVYSIPPGGFRHPYP-ALAVN-ASMSSLVS-SRFSPHMVPPHPHGLHTTGIPHPAIVSPAIKQEQTQG 350
TCF7L1a_Lepisosteus_oculatus            GQPMYSIPPGGFRHPYP-ALAMN-ASMSSLVS-SRFSPHMVPHPPHSLHQTGIPHPAIVSPAIKQEPNHG 350
TCF7L1b_Danio_rerio                     GQHMYPITAGGFRHPYP-ALAMN-ASMSSLVS-SRFSPHLVPHHPHGLHQTGIPHPAIVSPVIKQEPNGE 350
TCF7L1_Oryzias_latipes                  GQPMYSIPPGGFRHPYP-ALAMN-ASMSSLVS-SRFSPHMVTPPPHSLHQTGIPHPAIVSPAIKQEPSGD 350
TCF7L1_Takifugu_rubripes                GQHMYSIPPGGFRHPYP-ALAMN-ASMSSLVS-SRFSPHMVTPPPHGLHQTGIPHPAIVSPAIKQEPSGD 350
TCF7L1_Tetraodon_nigroviridis           GQHMYSIPPGGFRHPYP-ALAMN-ASMSSLVS-SRFSPHMVTPPPHSLHQTGIPHPAIVSPAIKQEPSGD 350
TCF7L1a_Danio_rerio                     GQHMYSIPPGGFRHPYP-ALAMN-ASMSSLVS-SRFSPHMVPHPPHGLHQTGIPHPAIVSPAIKQEPNGE 350
TCF7L1x_Tetraodon_nigroviridis          GQPMYPIA-GGFS---PAALAMN-ASMSSLMT-GGFSPRLVPT-----SRSSSPHPSAAPSVVKQEPGVS 350
TCF7L1x_Takifugu_rubripes               GQPMYPLP-GGFS---PAALAMN-ASMSSLMT-GGFSPRLVPT-----SQSSSPHPSVAPPVVKQEPGVS 350
TCF7L1x_Oryzias_latipes                 GQHMYPLT-GGFS---PAALAMN-ASMSSLVS-GSFSPRLVPT-----PQPSVSQPSVAPPAVKQEPG-E 350
Scanicula_TCF7L1                        SPPLYPIPAGSFRPSYHTALTVN-ASMS------RFSPHMAPA-PHASHATGIPHPAIVTTGIKQERLQN 350
Amblyraja_radiata_TCF7L1_partial        SPSLYSMLAPGFRPAYHTALATS-PS-----V-ARFSPQMVSP-QH-----GIPHPAIVSTGIKQEPTHS 350
Chiloscyllium_plagiosum_TCF7L1          SPPLYPIAAGSFRPSYHTALTVN-ASMS------RFSPHMAPP-PHSSHATGIPHPAITT-GIKQEQLHS 350
Elphany_shark_partial_TCF7L1            SPPMYPITAGGFRPPYP-ALAVN-TSMS------RFSPHMVPP-PHGLHPTGIPHPAIVTSNIKQEPSHS 350
TCF7L2_Homo_sapiens_V3                  GQPVYPITTGGFRHPYPTALTVN-ASMSSFLS-SRFPPHMVPP-HHTLHTTGIPHPAIVTPTVKQESSQS 350
TCF7L2_Mus_musculus_V3                  GQPVYPITTGGFRHPYPTALTVN-ASMSSFLS-SRFPPHMVPP-HHTLHTTGIPHPAIVTPTVKQESSQS 350
TCF7L2_Gallus_gallus_V3                 GQPVYPITTGGFRHPYPTALTVN-ASMSSFLS-SRFPPHMVPP-HHSLHTTGIPHPAIVTPTVKQESSQS 350
TCF7L2_Taeniopygia_guttata_V3           GQPVYPITTGGFRHPYPTALTVN-ASMSSFLS-SRFPPHMVPP-HHSLHTTGIPHPAIVTPTVKQESSQS 350
TCF7L2_Anolis_carolinensis_V3           GQPVYPITTGGFRHPYPTALTVN-ASMSSFLS-SRFPPHMVPP-HHSLHTTGIPHPAIVTPTVKQESSQN 350
TCF7L2_Chrysemys_picta_bellii_V3        GQPVYPITTGGFRHPYPTALTVN-ASMSSFLS-SRFPPHMVPP-HHSLHTTGIPHPAIVTPTVKQESSQS 350
TCF7L2_Xenopus_tropicalis_V3            GQPVYPITTGGFRHPYPTALTVN-ASMSSFLS-SRFPPHMVPP-HHSLHTTGIPHPAIVTPTVKQESSQS 350
TCF7L2L_Xenopus_laevis_V3               GQPVYPITTGGFRHPYPTALTVN-ASMSSFLS-SRFPPHMVPP-HHSLHTTGIPHPAIVNPTVKQESSQS 350
TCF7L2S_Xenopus_laevis_V3               GQPVYPITTGGFRHPYPTALTVN-ASMSSFLS-SRFPPHMVPP-HHSLHTTGIPHPAIVAPTVKQESSHS 350
TCF7L2_chr7_Acipenser_ruthenus_V3       GQPVYPITTGGFRHPYPTALTVN-ASMSSFLN-SRFPPHMVPP-HHSLHTTGIPHPAIVTHSVKQESSQS 350
TCF7L2_chr13_Acipenser_ruthenus_V3      GQPVYPITTGGFRHPYPTALTVN-ASMSSFLN-SRFPPHMVPP-HHSLHTTGIPHPAIVTHSVKQESSQS 350
TCF7L2_Latimeria_chalumnae_V3           GQPVYPITTGGFRHPYPTALTVN-ASMSSFLS-SRFPPHMVPP-HHSLHTTGIPHPAIVTPTVKQESSQN 350
TCF7L2_Lepisosteus_oculatus_V3          GQPVYPITTGGFRHPYPTALTVN-ASMSSFLS-SRFPPHMVPP-HHSLHTTGIPHPAIVTPSVKQESSQS 350
TCF7L2_Danio_rerio_V3                   GQPVYPITTGGFRHPYPTALTVN-ASMSSLLS-SRFPPHMVPP-HHSLHTTGIPHPAIVTPNVKQESSHS 350
TCF7L2_Tetraodon_nigroviridis_V3        GQPVYPITTGGFRHPYPTALTVN-ASMSSLLS-SRFPPHMVPP-HHSLHTTGIPHPAIVTPNVKQESTHS 350
TCF7L2_Oryzias_latipes_V3               GQPVYPITTGGFRHPYPTALTVN-ASMSSLLS-SRFPPHMVPP-HHSLHTTGIPHPAIVTPNVKQESSHS 350
TCF7L2_Takifugu_rubripes_V3             GQPVYPITTGGFRHPYPTALTVN-ASMSSLLS-SRFPPHMVPP-HHSLHTTGIPHPAIVTPNVKQESTHS 350
Amblyraja_radiata_TCF7L2                GQPVYPITTGGFRHPYPTALTVN-ASMSSFLS-GRFPPHMVPP-HHSLHTTGIPHPAIVTQTVKQESSQS 350
Chiloscyllium_plagiosum_TCF7L2          GQPVYPITTGGFRHPYPTALTVN-ASMSSFLS-GRFPPHMVPP-HHSLHTTGIPHPAIVTPTVKQESSQS 350
Scanicula_TCF7L2                        GQPVYPITTGGFRHPYPTALTVN-ASMSSFLS-GRFPPHMVPP-HHSLHTTGIPHPAIVTPTVKQESSQS 350
Elephant_shark_TCF7L2                   GQPVYPITTGGFRHPYPTALTVN-ASMSSFLS-GRFPPHMVPP-HHSLHTTGIPHPAIVTPSVKQESSQS 350
Lamprey_TCF_chr41                       GQPLYPL-AGGFRPPYPTAL-VN-SSMSSFLS-GRYPPHMVHP-SHGLPPTGIPHPAIVTPPVKQE-AHS 350
Pacific_lamprey_TCF_chr43               GQPLYPL-AGGFRPPYPTAL-VN-SSMSSFLS-GRYPPHMVHP-SHGLPPTGIPHPAIVTPPVKQE-AHS 350
Lethenteron_reissneri_chr47_TCF         GQPLYPL-AGGFRPPYPTAL-VN-SSMSSFLS-GRYPPHMVHP-SHGLPPTGIPHPAIVTPPVKQE-AHS 350
Lamprey_TCF_chr8                        GQPLYPLSTAGFRPPYPTALTVS-TPVS------RFPSHLVPS-HHGLHTTGIPHPAIVAPSLKPESSHS 350
Pacific_lamprey_TCF_chr22               GQPLYPLSTAGFRPPYPTALTVS-TPVS------RFPSHLVPS-HHGLHPTGIPHPAIVAPSLKPESSHS 350
Lethenteron_reissneri_chr34_TCF         GQPLYPLSTAGFRPPYPTALTVS-TPVS------RFPSHLVPS-HHGLHPTGIPHPAIVAPSLKPESSHS 350
Lamprey_putative_TCF_chr68_partial      GQPLYPLGAAGFRHPYQTAIAIN--SISSPL--GRYSPQLVPAAAAAASTLANPRTAIVTA--------- 350
Ciona_robusta_TCF_v3                    GMLRYP---------YPFASQGG---MSA-ITP--FPLVPPH--VSGMHSTMIPHPAMALP-GHLP--GH 350
Ciona_intestinalis_TCF                  GMLRYP---------YPFASQGG---MSA-ITP--FPLVPPH--VSGMHPTMIPHPAMALP-GHLP--GH 350
Ciona_savignyi_TSA_based                GMLRYP---------YPFGPQ-A---MTG-ITP--FPMVQPHPHVSGMHPTMIPHPAMALP-GHLP--GH 350
Phallucia_fumigata_TCF                  GVFPYP---------YPFANLGS---INGVS-P--FPLVRPNPHASGMHPTVISHPGLQLS-GPMT--GQ 350
Halocynthia_roretzi_TCF                 SILRYP--------HHPFAAFVA---ASS-M-P-RFPMGA-HPHNPGMHPTMIPHPALVSN-GPLS-MGN 350
B_lanceolatum_TCF                       SQGVYPISAAGYRHPYPTAIAVNASNMP------RYPV-LQHPHHPGMPPTGIPHPAIVSPHGMLKH--- 350
B_belcheri_TCF                          SQGVYPISAAGYRHPYPTAIAVNATNMP------RYPV-LQHPHHPGMPPTGIPHPAIVSPHGMLKH--- 350
B_floridae_TCF                          SQGVYPISAAGYRHPYPTAIAVNATNMPREEPI-RYPV-LQHPHHPGMPPTGIPHPAIVSPHGMLKH--- 350
Saccoglossus_kowalevskii_TCF            GQPVYPMTTAAFRGAYPAALTVNTANMT------RFG-HSAIPHHPGMTITGIPHSAIVTPGAKQD---- 350
Ptychodera_flava_TCF_TSA                GSPVYPMTTAAFRGPYPTALTVNTANMP------RFS-HPGMPPHPGLSVTGIPHPAIVTPGAKQD---- 350
Strongylocentrotus_purpuratus_TCF       GQPFYPLTSSALRSPYPPSLAVSPASMA------RLGPSPAGP----IPVGSLPHSLMHPGSSQQD---- 350
Asterias_rubens_TCF                     GQPFYPLT-SALRSPYPA--LINASGMS------RLG-HPLPPHHHGMAVTGIPHPAIVTGTRQQD---- 350
Apostichopus_japonicus_TCF              SQAFY----SSIRSPYSTNLLVNPSGIS------RLAPSSNPSSFRGIPLS--PQ----TPASQHD---- 350
                                                                                              .:
```

```
LEF1_Homo_sapiens_V2                D-SDLMHVKPQHE---QRKEQEP-KR-PHIKKPLNAFMLYMKEMRANVVAECTLKESAAINQILGRRWHA 420
LEF1_Mus_musculus_V2                D-SDLMHVKPQHE---QRKEQEP-KR-PHIKKPLNAFMLYMKEMRANVVAECTLKESAAINQILGRRWHA 420
LEF1_Gallus_gallus_V2               D-SELMHVKPQHE---QRKEQEP-KR-PHIKKPLNAFMLYMKEMRANVVAECTLKESAAINQILGRRWHA 420
LEF1_Taeniopygia_guttata_V2         D-SELMHVKPQHE---QRKEQEP-KR-PHIKKPLNAFMLYMKEMRANVVAECTLKESAAINQILGRRWHA 420
LEF1_Anolis_carolinensis_V2         D-SDLMHVKPQHE---QRKEQEP-KR-PHIKKPLNAFMLYMKEMRANVVAECTLKESAAINQILGRRWHA 420
LEF1_Chrysemys_picta_bellii_V2      D-SDLMHVKPQHE---QRKEQEP-KR-PHIKKPLNAFMLYMKEMRANVVAECTLKESAAINQILGRRWHA 420
LEF1_Xenopus_tropicalis_V2          D-NDLMHMKPHHE---QRKEQEP-KR-PHIKKPLNAFMLYMKEMRANVVAECTLKESAAINQILGRRWHA 420
LEF1L_Xenopus_laevis_V2             D-HDLMHMKPHHE---QRKEQEP-KR-PHIKKPLNAFMLYMKEMRANVVAECTLKESAAINQILGRRWHA 420
LEF1S_Xenopus_laevis_V2             D-NDLMHMKPHHE---QRKEQEP-KR-PHIKKPLNAFMLYMKEMRANVVAECTLKESAAINQILGRRWHA 420
LEF1_chr1_Acipenser_ruthenus_V2     D-NELMHMKPQHE---QRKEQEP-KR-PHIKKPLNAFMLYMKEMRANVVAECTLKESAAINQILGRRWHA 420
LEF1_chr2_Acipenser_ruthenus_V2     D-TDLMHMKPQHE---QRKEQEP-KR-PHIKKPLNAFMLYMKEMRANVVAECTLKESAAINQILGRRWHA 420
LEF1_Latimeria_chalumnae_V2         E-SDLVHMKPQHE---QRKEQEP-KR-PHIKKPLNAFMLYMKEMRANVVAECTLKESAAINQILGRRWHA 420
LEF1_Lepisosteus_oculatus_V2        D-SDLMHMKPQHE---QRKEQEP-KR-PHIKKPLNAFMLYMKEMRANVVAECTLKESAAINQILGRRWHA 420
LEF1_Danio_rerio_V2                 D-TDLMHMKPQHE---QRKEQEP-KR-PHIKKPLNAFMLYMKEMRANVVAECTLKESAAINQILGRRWHA 420
LEF1_Oryzias_latipes_V2             E-TDIMHMKPQHE---QRKEQEP-KK-PHIKKPLNAFMLYMKEMRANVVAECTLKESAAINQILGRRWHA 420
LEF1_Tetraodon_nigroviridis_V2      E-TDIMHMKPQHE---QRKDQEP-KK-PHIKKPLNAFMLYMKEMRANVVAECTLKESAAINQILGRRWHA 420
LEF1_Takifugu_rubripes_V2           E-TDIMHMKPQHE---QRKDQEP-KK-PHIKKPLNAFMLYMKEMRANVVAECTLKESAAINQILGRRWHA 420
Amblyraja_radiata_LEF1              E-IELMNTKPQHE---TRKEQEA-KK-PHIKKPLNAFMLYMKEMRANVVAECTLKESAAINQILGRRWHA 420
Chiloscyllium_plagiosum_LEF1        E-IEHMDMKPHHE---TRKEQEA-KK-PHIKKPLNAFMLYMKEMRANVVAECTLKESAAINQILGRRWHA 420
Scanicula_LEF1                      E-IEHMDIKPHQE---MRKEQEA-KK-PHIKKPLNAFMLYMKEMRANVVAECTLKESAAINQILGRRWHA 420
Elphany_shark_partial_LEF1          E-SELMSMKPHHE---SRKEQEA-KK-PHIKKPLNAFMLYMKEMRANVVAECTLKESAAINQILGRRWHA 420
TCF7_Homo_sapiens_V3                D-------KTQAE---SKAEKEA-KK-PTIKKPLNAFMLYMKEMRAKVIAECTLKESAAINQILGRRWHA 420
TCF7_Mus_musculus_V3                D-------KTQAE---PKAEKEA-KK-PVIKKPLNAFMLYMKEMRAKVIAECTLKESAAINQILGRRWHA 420
TCF7_Gallus_gallus_V3               D--RNM--KPQPE---PKREKEA-KK-PTIKKPLNAFMLYMKEMRAKVIAECTLKESAAINQILGRRWHA 420
TCF7_Taeniopygia_guttata_V3         D--RNM--KPQPE---PKREKEA-KK-PTIKKPLNAFMLYMKEMRAKVIAECTLKESAAINQILGRRWHA 420
TCF7_Anolis_carolinensis_V3         D--RSM--KSQPE---PKREKEA-KK-PTIKKPLNAFMLYMKEMRAKVIAECTLKESAAINQILGRRWHA 420
TCF7_Chrysemys_picta_bellii_V3      D--RTM--KPQPE---PKREKEA-KK-PTIKKPLNAFMLYMKEMRAKVIAECTLKESAAINQILGRRWHA 420
TCF7_Xenopus_tropicalis_V3          E--RNM--KPHSE---PKREREP-KK-PTIKKPLNAFMLYMKEMRAKVIAECTLKESAAINQILGRRWHA 420
TCF7S_Xenopus_laevis_V3             E--RNM--KQHSE---PKREKEP-KK-PAIKKPLNAFMLYMKEMRANVIAECTLKESAAINQILGRRWHA 420
TCF7_chr15_Acipenser_ruthenus_V3    D--RNMYVKQHQE---PKREKEP-KK-PVIKKPLNAFMLYMKEMRAKVIAECTLKESAAINQILGRRWHA 420
TCF7_chr29_Acipenser_ruthenus_V3    D--RNMYVKQHQE---PKREKEP-KK-PVIKKPLNAFMLYMKEMRAKVIAECTLKESAAINQILGRRWHA 420
TCF7_Latimeria_chalumnae_V3         D--RNM--KPQPE---PKREKEP-KK-PTIKKPLNAFMLYMKEMRAKVIAECTLKESAAINQILGRRWHA 420
TCF7_Lepisosteus_oculatus_V3        D--RNMYAKPQQE---PKRDKEP-KK-PVIKKPLNAFMLYMKEMRAKVIAECTLKESAAINQILGRRWHA 420
TCF7_Danio_rerio_V3                 D--RSIYNKSHAE---AKREKEP-KK-PVIKKPLNAFMLYMKEMRAKVIAECTLKESAAINQILGRRWHA 420
TCF7_Oryzias_latipes_V3             D--RGMYVKQQVE---TKREKEP-KK-PVIKKPLNAFMLYMKEMRAKVIAECTLKESAAINQILGRRWHA 420
TCF7_Takifugu_rubripes_V3           D--RGMYLKQEV-----KQQKEP-KK-PVIKKPLNAFMLYMKEMRAKVIAECTLKESAAINQILGRRWHA 420
TCF7_Tetraodon_nigroviridis_V3      D--RGMYLKQEV-----KQQKEP-KK-PVIKKPLNAFMLYMKEMRAKVIAECTLKESAAINQILGRRWHA 420
Amblyraja_radiata_TCF7              DGLRCM--KSNPE---PKREKES-KK-PTIKKPLNAFMLYMKEMRAKVVAECTLKESAAINQILGRRWHA 420
Chiloscyllium_plagiosum_TCF7        DGLRSM--KPNPE---PKREKES-KK-PTIKKPLNAFMLYMKEMRAKVVAECTLKESAAINQILGRRWHA 420
Scanicula_TCF7                      DGLRSM--KPNPE---PKREKES-KK-PTIKKPLNAFMLYMKEMRAKVVAECTLKESAAINQILGRRWHA 420
Elphany_shark_partial_TCF7          DGLRCM--KPNPE---PKREKES-KK-PAIKKPLNAFMLYMKEMRAKVVAECTLKESAAINQILGRRWHA 420
TCF7L1_Homo_sapiens                 SLSPAVSVK-SPV---TVKKEEE-KK-PHVKKPLNAFMLYMKEMRAKVVAECTLKESAAINQILGRKWHN 420
TCF7L1_Mus_musculus                 SLSPAVSAK-SPV---TVKKEEE-KK-PHVKKPLNAFMLYMKEMRAKVVAECTLKESAAINQILGRKWHN 420
TCF7L1_Gallus_gallus                STSPGVSSK-SPI---TVKKEEE-KK-PHIKKPLNAFMLYMKEMRAKVVAECTLKESAAINQILGRRWHS 420
TCF7L1_Taeniopygia_guttata          SSSPGGSSK-SPA---AVKKEEE-KK-PHIKKPLNAFMLYMKEMRAKVVAECTLKESAAINQILGRRWHS 420
TCF7L1_Anolis_carolinensis          SMSPEGSSK-TPV---IVKKEEE-KK-PHIKKPLNAFMLYMKEMRAKVVAECTLKESAAINQILGRRWHS 420
TCF7L1_Chrysemys_picta_bellii       NMSPGGNSK-SPG---SVKKEEE-KK-PHIKKPLNAFMLYMKEMRAKVVAECTLKESAAINQILGRRWHS 420
TCF7L1_Xenopus_tropicales           NISPNLITK-PSV---VVKKEEE-KK-PHIKKPLNAFMLYMKEMRAKVVAECTLKESAAINQILGRRWHS 420
TCF7L1L_Xenopus_laevis              NISPNLHTK-SNM---IVKKEEE-KK-PHIKKPLNAFMLYMKEMRAKVVAECTLKESAAINQILGRRWHS 420
TCF7L1S_Xenopus_laevis              NISPNLSTK-SNV---VVKKEEE-KK-PHIKKPLNAFMLYMKEMRAKVVAECTLKESAAINQILGRRWHS 420
TCF7L1_chr41_Acipenser_ruthenus     HLSPSMHSK-SPA---PVKKEEE-KK-PHVKKPLNAFMLYMKEMRAKVVAECTLKESAAINQILGRRWHS 420
TCF7L1_ste150_Acipenser_ruthenus    HLSPSMHSK-SPA---PVKKEEE-KK-PHVKKPLNAFMLYMKEMRAKVVAECTLKESAAINQILGRRWHS 420
TCF7L1_Latimeria_chalumnae          TISPNVHPK-SPV---SLKKEEE-KK-PHIKKPLNAFMLYMKEMRAKVVAECTLKESAAINQILGRRWHS 420
TCF7L1a_Lepisosteus_oculatus        TVSPTAHSK-SPG---PVKKEED-KK-PHIKKPLNAFMLYMKEMRAKVVAECTLKESAAINQILGRRWHS 420
TCF7L1b_Danio_rerio                 -LSPPVNTK-SPG---PNKKDED-KK-PHIKKPLNAFMLYMKEMRAKVVAECTLKESAAINQILGRRWHS 420
TCF7L1_Oryzias_latipes              NISPSMHAK-SPV---PAKKEED-KK-PHIKKPLNAFMLYMKEMRAKVVAECTLKESAAINQILGRRWHS 420
TCF7L1_Takifugu_rubripes            NISPTMQAK-SPA---PHKKEED-KK-PHIKKPLNAFMLYMKEMRAKVVAECTLKESAAINQILGRRWHS 420
TCF7L1_Tetraodon_nigroviridis       NISPTMQAK-SPA---PHKKEED-KK-PHIKKPLNAFMLYMKEMRAKVVAECTLKESAAINQILGRRWHS 420
TCF7L1a_Danio_rerio                 SPSNSTHGK-PSV---PVKKEEE-KK-PHIKKPLNAFMLYMKEMRAKVVAECTLKESAAINQILGRRWHS 420
TCF7L1x_Tetraodon_nigroviridis      NSSPP-GAK-MAES--QLEKEED-KR-FYIKKPLNAFMLYMREERPKVVAQCNVKESATINQILGQRWHS 420
TCF7L1x_Takifugu_rubripes           NSSQP-GTK-MPEG--QHEKEDD-KR-FYIKKPLNAFMLYMREERPKVVAQCNVKESATINQILGQRWHS 420
TCF7L1x_Oryzias_latipes             HSGHE-HSK-SASG--A-PREDD-RR-PHIKKPLNAFMLYMRDERPKVVSQCKVKESATINQILGQRWHS 420
Scanicula_TCF7L1                    QPSPNDTSK-PLV---V-KE-DE-KRKPHIKKPLNAFMLYMKEMRAKVVAECTLKESAAINQILGRKWHA 420
Amblyraja_radiata_TCF7L1_partial    QPSPSHVSRPPSI-----KEEDE-KRKPHIKKPLNAFMLYMKEMRAKVVAECTLKESAAINQILGRKWHG 420
Chiloscyllium_plagiosum_TCF7L1      QPSPSETPK-PLM---VKKE-DE-KRKPHIKKPLNAFMLYMKEMRAKVVAECTLKESAAINQILGRKWHA 420
Elphany_shark_partial_TCF7L1        HIAGCNGYRRPLS---IKKEEEK-KR-PHIKKPLNAFMLYMKEMRAKVVAECTLKESAAINQILGRRWHA 420
TCF7L2_Homo_sapiens_V3              DVGSLHSSK-HQD---SKKEEEK-KK-PHIKKPLNAFMLYMKEMRAKVVAECTLKESAAINQILGRRWHA 420
TCF7L2_Mus_musculus_V3              DVGSLHSSK-HQD---SKKEEEK-KK-PHIKKPLNAFMLYMKEMRAKVVAECTLKESAAINQILGRRWHA 420
TCF7L2_Gallus_gallus_V3             DVGSLHSSK-HQD---SKKEEEK-KK-PHIKKPLNAFMLYMKEMRAKVVAECTLKESAAINQILGRRWHA 420
TCF7L2_Taeniopygia_guttata_V3       DVGSLHSSK-HQD---SKKEEEK-KK-PHIKKPLNAFMLYMKEMRAKVVAECTLKESAAINQILGRRWHA 420
TCF7L2_Anolis_carolinensis_V3       DVGSLHSSK-HQD---SKKEEEK-KK-PHIKKPLNAFMLYMKEMRAKVVAECTLKESAAINQILGRRWHA 420
TCF7L2_Chrysemys_picta_bellii_V3    DVGSLHSSK-HQD---SKKEEEK-KK-PHIKKPLNAFMLYMKEMRAKVVAECTLKESAAINQILGRRWHA 420
TCF7L2_Xenopus_tropicalis_V3        DMGSLHSSK-HQD---SKKEEEK-KK-PHIKKPLNAFMLYMKEMRAKVVAECTLKESAAINQILGRRWHA 420
TCF7L2L_Xenopus_laevis_V3           DMGSLHSSK-HQD---SKKEEEK-KK-PHIKKPLNAFMLYMKEMRAKVVAECTLKESAAINQILGRRWHA 420
TCF7L2S_Xenopus_laevis_V3           DMGSLHSSK-HQD---SKKEEEK-KK-PHIKKPLNAFMLYMKEMRAKVVAECTLKESAAINQILGRRWHA 420
TCF7L2_chr7_Acipenser_ruthenus_V3   DIGSLNSSK-HQD---QKKEDEK-KKQPHIKKPLNAFMLYMKEMRAKVVAECTLKESAAINQILGRRWHA 420
TCF7L2_chr13_Acipenser_ruthenus_V3  DIGSLNSSK-HQD---QKKEDEK-KKQPHIKKPLNAFMLYMKEMRAKVVAECTLKESAAINQILGRRWHA 420
```

```
TCF7L2_Latimeria_chalumnae_V3           DVGSLHSSK-HQD---SKKEEEK-KK-PHIKKPLNAFMLYMKEMRAKVVAECTLKESAAINQILGRRWHA 420
TCF7L2_Lepisosteus_oculatus_V3          DIGSLNSSK-HQD---SKKEEEK-KKQPHIKKPLNAFMLYMKEMRAKVVAECTLKESAAINQILGRRWHA 420
TCF7L2_Danio_rerio_V3                   DIGSLNSSK-HQD---AKKEEEK-KKQPHIKKPLNAFMLYMKEMRAKVVAECTLKESAAINQILGRRWHA 420
TCF7L2_Tetraodon_nigroviridis_V3        DINSLNSSK-QSD---SKKEPEK-KKEVHIKKPLNAFMLYMKEMRAKVVAECTLKESAAINQILGRRWHA 420
TCF7L2_Oryzias_latipes_V3               DISSLNSSK-QSD---AKKEEEK-KKQVHIKKPLNAFMLYMKEMRAKVVAECTLKESAAINQILGRRWHA 420
TCF7L2_Takifugu_rubripes_V3             DINSLNSSK-QSD---SKKEPEK-KKEVHIKKPLNAFMLYMKEMRAKVVAECTLKESAAINQILGRRWHA 420
Amblyraja_radiata_TCF7L2                DIGSLNNSKHHQD---AKKEEEK-KK-PHIKKPLNAFMLYMKEMRAKVVAECTLKESAAINQILGRRWHA 420
Chiloscyllium_plagiosum_TCF7L2          DIGSLNNSKHHQD---AKKEEEK-KK-PHIKKPLNAFMLYMKEMRAKVVAECTLKESAAINQILGRRWHA 420
Scanicula_TCF7L2                        DIGSLNNSKHHQD---AKKEEEK-KK-PHIKKPLNAFMLYMKEMRAKVVAECTLKESAAINQILGRRWHA 420
Elephant_shark_TCF7L2                   DIGSLNNSKHHQD---AKKEEEK-KK-PHIKKPLNAFMLYMKEMRAKVVAECTLKESAAINQILGRRWHA 420
Lamprey_TCF_chr41                       GANGSEHQK--QN---SKKEAEE-KK-PHIKKPLNAFMLFMKEMRAKVIAECTLKESAAINQILGRRWHA 420
Pacific_lamprey_TCF_chr43               GANGSEHQK--QN---SKKEAEE-KK-PHIKKPLNAFMLFMKEMRAKVIAECTLKESAAINQILGRRWHA 420
Lethenteron_reissneri_chr47_TCF         GANGSEHQK--QN---SKKEAEE-KK-PHIKKPLNAFMLFMKEMRAKVIAECTLKESAAINQILGRRWHA 420
Lamprey_TCF_chr8                        NPQPSQHPQNKAA---EEKRDAD-KK-PHIKKPLNAFMLYMKEMRAKVVAECTLKESAAINQILGRRWHA 420
Pacific_lamprey_TCF_chr22               NPQPSQHPQNKAA---EEKRDAD-KK-PHIKKPLNAFMLYMKEMRAKVVAECTLKESAAINQILGRRWHA 420
Lethenteron_reissneri_chr34_TCF         NPQPSQHPQNKAA---EEKRDAD-KK-PHIKKPLNAFMLYMKEMRAKVVAECTLKESAAINQILGRRWHA 420
Lamprey_putative_TCF_chr68_partial      -------GDG-QR--PPQQQPP-HQR-CHVKKPLNAFMLYMKETRGRVIAECTLKESAAINQILGRRWHA 420
Ciona_robusta_TCF_v3                    N-QSKGGVD-----KKGPEPGS-KKRRPYVKKPLNAFMLYMKEQRAKVVAECTLKESAAINQILGRKWHS 420
Ciona_intestinalis_TCF                  N-QSKGGVD-----KKGPEPGS-KKRRPYVKKPLNAFMLYMKEQRAKVVAECTLKESAAINQILGRKWHS 420
Ciona_savignyi_TSA_based                A-TSKSNPD-----KKGHEGGP-KKRRPYVKKPLNAFMLYMKEQRAKVVAECTLKESAAINQILGRKWHS 420
Phallucia_fumigata_TCF                  G-PGKSMSD-----KKNSDSTS-KRRRPYVKKPLNAFMLYMKEQRAKVVAECTLKESAAINQILGRKWHS 420
Halocynthia_roretzi_TCF                 VHPAHNKMD--QDKPSKSEHSG-HGHRPYVKKPLNAFMLYMKEQRAKVVAECTLKESAAINQILGRKWHS 420
B_lanceolatum_TCF                       -DPSHMHQK--QDQKKEPE----KKK-PHIKKPLNAFMLYMKEMRAKVVAECTLKESAAINQILGRRWHA 420
B_belcheri_TCF                          -DPSQMHQK--QDQKKEPE----KKK-PHIKKPLNAFMLYMKEMRAKVVAECTLKESAAINQILGRRWHA 420
B_floridae_TCF                          -DPSQMHQK--QDQKKEPE----KKK-PHIKKPLNAFMLYMKEMRAKVVAECTLKESAAINQILGRRWHA 420
Saccoglossus_kowalevskii_TCF            -----LQQQ-NNDREVLISPYRQKSETPHIKKPLNAFMLYMKEMRAKVVAECTLKESAAINQILGRRWHA 420
Ptychodera_flava_TCF_TSA                -----LQQQ-SNDR--------QKGK-PHVKKPLNAFMLYMKEMRAKVVAECTLKESAAINQILGRRWHA 420
Strongylocentrotus_purpuratus_TCF       -----AQQQ-SNDRHQ------EKEKQQHIKKPLNAFMLYMKEMRASVVKECTLKESAAINQILGRRWHA 420
Asterias_rubens_TCF                     -----TLQQ-SNDRQP------PKEEHPHIKKPLNAFMLYMKEKRASVVKECTLKESAAINQILGRRWHA 420
Apostichopus_japonicus_TCF              -----TQQQ-SNDREK------TDKKKVHIKKPLNAFMLYMKEMRASVVKECTLKESAAINQILGRRWHA 420
                                                                 .   :*********:*:: *  *: :*.:****:******::**

LEF1_Homo_sapiens_V2                    LSREEQAKYYELARKERQLHMQLYPGWSARDNYGKKKKRKREK----LQ-ESA----------------- 490
LEF1_Mus_musculus_V2                    LSREEQAKYYELARKERQLHMQLYPGWSARDNYGKKKKRKREK----LQ-EST----------------- 490
LEF1_Gallus_gallus_V2                   LSREEQAKYYELARKERQLHMQLYPGWSARDNYGKKKKRKREK----LQ-ESA----------------- 490
LEF1_Taeniopygia_guttata_V2             LSREEQAKYYELARKERQLHMQLYPGWSARDNYGKKKKRKREK----LQ-ESA----------------- 490
LEF1_Anolis_carolinensis_V2             LSREEQAKYYELARKERQLHMQLYPGWSARDNYGKKKKRKREK----LQ-EST----------------- 490
LEF1_Chrysemys_picta_bellii_V2          LSREEQAKYYELARKERQLHMQLYPGWSARDNYGKKKKRKREK----LQ-EST----------------- 490
LEF1_Xenopus_tropicalis_V2              LSREEQAKYYELARKERQLHMQLYPGWSARDNYGKKKKRKREK----LQ-EST----------------- 490
LEF1L_Xenopus_laevis_V2                 LSREDQAKYYELARKERQLHMQLYPGWSARDNYGKKKKRKREK----LQ-EST----------------- 490
LEF1S_Xenopus_laevis_V2                 LSREEQSKYYELARKERQLHMQLYPGWSARDNYGKKKKRKREK----LQ-EST----------------- 490
LEF1_chr1_Acipenser_ruthenus_V2         LSREEQAKYYELARKERQLHMQLYPGWSARDNYGKKKKRKREK----IQ-ETT----------------- 490
LEF1_chr2_Acipenser_ruthenus_V2         LSREEQAKYYELARKERQLHMQLYPGWSARDNYGKKKKRKREK----IQ-ETT----------------- 490
LEF1_Latimeria_chalumnae_V2             LSREEQAKYYELARKERQLHMQLYPGWSARDNYGKKKKRKREK----LQ-EST----------------- 490
LEF1_Lepisosteus_oculatus_V2            LSREEQAKYYELARKERQLHMQLYPGWSARDNYGKKKKRKREK----LQ-EPA----------------- 490
LEF1_Danio_rerio_V2                     LSREEQAKYYELARKERQLHMQLYPGWSARDNYGKKKKRKREK----IQ-EPA----------------- 490
LEF1_Oryzias_latipes_V2                 LSREEQAKYYELARKERQLHMQLYPGWSARDNYGKKKKRKREK----MQ-ESA----------------- 490
LEF1_Tetraodon_nigroviridis_V2          LSREEQAKYYELARKERQLHMQLYPGWSARDNYGKKKKRKREK----MQ-ETA----------------- 490
LEF1_Takifugu_rubripes_V2               LSREEQAKYYELARKERQLHMQLYPGWSARDNYGKKKKRKREK----MQ-ETA----------------- 490
Amblyraja_radiata_LEF1                  LTREEQAKYYELARKERQLHMQLYPGWSARDNYGKKKKRKREK----MQ-ETT----------------- 490
Chiloscyllium_plagiosum_LEF1            LSREEQAKYYELARKERQLHMQLYPGWSARDNYGKKKKRKREK----MQ-ETT----------------- 490
Scanicula_LEF1                          LSREEQAKYYELARKERQLHMQLYPGWSARDNYGKKKKRKREK----MQ-ETT----------------- 490
Elphany_shark_partial_LEF1              LTREEQAKYYELARKERQLHMQLYPGWSARDNYGKKKKRKREK----MQ-EAT----------------- 490
TCF7_Homo_sapiens_V3                    LSREEQAKYYELARKERQLHMQLYPGWSARDNYGKKKRRSREK-----H-QES-TTDPGSPKKCRARFGL 490
TCF7_Mus_musculus_V3                    LSREEQAKYYELARKERQLHMQLYPGWSARDNYGKKKRRSREK-----H-QES-TTDPGSPKKCRARFGL 490
TCF7_Gallus_gallus_V3                   LSREEQAKYYELARKERQLHMQLYPGWSARDNYGKKKRRTREK-----Q-QDS-NSDPASPKKCRARFGL 490
TCF7_Taeniopygia_guttata_V3             LSREEQAKYYELARKERQLHMQLYPGWSARDNYGKKKRRTREK-----Q-QDS-SSDPASPKKCRARFGL 490
TCF7_Anolis_carolinensis_V3             LSREEQAKYYELARKERQLHMQLYPGWSARDNYGKKKRRTREK-----Q-QDS-NSDPASPKKCRARFGL 490
TCF7_Chrysemys_picta_bellii_V3          LSREEQAKYYELARKERQLHMQLYPGWSARDNYGKKKRRAREK-----Q-QDS-NSDPASPKKCRARFGL 490
TCF7_Xenopus_tropicalis_V3              LSREEQSKYYELARKERQLHMQLYPGWSARDNYGKKKRRTREK-----H-QDS-SSDPGSPKKCRARFGL 490
TCF7S_Xenopus_laevis_V3                 LSREEQSKYYELARKERQLHMQLYPGWSARDNYGKRKRRTREK-----H-QDS-SSDPGSPKKCRARFGL 490
TCF7_chr15_Acipenser_ruthenus_V3        LTREEQAKYYELARKERQLHMQLYPSWSARDNYGKKKRRKREK-----Q-PDS-NLDPGSPKKCRARFGL 490
TCF7_chr29_Acipenser_ruthenus_V3        LTREEQAKYYELARKERQLHMQLYPSWSARDNYGKKKRRKREK-----Q-PDS-NLDPGSPKKCRARFGL 490
TCF7_Latimeria_chalumnae_V3             LSREEQAKYYELARKERQLHMQLYPGWSARDNYGKKKRRTREK-----Q-HDN-SSDPGSPKKCRARFGL 490
TCF7_Lepisosteus_oculatus_V3            LSREEQAKYYELARKERQLHMQLYPSWSARDNYGKKKRRKREK-----Q-QDS-SSDPGSPKKCRARFGL 490
TCF7_Danio_rerio_V3                     LTREEQAKYYELARKERQLHMQLYPSWSARDNYGKKKRRKRDK-----Q-QDS-STEPGSPKKCRARFGL 490
TCF7_Oryzias_latipes_V3                 LTREEQAKYYELARKERQLHMQLYPTWSARDNYGKRKKRKREK-----Q-QDS-NTDPGSPKKCRARFGL 490
TCF7_Takifugu_rubripes_V3               LTREEQAKYYELARKERQLHMQLYPTWSARDNYGKRKKRKREK-----L-QDS-GADPGSPKKCRARFGL 490
TCF7_Tetraodon_nigroviridis_V3          LTREEQAKYYELARKERQLHMQLYPTWSARDNYGKRKKRKREK-----L-QDS-GADPGSPKKCRARFGL 490
Amblyraja_radiata_TCF7                  LTREEQAKYYELARKERQLHMQLYPGWSARDNYGKKKRRKRDK-----Q-QDS-DQDPSSPKKCRARFGL 490
Chiloscyllium_plagiosum_TCF7            LSREEQAKYYELARKERQLHMQLYPGWSARDNYGKKKRRKREK-----Q-QDS-DPDPSSPKKCRARFGL 490
Scanicula_TCF7                          LSREEQAKYYELARKERQLHMQLYPGWSARDNYGKKKRRKREK-----Q-QDS-DPDPSSPKKCRARFGL 490
Elphany_shark_partial_TCF7              LTREEQAKYYELARKERQLHMQLYPGWSARDNYGKKKRRKREK-----Q-QDS-DGDPSSPKKCRALFGL 490
TCF7L1_Homo_sapiens                     LSREEQAKYYELARKERQLHSQLYPTWSARDNYGKKKKRKREKQSQQVQ-EAE----------------- 490
TCF7L1_Mus_musculus                     LSREEQAKYYELARKERQLHAQLYPTWSARDNYGKKKKRKREKQSQQIQ-EAE----------------- 490
TCF7L1_Gallus_gallus                    LSREEQAKYYELARKERQLHSQLYPTWSARDNYGKKKKRKREKLAQQSH-EAE----------------- 490
TCF7L1_Taeniopygia_guttata              LSREEQAKYYELARKERQLHSQLYPTWSARDNYGKKKKRKREKLAQQSQ-DAD----------------- 490
TCF7L1_Anolis_carolinensis              LSREEQAKYYELARKERQLHSQLYPTWSARDNYGKKKKRKREKQPQQIQ-ETG----------------- 490
```

```
TCF7L1_Chrysemys_picta_bellii              LSREEQAKYYELARKERQLHSQLYPTWSARDNYGKKKKRKREKQPQQIQ-DTE----------------- 490
TCF7L1_Xenopus_tropicales                  LSREEQAKYYELARKERQLHSQLYPTWSARDNYGKRKKRKRDK---QSP-EME----------------- 490
TCF7L1L_Xenopus_laevis                     LSREEQAKYYELARKERQLHSQLYPSWSARDNYGKRKKRKREK---QSP-EME----------------- 490
TCF7L1S_Xenopus_laevis                     LSREEQAKYYELARKERQLHSQLYPSWSARDNYGKKKKRKREK---QSP-EME----------------- 490
TCF7L1_chr41_Acipenser_ruthenus            LTREEQAKYYELARKERQLHSQLYPGWSARDNYGKRKKRKREK---QQP-DGE----------------- 490
TCF7L1_ste150_Acipenser_ruthenus           LTREEQAKYYELARKERQLHSQLYPGWSARDNYGKRKKRKREK---QQP-DGE----------------- 490
TCF7L1_Latimeria_chalumnae                 LTREEQAKYYELARKERQLHSQLYPTWSARDNYGKKKKRKREK-QQASD-ADM----------------- 490
TCF7L1a_Lepisosteus_oculatus               LSREEQAKYYELARKERQLHSQLYPGWSARDNYGKRKKRKRESKQETTA-ED------------------ 490
TCF7L1b_Danio_rerio                        LSREEQAKYYELARKERQLHSQLYPGWSARDNYGKRKKRKRDNKTDSTP-ED------------------ 490
TCF7L1_Oryzias_latipes                     LSREEQAKYYELARKERQLHSQLYPGWSARDNYGKRKKRKRENKPDSKP-ED------------------ 490
TCF7L1_Takifugu_rubripes                   LSREEQAKYYELARKERQLHSQLYPGWSARDNYGKRKKRKRENKPDSKP-ED------------------ 490
TCF7L1_Tetraodon_nigroviridis              LSREEQAKYYELARKERQLHSQLYPGWSARDNYGKRKKRKRENKPDSKP-ED------------------ 490
TCF7L1a_Danio_rerio                        LSREEQAKYYELARKERQLHSQLYPGWSARDNYGKRKKRKRDCKSDSPS-ES------------------ 490
TCF7L1x_Tetraodon_nigroviridis             LTKEEQAKYYEMARKERLVHSKLYPGWSARENYGKKKKRKR-----SKS-ESN----------------- 490
TCF7L1x_Takifugu_rubripes                  LTKEEQAKYYEMARKERLVHSKLYPGWSARENYGKKKKRKR-----SKS-ESN-----EEGVPPQLKRPP 490
TCF7L1x_Oryzias_latipes                    LSKDQQAKYYELARKERLLHSKLYPNWSARDNYGKKKKRKR-----IKS-ESQ-----LEADASDGFLPQ 490
Scanicula_TCF7L1                           LTREEQSKYYELARKERQLHSQLYPGWSARDNYGKRKKRKRDK---QQT-ETD----------------- 490
Amblyraja_radiata_TCF7L1_partial           LTREQQSKYYELARMERQLHSQLYPGWSARDNYGKRKKRKR---GQQMSEESN----------------- 490
Chiloscyllium_plagiosum_TCF7L1             LTREEQSKYYELARKERQLHSQLYPGWSARDNYGKRKKRKRDK---QQP-ERD----------------- 490
Elphany_shark_partial_TCF7L1               LSREEQAKYYELARKERQLHSQLYPGWSARDNYGKKKKRKREK----QQRE------------------- 490
TCF7L2_Homo_sapiens_V3                     LSREEQAKYYELARKERQLHMQLYPGWSARDNYGKKKKRKRDK----QPGE---TNDLSAPKKCRARFGL 490
TCF7L2_Mus_musculus_V3                     LSREEQAKYYELARKERQLHMQLYPGWSARDNYGKKKKRKRDK----QPGE---TNDLSAPKKCRARFGL 490
TCF7L2_Gallus_gallus_V3                    LSREEQAKYYELARKERQLHMQLYPGWSARDNYGKKKKRKRDK----QPGE---TNDLSAPKKCRARFGL 490
TCF7L2_Taeniopygia_guttata_V3              LSREEQAKYYELARKERQLHMQLYPGWSARDNYGKKKKRKRDK----QPGE---TNDLSAPKKCRARFGL 490
TCF7L2_Anolis_carolinensis_V3              LSREEQAKYYELARKERQLHMQLYPGWSARDNYGKKKKRKRDK----QPGE---TNDLSAPKKCRARFGL 490
TCF7L2_Chrysemys_picta_bellii_V3           LSREEQAKYYELARKERQLHMQLYPGWSARDNYGKKKKRKRDK----QPGE---TNDLSAPKKCRARFGL 490
TCF7L2_Xenopus_tropicalis_V3               LSREEQAKYYELARKERQLHMQLYPGWSARDNYGKKKKRKREK----QQGE---ANDLSAPKKCRARFGL 490
TCF7L2L_Xenopus_laevis_V3                  LSREEQAKYYELARKERQLHMQLYPGWSARDNYGKKKKRKREK----QQGE---ANDLSAPKKCRARFGL 490
TCF7L2S_Xenopus_laevis_V3                  LSREEQAKYYELARKERQLHMQLYPGWSARDNYGKKKKRKREK----QQGE---ANDLSAPKKCRARFGL 490
TCF7L2_chr7_Acipenser_ruthenus_V3          LTREEQAKYYELARKERQLHMQLYPGWSARDNYGKKKKRKRDK----QQGE---ANDLSAPKKCRARFGL 490
TCF7L2_chr13_Acipenser_ruthenus_V3         LTREEQAKYYELARKERQLHMQLYPGWSARDNYGKKKKRKRDK----QQGE---ANDLSAPKKCRARFGL 490
TCF7L2_Latimeria_chalumnae_V3              LSREEQAKYYELARKERQLHMQLYPGWSARDNYGKKKKRKRDK----QPGE---TNDLSAPKKCRARFGL 490
TCF7L2_Lepisosteus_oculatus_V3             LSREEQAKYYELARKERQLHMQLYPGWSARDNYGKKKKRKRDK----QPGE---TNDLSAPKKCRARFGL 490
TCF7L2_Danio_rerio_V3                      LSREEQAKYYELARKERQLHMQLYPGWSARDNYGKKKKRKREK----QAGE---GNDLSAPKKCRARFGL 490
TCF7L2_Tetraodon_nigroviridis_V3           LSREEQAKYYELARKERQLHMQLYPGWSARDNYGKRKKRKREK---QQQAE---GNDLSAPKKCRARFGL 490
TCF7L2_Oryzias_latipes_V3                  LSREEQAKYYELARKERQLHMQLYPGWSARDNYGKRKKRKREK----QQAE---SNDLSAPKKCRARFGL 490
TCF7L2_Takifugu_rubripes_V3                LSREEQAKYYELARKERQLHMQLYPGWSARDNYGKRKKRKREK---QQQAE---GNDLSAPKKCRARFGL 490
Amblyraja_radiata_TCF7L2                   LSREEQAKYYELARKERQLHMQLYPGWSARDNYGKRKKRKREK----QAGE---TNDLSAPKKCRARFGL 490
Chiloscyllium_plagiosum_TCF7L2             LSREEQAKYYELARKERQLHMQLYPGWSARDNYGKRKKRKREK----QAGE---TNDLSAPKKCRARFGL 490
Scanicula_TCF7L2                           LTREEQAKYYELARKERQLHMQLYPGWSARDNYGKRKKRKREK----QAGE---TNDANTPKKCRALFGL 490
Elephant_shark_TCF7L2                      LSREEQAKYYELARKERQLHMQLYPGWSARDNYGKRKKRKREK----QQGE---TNDLSAPKKCRARFGL 490
Lamprey_TCF_chr41                          LSREEQAKYYELARKERQLHSQLYPGWSARDNYGKKKKRKREK-----Q-PAEAVIEQESAKKCRARYGV 490
Pacific_lamprey_TCF_chr43                  LSREEQAKYYELARKERQLHSQLYPGWSARDNYGKKKKRKREK-----Q-PAEAVIEQESAKKCRARYGV 490
Lethenteron_reissneri_chr47_TCF            LSREEQAKYYELARKERQLHSQLYPGWSARDNYGKKKKRKREK-----Q-PAEAVIEQESAKKCRARYGV 490
Lamprey_TCF_chr8                           LSREEQAKYYELARKERQLHMQLYPGWSARDNYGKRKKRRREK----QQVEVSHSMELAAAKRCRAHFGL 490
Pacific_lamprey_TCF_chr22                  LSREEQAKYYELARKERQLHMQLYPGWSARDNYGKRKKRRREK----QQVEVSHSMELAAAKRCRAHFGL 490
Lethenteron_reissneri_chr34_TCF            LSREEQAKYYELARKERQLHMQLYPGWSARDNYGKRKKRRREK-----Q-QVEHSMELAAAKRCRAHFGL 490
Lamprey_putative_TCF_chr68_partial         LTREQQARYYELARKERQLHMQLYPGWSARDNYGKRKKRKRER-----Q-HGE-IADINAPKKCRARYGL 490
Ciona_robusta_TCF_v3                       LNREEQQKYYEMARKERQLHQQMFPGWSARDNYGKRKKRKKEK----TQ-D--CT-AQN-PKKCRAVFGL 490
Ciona_intestinalis_TCF                     LNREEQQKYYEMARKERQLHQQMFPGWSARDNYGKRKKRKKEK----TQ-D--CT-AQN-PKKCRAVFGL 490
Ciona_savignyi_TSA_based                   LNRDEQQKYYEMARKERQLHQQMFPGWSARDNYGKRKKRKKEK----TQ-D--CT-AQN-PKKCRAVFGL 490
Phallucia_fumigata_TCF                     LNRDEQQKYYEMARKERQIHMQMFPGWSARDNYGKRKKRKKEK----AQ-D--CT-AQN-PKKCRAVFGL 490
Halocynthia_roretzi_TCF                    LSREEQQKYYELARKERQLHLQMYPGWSARDNYGKRKKRKKEK----SQGE--CT-AQN-PKKCRAVFGL 490
B_lanceolatum_TCF                          LPREEQAKYYELARKERQLHMQLYPGWSARDNYGKKKKRKRDK----QQGD--TSEVSN-PKKCRARFGL 490
B_belcheri_TCF                             LPREEQAKYYELARKERQLHMQLYPGWSARDNYGKKKKRKRDK----QQGD--TSEVSN-PKKCRARFGL 490
B_floridae_TCF                             LPREEQAKYYELARKERQLHMQLYPGWSARDNYGKKKKRKRDK----QQGD--TSEVSN-PKKCRARFGL 490
Saccoglossus_kowalevskii_TCF               LTREEQAKYYELARKERQLHMQLYPGWSARDNYGKKKKRKRDK----SQGD--NSEASN-PKKCRARFGL 490
Ptychodera_flava_TCF_TSA                   LSREEQAKYYELARKERQLHMQLYPGWSARDNYGKKKKRKRDK----AQGD--NSEASN-PKKCRARFGL 490
Strongylocentrotus_purpuratus_TCF          LTREEQAKYYELARKERQLHMQLYPGWSARDNYGKKKKKKRDK----SHGD--NGEPST-PKKCRARFGV 490
Asterias_rubens_TCF                        LSREEQAKYYELARKERQLHMQLYPSWTARDNYGKKKKKKKDK----NPGD--NSEPST-PKKCRARFGV 490
Apostichopus_japonicus_TCF                 LSREEQAKYYELARKERQLHMQLYPGWSARDNYCRRRKKRKDK----NQGD--ASDPST-PKKCRARFGV 490
                                           * :::* :***:** ** :* :::* *:**:** ::::: :

LEF1_Homo_sapiens_V2                       ------SGGKRSSFPT---- 510
LEF1_Mus_musculus_V2                       ------SGGKRSSFPT---- 510
LEF1_Gallus_gallus_V2                      ------SGGKRNAFST---- 510
LEF1_Taeniopygia_guttata_V2                ------SGGKRNAFST---- 510
LEF1_Anolis_carolinensis_V2                ------SGGKRNAFST---- 510
LEF1_Chrysemys_picta_bellii_V2             ------AGGKRNAFST---- 510
LEF1_Xenopus_tropicalis_V2                 ------SGGKKNALST---- 510
LEF1L_Xenopus_laevis_V2                    ------SGGKKNTFST---- 510
LEF1S_Xenopus_laevis_V2                    ------SGGKKNTFSS---- 510
LEF1_chr1_Acipenser_ruthenus_V2            ------SGGKRNAFSM---- 510
LEF1_chr2_Acipenser_ruthenus_V2            -------SGGKYAFST---- 510
LEF1_Latimeria_chalumnae_V2                ------SGGKRNAFSA---- 510
LEF1_Lepisosteus_oculatus_V2               ------SGGKRNTFST---- 510
LEF1_Danio_rerio_V2                        ------SGGKRTSFSS---- 510
LEF1_Oryzias_latipes_V2                    ------TGGKRNGFST---- 510
```

```
LEF1_Tetraodon_nigroviridis_V2          ------TGGKRSNFST---- 510
LEF1_Takifugu_rubripes_V2               ------TGGKRSNFST---- 510
Amblyraja_radiata_LEF1                  -------SGKQNTFTT---- 510
Chiloscyllium_plagiosum_LEF1            -------SGKQNAFST---- 510
Scanicula_LEF1                          -------SGKQNAFST---- 510
Elphany_shark_partial_LEF1              ------PGSDSKELPV--HS 510
TCF7_Homo_sapiens_V3                    NQQTDWCGPCRRKKKCIRYL 510
TCF7_Mus_musculus_V3                    NQQTDWCGPCRRKKKCIRYL 510
TCF7_Gallus_gallus_V3                   NQQTDWCGPCRRKKKCIRYL 510
TCF7_Taeniopygia_guttata_V3             NQQTDWCGPCRRKKKCIRYL 510
TCF7_Anolis_carolinensis_V3             NQQMDWCGPCRRKKKCIRYL 510
TCF7_Chrysemys_picta_bellii_V3          NQQTDWCGPCRRKKKCIRYL 510
TCF7_Xenopus_tropicalis_V3              NQQTDWCGPCRRKKKCIRYL 510
TCF7S_Xenopus_laevis_V3                 NQQTDWCGPCRRKKKCIRYL 510
TCF7_chr15_Acipenser_ruthenus_V3        NQQTDWCDPCRRKKKCIRYL 510
TCF7_chr29_Acipenser_ruthenus_V3        NQQTDWCGPCRRKKKCIRYL 510
TCF7_Latimeria_chalumnae_V3             NQQTDWCGPCRRKKKCIRYL 510
TCF7_Lepisosteus_oculatus_V3            NQQTDWCGPCRRKKKCIRYL 510
TCF7_Danio_rerio_V3                     NQQTDWCGPCRRKKKCIRYL 510
TCF7_Oryzias_latipes_V3                 NQQTDWCGPCRRKKKCIRYL 510
TCF7_Takifugu_rubripes_V3               NQQTDWCGPCRRKKKCIRYL 510
TCF7_Tetraodon_nigroviridis_V3          SQQTDWCGPCRRKKKCIRYL 510
Amblyraja_radiata_TCF7                  DQRTDWCDPCRKKKKCVRYM 510
Chiloscyllium_plagiosum_TCF7            DQRTDWCGPCRKKKKCIRYL 510
Scanicula_TCF7                          DQRTDWCGPCRKKKKCIRYL 510
Elphany_shark_partial_TCF7              DQRTNWCPPCRKKKKCIRFV 510
TCF7L1_Homo_sapiens                     -----GALASKSKKPCVQYL 510
TCF7L1_Mus_musculus                     -----GALASKSKKPCIQYL 510
TCF7L1_Gallus_gallus                    -----SVLPSRSKKPCAPHL 510
TCF7L1_Taeniopygia_guttata              -----GSLPSRSKKPCVPYL 510
TCF7L1_Anolis_carolinensis              -----NSVALKSKKPCVPYL 510
TCF7L1_Chrysemys_picta_bellii           -----SSLASKSKKPCVQYL 510
TCF7L1_Xenopus_tropicales               --------ITKTKKMCVQHL 510
TCF7L1L_Xenopus_laevis                  --------HTKTKKMCVQHL 510
TCF7L1S_Xenopus_laevis                  -------NYTKTKKMCVQHF 510
TCF7L1_chr41_Acipenser_ruthenus         ---LEDQFSSRNKKPCTQYM 510
TCF7L1_ste150_Acipenser_ruthenus        ---LEDQFSSRNKKPCTQYM 510
TCF7L1_Latimeria_chalumnae              -------LASRTKKQCIQYM 510
TCF7L1a_Lepisosteus_oculatus            ------NFSSRNKKQCVQYL 510
TCF7L1b_Danio_rerio                     -------FSMRSKKPCVQYL 510
TCF7L1_Oryzias_latipes                  -------FSIRSKKQCVQYL 510
TCF7L1_Takifugu_rubripes                -------FSIRSKKQCVQYL 510
TCF7L1_Tetraodon_nigroviridis           -------FSIRSKKQCVQYL 510
TCF7L1a_Danio_rerio                     ------NFSPQPKKQCVPYL 510
TCF7L1x_Tetraodon_nigroviridis          ------EGFYPRLMSFLSSV 510
TCF7L1x_Takifugu_rubripes               VPPEEKPHTQTAHTQPRPFM 510
TCF7L1x_Oryzias_latipes                 TKKPRQDPPPAHTSHARPHL 510
Scanicula_TCF7L1                        -SRDLSLPVSRRRLKCVQYL 510
Amblyraja_radiata_TCF7L1_partial        --PDASVPVAHRRVRSRSHV 510
Chiloscyllium_plagiosum_TCF7L1          -NRDLSLPVSRRLKCVQYL 510
Elphany_shark_partial_TCF7L1            -------------------- 510
TCF7L2_Homo_sapiens_V3                  DQQNNWCGPCRRKKKCVRYI 510
TCF7L2_Mus_musculus_V3                  DQQNNWCGPCRRKKKCVRYI 510
TCF7L2_Gallus_gallus_V3                 DQQNNWCGPCRRKKKCVRYI 510
TCF7L2_Taeniopygia_guttata_V3           DQQNNWCGPCRRKKKCVRYI 510
TCF7L2_Anolis_carolinensis_V3           DQQNNWCGPCRRKKKCVRYI 510
TCF7L2_Chrysemys_picta_bellii_V3        DQQNNWCGPCRRKKKCVRYI 510
TCF7L2_Xenopus_tropicalis_V3            DQQNNWCGPCRRKKKCIRYI 510
TCF7L2L_Xenopus_laevis_V3               DQQNNWCGPCRRKKKCIRYI 510
TCF7L2S_Xenopus_laevis_V3               DQQNNWCGPCRRKKKCIRYI 510
TCF7L2_chr7_Acipenser_ruthenus_V3       DQQNNWCGPCRRKKKCIRYI 510
TCF7L2_chr13_Acipenser_ruthenus_V3      DQQNNWCGPCRRKKKCIRYI 510
TCF7L2_Latimeria_chalumnae_V3           DQQNNWCGPCRRKKKCIRYI 510
TCF7L2_Lepisosteus_oculatus_V3          DQQNNWCGPCRRKKKCIRYI 510
TCF7L2_Danio_rerio_V3                   DQQNNWCGPCRRKKKCIRYI 510
TCF7L2_Tetraodon_nigroviridis_V3        DQQNNWCGPCRRKKKCIRYI 510
TCF7L2_Oryzias_latipes_V3               DQQNNWCGPCRRKKKCIRYI 510
TCF7L2_Takifugu_rubripes_V3             DQQNNWCGPCRRKKKCIRYI 510
Amblyraja_radiata_TCF7L2                DQQNNWCGPCRRKKKCIRYI 510
Chiloscyllium_plagiosum_TCF7L2          DQQNNWCGPCRRKKKCIRYI 510
Scanicula_TCF7L2                        DQQSLWCKPCRRKKKCIRYI 510
Elephant_shark_TCF7L2                   DQQNNWCGPCRRKKKCIRYI 510
Lamprey_TCF_chr41                       DQQNNWCSPCRRKKKCARYP 510
Pacific_lamprey_TCF_chr43               DQQNNWCSPCRRKKKCARYP 510
Lethenteron_reissneri_chr47_TCF         DQQNNWCSPCRRKKKCARYP 510
Lamprey_TCF_chr8                        EQQGDWCGPCRRKKKCIRNV 510
Pacific_lamprey_TCF_chr22               EQQGDWCGPCRRKKKCIRNL 510
Lethenteron_reissneri_chr34_TCF         EQQGDWCGPCRRKKKCIRNV 510
```

```
Lamprey_putative_TCF_chr68_partial      DQQNNWCGPCRRKKRCARYM 510
Ciona_robusta_TCF_v3                    EQQQLWCAPCRRKKKCIRYQ 510
Ciona_intestinalis_TCF                  EQQQLWCAPCRRKKKCIRYQ 510
Ciona_savignyi_TSA_based                EQQQLWCAPCRRKKKCIRYQ 510
Phallucia_fumigata_TCF                  EQQQLWCAPCRRKKKCIRYQ 510
Halocynthia_roretzi_TCF                 EQQQLWCAPCRRKKKCIRYQ 510
B_lanceolatum_TCF                       DQQQAWCKPCRRKKKCIRYI 510
B_belcheri_TCF                          DQQQAWCKPCRRKKKCIRYI 510
B_floridae_TCF                          DQQQAWCKPCRRKKKCIRYI 510
Saccoglossus_kowalevskii_TCF            DQQHHWCKPCRGRKKCIRYV 510
Ptychodera_flava_TCF_TSA                DQQHHWCKPCRRKKKCIRYI 510
Strongylocentrotus_purpuratus_TCF       DQQDFWCKPCRRKKKCIRYI 510
Asterias_rubens_TCF                     DQQEFWCKPCRRKKKCIRYI 510
Apostichopus_japonicus_TCF              DQQDFWCKPCRRKKKCIRFI 510
```
